# DNA repair in Mycoplasma gallisepticum

**DOI:** 10.1186/1471-2164-14-726

**Published:** 2013-10-23

**Authors:** Alexey Y Gorbachev, Gleb Y Fisunov, Mark Izraelson, Darya V Evsyutina, Pavel V Mazin, Dmitry G Alexeev, Olga V Pobeguts, Tatyana N Gorshkova, Sergey I Kovalchuk, Dmitry E Kamashev, Vadim M Govorun

**Affiliations:** 1 Research Institute of Physico-Chemical Medicine, Malaya Pirogovskaya 1a, Moscow 119992, Russian Federation; 2 Institute for Information Transmission Problems of the Russian Academy of Sciences, Bolshoy Karetny 19, Moscow 127994, Russian Federation; 3 Moscow Institute of Physics and Technology, Institutsky 9, Dolgoprudny 141700, Russian Federation; 4 Shemyakin–Ovchinnikov Institute of Bioorganic Chemistry of the Russian Academy of Sciences, Miklukho-Maklaya 16/10, Moscow 117997, Russian Federation

**Keywords:** SOS-response, Mycoplasma gallisepticum, DNA repair, Minimal cell, Mollicutes

## Abstract

**Background:**

DNA repair is essential for the maintenance of genome stability in all living beings. Genome size as well as the repertoire and abundance of DNA repair components may vary among prokaryotic species. The bacteria of the *Mollicutes* class feature a small genome size, absence of a cell wall, and a parasitic lifestyle. A small number of genes make *Mollicutes* a good model for a “minimal cell” concept.

**Results:**

In this work we studied the DNA repair system of *Mycoplasma gallisepticum* on genomic, transcriptional, and proteomic levels. We detected 18 out of 22 members of the DNA repair system on a protein level. We found that abundance of the respective mRNAs is less than one per cell. We studied transcriptional response of DNA repair genes of *M. gallisepticum* at stress conditions including heat, osmotic, peroxide stresses, tetracycline and ciprofloxacin treatment, stationary phase and heat stress in stationary phase.

**Conclusions:**

Based on comparative genomic study, we determined that the DNA repair system *M. gallisepticum* includes a sufficient set of proteins to provide a cell with functional nucleotide and base excision repair and mismatch repair. We identified SOS-response in *M. gallisepticum* on ciprofloxacin, which is a known SOS-inducer, tetracycline and heat stress in the absence of established regulators. Heat stress was found to be the strongest SOS-inducer. We found that upon transition to stationary phase of culture growth transcription of DNA repair genes decreases dramatically. Heat stress does not induce SOS-response in a stationary phase.

## Background

Genomic DNA is constantly subject to damage. This includes the misincorporation of nucleotides by DNA-polymerases and chemical modification either by endogenous metabolites or external compounds. DNA damage is a frequent event, and cell division is possible because most of the lesions are effectively repaired. The DNA repair mechanisms of bacteria are numerous and universal. The inactivation of single genes in the DNA repair system is generally not lethal because of a significant amount of redundancy among DNA repair pathways [1].

Bacteria of the class *Mollicutes* features a small though sufficient genome to grow on a cell-free medium and a low GC content (31% for *Mycoplasma gallisepticum*). Their genome size typically ranges from 580 thousand to 1.4 million bp. Most mollicute species are obligate parasites [2-4]. As a result, they are constantly exposed to stress conditions caused by host defense, including elevated temperature and reactive oxygen species [2]. It is interesting how organisms with reduced genomes retain genome stability under adverse conditions.

DNA repair systems are divided into groups based on mechanism of action. Mismatch repair (MMR) fix non-pairing bases, which originated from the misincorporation of nucleotides by DNA-polymerase [5]. Chemically modified nucleotides are restored by base excision repair (BER). It recognizes specific modifications by dedicated proteins and replaces modified nucleotides with the correct ones [6]. Nucleotide excision repair (NER) deals with a broad spectrum of DNA damage. It removes a section of the defective DNA chain and fills it based on a complementary chain [1]. DNA lesions that are substrate of the BER system can be restored by the NER system but in a less efficient manner [7]. Double strand breaks or crosslinks are removed by homologous recombination. However, this requires information from homologous DNA molecules (not from a complementary chain, as for BER and NER), which is time consuming and involves massive DNA synthesis [1,8]. Therefore, recombination activates as a part of an SOS-response only in cases of severe DNA damage [9]. SOS-response in *Mollicutes* is of particular interest due to absence of LexA repressor in all *Mollicutes*[10]. An SOS-response may involve a number of repair enzymes, but key players are recombination protein and error-prone polymerases [11]. In bacteria, an SOS-response is activated by massive DNA damage [12]. Activation of an SOS-response inevitably leads to elevation of a mutation rate as well [13], which is unwanted for a cell in normal conditions. Absence of LexA repressor in *Mollicutes* was considered evidence that an SOS-response is not functional in these bacteria [2,10].

Mismatch repair was considered most degenerated in mycoplasmas. Homologs of MutH, MutS and MutL proteins were not found in all *Mollicutes*. This led to the conclusion that mutation rate is elevated in mycoplasmas [14]; however, it was later shown that it does not differ from the one in *E. coli*[15,16]. It was shown recently that histone-like protein HU (Hup2) in *M. gallisepticum* is able to recognize mispaired bases in DNA in contrast to HU from *Acholeplasma laidlawii* with functional MMR [17,18]. It can also compensate for inactivation of hupAB proteins in *E. coli*[17]. This may indicate its role in mismatch repair.

In the current study, we attempted to characterize the DNA repair system of *Mycopalsma gallisepticum*. We identified members of different DNA repair pathways based on genomic data and publications. We quantified mRNA of respective proteins per cell in a set of adverse conditions and identified most of them on the protein level. We showed induction of SOS-response in adverse conditions on a transcriptional level in the absence of a known regulator.

## Results

### In silico DNA repair system reconstruction

#### Nucleotide excision repair

Nucleotide excision repair of *M. gallisepticum* encompasses all required members on the protein level (Tables 1 and 2). This includes UvrA (lesion binding), UvrB (local melt of DNA), UvrC (excision of damaged site), and UvrD-helicase (removal of damaged site), as well as DNA-ligase and DNA-polymerase [19]. The interesting question here is which polymerase is recruited for DNA repair because DNA-polymerase I is absent in *M. gallisepticum* as well as in most of *Mollicutes*. It seems that DNA-polymerase III serves both DNA replication and repair. Alternatively, the DNA repair function can be taken by DNA-polymerase IV.

**Table 1:** **Probable participants of the DNA repair system in *M. gallisepticum***

**DNA repair system**	**Gene name**	**Function**	**Presence in genome *M. gallisepticum***	**Copy number of mRNA per one genome**	**Presence in proteome**
All systems	*ligA*	DNA ligase	+	0.03	+
DNA methylation	*hsdM*	DNA-methyltransferase	+	nd	**-**
MMR	*Exo*	5′- 3′-exonuclease	+	nd	+
MMR	*hup2*	DNA-mismatch binding	+	0.4	+
MMR	*MGA_0793*	Putative vsr protein	+	nd	+
MMR	*MGA_0195*	Putative MutH analogue	+	nd	+
NER and SOS	*uvrA*	Excinuclease ABC subunit A	+	0.02	+
NER and SOS	*uvrB*	Excinuclease ABC subunit B	+	0.02	+
NER and SOS	*uvrC*	Excinuclease ABC subunit C	+	0.02	+
NER and MMR	*uvrD*	DNA helicase II	+	0.04	+
BER	*fpg (mutM)*	Formamidopyrimidine-DNA glycosylase	+	0.09	+
BER	*Ung*	Uracil-DNA glycosylase	+	0.01	**-**
BER	*Nfo*	endonuclease IV	+	0.08	+
Recombination and SOS	*recA*	Recombinase RecA	+	0.01	+
Recombination and SOS	*ruvA*	Holliday junction ATP-dependent DNA helicase subunit A	+	0.01	**-**
Recombination and SOS	*ruvB*	Holliday junction ATP-dependent DNA helicase subunit B	+	0.01	**-**
Recombination	*Smc*	Chromosome cohesion	+	nd	**+**
Recombination and SOS	*recR*	Recombination protein RecR	+	0.02	**+**
Recombination	*MGA_0016*	Recombination protein RecO	+	nd	**-**
Recombination	*recU*	Holliday junction resolvase	+	nd	**-**
Recombination	*MGA_0836*	Holliday junction resolvase	+	nd	**+**
SOS	*dinB*	DNA-polymerase IV	+	0.01	**+**

nd – no data; “**-**” – not detected.

**Table 2:** DNA repair proteins identification by LC-MS/MS

**NCBI ID**	**Protein name**	Unused score*	**ProteinPilot score**	**The number of unique peptides (with the reliability of identification > = 95%)**	**Coverage protein sequence by unique peptides (with the reliability of identification > = 95%)**
gi|31541218	SMC (Cohesin)	84.01	84.01	55	54.43
gi|284811881	UvrA (Excinuclease ABC subunit B)	75.8	75.8	50	55.67
gi|284811888	MGA_0793 (DNA helicase, contain vsr-domain)	62.07	62.07	41	36.41
gi|284812070	UvrD (DNA helicase II)	51.65	51.65	27	47.54
gi|284811857	UvrB (Excinuclease ABC subunit B)	41.75	41.75	24	39.01
gi|31541419	Nfo (endonuclease IV)	31.18	31.18	17	63.04
gi|284812280	LigA (DNA ligase)	24.41	24.83	13	23.08
gi|284812220	RecR (recombinase RecR)	24.06	24.06	15	72.82
gi|284811982	MutM (Formamidopyrimidine-DNA glycosylase)	20.02	20.02	10	41.97
gi|31541551	MGA_0195 (contain endonuclease type II domain)	18.9	18.9	18	49.18
gi|284812101	Hup2 (histone-like protein)	16.7	18.1	23	71.72
gi|284811981	Exo (5′- 3′-exonuclease)	12.01	12.01	8	37.85
gi|284812049	UvrC (Excinuclease ABC subunit C)	6.19	6.19	4	5.672
gi|31541441	MGA_0016 (recombinase RecO)	5.29	5.29	3	19.5
gi|31541171	putative Holliday junction resolvase	4.9	4.9	5	31.69
gi|284812207	DinB (DNA-polymerase IV)	4.05	4.05	2	5.985
gi|31541522	RecA (recombinase RecА)	4.01	4.01	2	8.547
gi|31541659	Ung (Uracil-DNA glycosylase)	3.13	3.86	3	13.85

*The protein identification algorithm receives the value score for the protein as the sum of the scores for all its related peptides (the score in the program is a direct derivative of ProteinPilot reliability of identification). In the case where a peptide is common to the two proteins, its contribution to the score of the protein, which has a lower accuracy of the identification (less than the total score) will be less than the maximum possible value, calculated based on the reliability of his identification. Thus, the value of the unused score reflects the use of the same peptides (or rather the spectrum on the basis of which were identified peptides) in the identification of other proteins. The closer the value of the unused score to the total score of the protein, the more specific and accurate is this identification.

### Base excision repair

**Figure 1:**
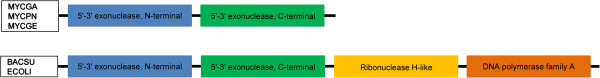
**Domain organization of Exo protein of *M. gallisepticum*, *M. genitalium, *** **and *M. pneumoniae *** **in comparison to DNA polymerase I of *E. coli *** **and *B. subtilis*.**

Base excision repair starts for glycosylases, which recognize specific lesions in DNA. *M. gallisepticum* have two enzymes of this type, including uracil- (Ung) and formamidopyrimidine-DNA-glycosylases (MutM). The next player is AP-endonuclease, which nicks DNA at 5′ position from the AP-site. The only AP-endonuclease in *Mollicutes* is Nfo, which belongs to the endonuclease IV family. This family of endonucleases features 3′-phosphatase activity but lacks 5′ -phosphatase and 3′-5′ exonuclease activity in contrast to the endonuclease III family [6]. In well-studied bacteria like *E. coli,* the AP-site is removed and repaired by DNA-polymerase I (PolA), which has 5′-3′ exonuclease activity. In *M. gallisepticum* and most of *Mollicutes,* this mechanism is likely substituted with DNA-polymerase III and Exo protein, which is an exonuclease, homologous to the 5′-3′ exonuclease domain of PolA. Exo seems to originate from PolA as a result of truncation with the loss of the polymerase domain (Figure 1). All components of BER except uracil-glycosylase were identified on the protein level [20-22].

### Mismatch repair

We found two additional proteins that can play a role in mismatch repair. They are MGA_0195 and MGA_0793. The first one contains an endonuclease domain that belongs to the same superfamily as the endonuclease domain of MutH. The second one contains a vsr domain, which takes part in the repair of mispaired guanines. MGA_0195 and MGA_0793 were identified on the protein level (Table 2). A hallmark of *M. gallisepticum* is the absence of known exonucleases (Exo I, Exo VII, RecJ, Exo X [5]) of the MMR pathway. However, missing activity can be compensated by exonuclease Exo, a truncated PolA.

We identified one DNA methylation enzyme: HsdM. This is site-specific methylase that methylate adenine in position six.

### Recombinational repair system

The genome of *M. gallisepticum* carries genes that encode key proteins required for recombination - recombinase RecA, RecR, and RecO (MGA_0016), as well as genes *ruvA* and *ruvB*, encoding a DNA helicase RuvAB (involved in the migration of the DNA chains), as well as two genes encoding enzymes that allow Holliday junction resolution - DNA resolvase RecU and MGA_0836. In addition, there is a gene *smc*, encoding Smc-cohesin capable of implementing cohesion after the replication of chromosomes, thus participating in the recombinational repair [23].

### SOS response system

The following participants of SOS-system were found in the genome of *M. gallisepticum*: recombinase A (*recA*), recombinase R (*recR*), helicase complex (*ruvA, ruvB*), nuclease-helicase complex UvrABC, and DNA-dependent DNA polymerase IV (*dinB*) which homologous to the mutagenic DNA pol IV of *E. coli*. In bacteria, this polymerase (along with DNA polymerase V, which is absent in all members of the class *Mollicutes*) is able to use damaged DNA template [24]. At the same time, we didn’t find any homologs of known regulators of the bacterial SOS-system (LexA [25], HdiR [26]) in any of the analyzed genomes of *Mollicutes*. We found all of the prospective members of the SOS-response system at the mRNA level; while at the protein level, we were unable to identify only helicase RuvAB (Table 2).

### Presence of DNA-repair transcripts in the cell

Comparative analysis based only on genomic data does not allow us to draw conclusions about genes expression and the functional activity of the proteins encoded by them. To test the activity of the annotated genes, we carried out a quantitative analysis of transcription levels for the genes of the repair system by quantitative reverse transcription PCR (droplet-digital PCR and real-time PCR - see Methods). We also counted the number of transcripts copies per single bacterial genome. The results of the expression assay for the studied genes at the mRNA and protein levels are shown in Table 1. Transcription was detected for all of analyzed genes.

It should be noted that the number of copies of mRNA varies from one per one hundred (*recA, ruvA, ung, dinB*) to one per 2.5 copies of genomic DNA (*hup2*). If we assume that each cell contains, on average, one copy of genomic DNA, the presence of transcripts is rather low, and in most of the cells there are no transcripts of the genes’ encoding repair system proteins.

### Presence of DNA-repair proteins in the cell

In order to assess the presence of repair systems members at the protein level, we used the methods of liquid chromatography-mass spectrometry for the comprehensive identification of all the proteins in the cells of *M. gallisepticum*. We have identified 561 proteins in total (data not shown) by using the following selection criteria: the number of unique peptides for protein - at least two - and a global FDR of 1% (based on the analysis in PSPEP, with threshold unused score for the protein equaled 0.4). Thus 17,221 unique peptides have been identified (global FDR 1% PSPEP). It should be noted that due to the fact that the algorithm Paragon, used for searching, works on the basis of probability factors, the total number of peptides includes not only non-modified tryptic peptides but semi-tryptic and non-tryptic peptides as well, and peptides containing all possible amino acid modifications considered in the default search algorithm of Paragon.

Analysis of the presence of the DNA repair proteins (Table 2) showed the presence of most of them in the mycoplasma’s proteome - 18 proteins for 22 genes are listed in Table 2. All proteins in Table 2 were unique according to the groups of spectrums using the Pro Group algorithm.

### Repair system genes response on stress (T, NaCl, H_2_O_2_, stationary phase of growth, antibiotics)

**Figure 2:**
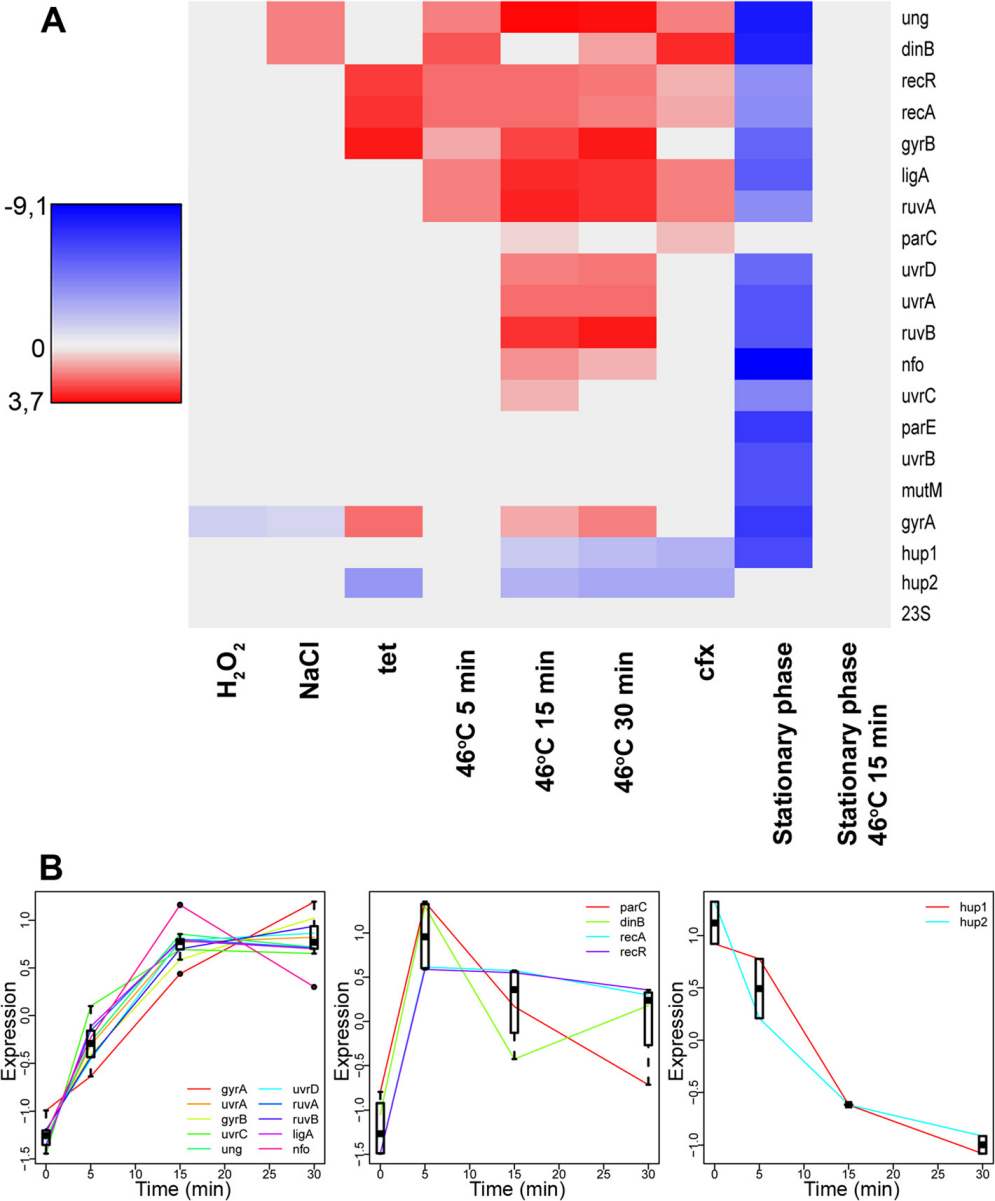
**Transcription of genes of *M. gallisepticum *involved in DNA repair and homeostasis. A** – Transcription profiles of DNA repair and homeostasis genes under different conditions. 23S rRNA gene was used as a reference. Genes with q-values less than 0.05 were considered to change the expression significantly. Colors indicate direction (red for upregulation, blue for downregulation) and level of expression change (log2). Gray indicates no statistically significant change in expression (t-test, BH-correction, q-value > 0.05); tet – tetracycline treatment, cfx – ciprofloxacin treatment. **B** – Kinetics of the transcriptional response during heat stress. Each box shows a different expression pattern (see Methods). Individual genes are shown by lines. Only genes that significantly change expression with at least one stress duration are shown. Gene expressions were normalized to mean zero and variance one before plotting. Distributions of normalized expressions for given stress duration and pattern are shown by boxes. Genes with the same rank in all conditions were considered to have similar expression patterns. Only genes with significant expression changes between control and at least one stress duration were used.

According to the literature data, the SOS-repair system of *Mollicutes* have undergone reduction [10]. In order to understand whether an SOS-response takes place in *M. gallisepticum,* we decided to test changes in the levels of mRNAs of the genes’ encoding proteins of the repair system in response to stress influences. We exposed the cells to sub-lethal stress (see Methods) - temperature, osmotic and peroxide stress, and antibiotics. Such effects were selected because they are physiologically common for parasites, as the interaction with the host organism cells encounter reactions of inflammation: heat, immune system peroxide attack, and antibiotics treatment. Figure 2A shows the color map, representing the change of the transcription profiles for the genes’ encoding DNA repair proteins. Raw data are presented in Additional file 1.

Ciprofloxacin is known as an SOS-inducer in different bacteria [13,27]. It was used as a reference condition to identify members of SOS-response in *M. gallisepticum*. Ciprofloxacin treatment resulted in the upregulation of *recA*, *recR*, *ung*, *ruvA*, *ligA*, *parC,* and *dinB*. Remarkably, the 15-fold induction of error-prone polymerase (*dinB*) was the strongest effect of the ciprofloxacin treatment. Tetracycline treatment induced upregulation of the SOS-response participants *recA* and *recR* but not *dinB*. DNA gyrase (*gyrA* and *gyrB*), which is a target of ciprofloxacin, was induced by tetracycline but not ciprofloxacin. Osmotic stress induced the upregulation of *dinB* and *ung* but not *recA* and *recR*. Peroxide stress had little effect on the transcription of genes involved in DNA repair. Heat stress invoked the strongest response among all types of stress. Response to heat stess includes genes involved in responses to ciprofloxacin, *uvrABCD*, *nfo* glycosylase, and DNA gyrase (*gyrA* and *gyrB*). It may be concluded that the stress response system of *M. gallisepticum* consists of several regulons.

The stationary growth phase differs remarkably from the exponential phase in terms of gene expression as well as stress response. The stationary phase demonstrates a significant downregulation of most of the studied genes. Heat stress in the stationary phase did not result in the change of expression of any studied gene in contrast to its exponential phase. It may indicate that the transcription process in the stationary phase is modulated by some global regulator, like an alternative sigma factor.

The transcription profile for the repair system genes in *M. gallisepticum* under heat shock conditions (Figure 2B) suggests three groups of genes. The first group is characterized by a gradual growth of the transcription level and includes the majority of the genes. The second group includes the genes that respond to the heat stress by dramatical increasing of the transcription level following by decreasing or stagnation of the last one. The third group includes two genes of histone-like proteins *hup1* and *hup2*. They are characterized by a steady decline of transcription (Figure 2B).

## Discussion

The study of the mycoplasma repair system, as bacteria with a reduced genome, gives an indication of the minimum number of genes required to maintain genomic stability. Comparative analysis shows that the repair system of *M. gallisepticum* includes fewer genes than the one of *E. coli*. However, if we give preference to functional rather than numerical parameters, we can see the presence of the key elements of all major repair systems in the absence of overlapping units.

**Figure 3:**
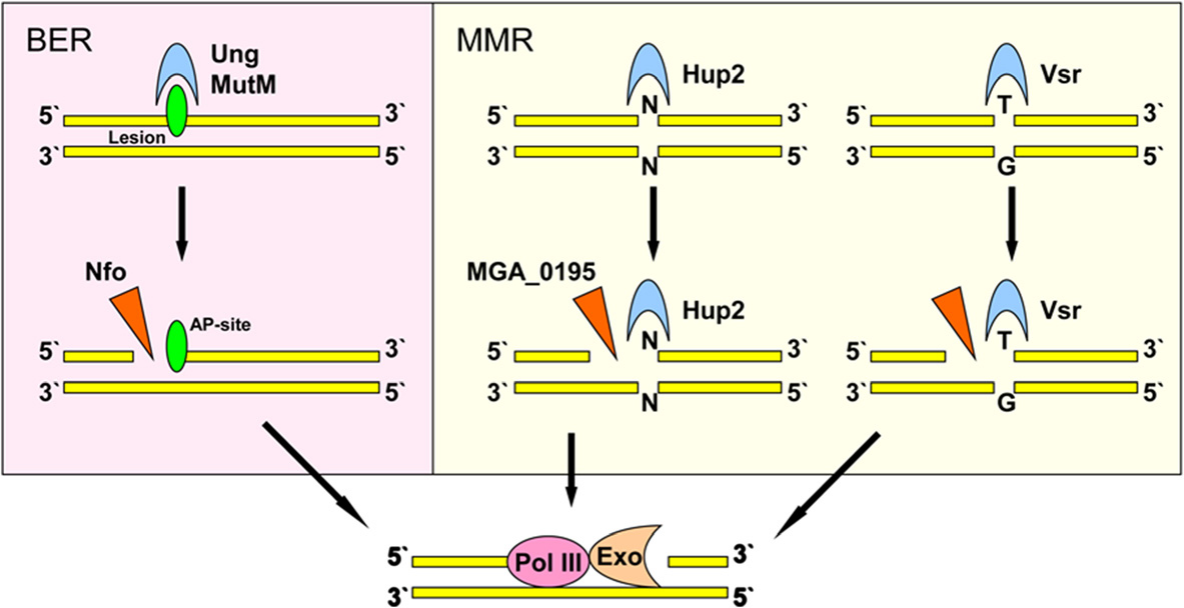
**Hypothetical model of the MMR and BER systems in *M. gallisepticum.***

The results show that the possible composition of the *M. gallisepticum* repair system may be greater than it was assumed up until now. In particular, we found several previously unknown mycoplasma proteins. One of them - a protein MGA_0793 - was annotated as a hypothetical protein of unknown function. Results of the alignment revealed homology with the Vsr protein, which was involved in the mismatch repair of DNA containing an unpaired guanine in *E. coli*[28]. Interestingly, the HU (Hup2) protein identified earlier in *M. gallisepticum*, capable of binding DNA mismatches, does not bind unpaired T-G, A-G, and G-G base pairs [17]. The Figure 3 shows a hypothetical model of the MMR system in *M. gallisepticum.*

We identified one DNA methylation enzyme: HsdM. This is site-specific methylase that methylate adenine in position six. DNA methylation is required for MMR pathways to distinguish the correct strand to use as a template [5]. It was shown that methylation occurs in mycoplasmas [29-31]. However dam-methyltransferase required for the methylation of GATC sites is absent in *M. gallisepticum*, the discrimination of the chains may be due to the interaction of the repair complex with the B-subunit of DNA polymerase III in replication - such a mechanism was previously shown for a number of microorganisms (Gram-positive bacteria) [1]. Thus we can at least say that *M. gallisepticum* has an enzyme capable of recognizing and binding DNA mismatches, as well as an enzyme hypothetically capable of the excision of the damaged DNA fragment. But the question remains, which enzyme of mycoplasma is a functional analog of MutH protein, which is required to make a single-strand break in the DNA being repaired.

We found that *M. gallisepticum* has the full pathway of nucleotide excision repair and recombination of DNA. From all of DNA recombination participants, only RecR have been previously found in *Mycoplasma gallisepticum* at the protein level [20-22]. In this study, we identified RecA and Smc proteins as well (Table 2).

Of particular interest for the analysis of gene excision is the base excision repair system, as it has always been represented by a large number of proteins, each of which recognizes a different type of damage. There are three known types of damage: oxidation, alkylation, and deamination. DNA glycosylase MutM in *E. coli*, whose homolog is present in the genome of *M. gallisepticum*, corrects the most frequently occurring DNA damage caused by endogenous oxidative stress [32]. Uracil-glycosylase removes uracil, which arises spontaneously by the deamination of cytosine or by erroneous inclusion during replication. Mollicutes have only one of two AP-endonucleases - endonuclease IV. It’s interesting that endonuclease IV (Nfo) has an additional activity - it recognizes oxidized bases (hydroxycytosine, dihydroxyuracil, and dihydroxythymine) and makes a single-stranded gap upstream of the damage, which is used as a primer for the repair by polymerase and ligase [7]. The Figure 3 shows a hypothetical model of the BER system in *M. gallisepticum.*

Assuming that *Mollicutes* are bacteria with a minimal genome and are capable of self-reproduction, we come to the conclusion that they possess the number of repair system proteins that are necessary and sufficient for life in the cell-free medium.

The results of the gene transcription assay indicate that the mRNA molecules are not present in every cell in the population (see Table 1). These data are consistent with the literature and may be associated with the long lifetime of the functional protein in contrast to the mRNA [33]. At the proteomic level, we were able to identify a large portion (80%) of repair proteins, including DinB and RecA, members of SOS-response, which is an additional indication of the functionality of the repair system in *M. gallisepticum*.

An interesting result of the transcription-profiling assay was the induction of SOS-response genes in different shocks. This fact interested us for several reasons. First of all, such a response has not been shown previously in *Mollicutes*, including large-scale studies of transcription responses in *M. pneumoniae*[34], which is one of the closest relatives of *M. gallisepticum* according to phylogenetic studies [35]. The other interest is that the genomes of all members of the class *Mollicutes* don’t have any known regulator of the SOS-response system, and therefore several authors consider the SOS-system as non-functional in mycoplasmas [2,10]. However, our results are consistent with published data obtained by the transcriptional analysis of non-relatives to mycoplasmas bacteria, where the regulatory system of the SOS-response was described [1,36,37]. These observations may indicate the functionality of the SOS-response on the one hand and the presence of an unknown regulator on the other. In favor of the hypothesis of the presence of such a regulator, this may also indicate the presence of a number of genes in the genome, whose protein products, according to the annotation, have sequence-specific DNA-binding domains and could potentially act as transcription factors (data not shown). In addition rapid induction at the level of gene transcription in the second group (Figure 2B) may be indicative of the presence of a repressor that acts similar to the previously described LexA-repressor in the *E. coli*. The subsequent decline of the mRNA of *parC* and *dinB* genes may be due to the presence of a negative feedback regulation. This is particularly likely in the case of *dinB*, which encodes an alternative DNA polymerase as its activation can lead to dangerously high levels of mutagenesis. The increase in mRNA levels of most of the repair system genes may be a consequence of the global regulatory mechanism rather than the result of a single transcription factor.

Demonstrated here, the induction of DNA polymerase IV in different types of stress is consistent with the literature and may be a mechanism of adaptation to stress by increasing endogenous levels of mutagenesis [9,13,38,39].

## Conclusions

Based on comparative genomic study, we determined that the DNA repair system *M. gallisepticum* includes a sufficient set of proteins to provide a cell with functional nucleotide and base excision repair and mismatch repair. We identified SOS-response in *M. gallisepticum* on ciprofloxacin, which is a known SOS-inducer, tetracycline and heat stress in the absence of established regulators. Heat stress was found to be the strongest SOS-inducer. We found that upon transition to stationary phase of culture growth transcription of DNA repair genes decreases dramatically. Heat stress does not unduce SOS-response in a stationary phase.

## Methods

### Strains and conditions

*Mycoplasma gallisepticum S6* was cultivated on a liquid medium [40] at 37°C for 12 and 24 hours for exponential and stationary phases, respectively (Additional file 2). Cells were passaged twice for 24 hours, starting from frozen culture prior to the experiment. Cells were passaged in 1:10 dilution. Cells were harvested by centrifugation at 8 000 g and 4°C for 10 min.

### DNA extraction

Cells were harvested as described above and lysed with CTAB buffer (2% CTAB, 100 mM Tris–HCl, pH = 8.0, 20 mM EDTA, 1.4 M NaCl) at 60°C for 30 min with subsequent chloroform extraction (1:1) and isopropanol precipitation (1:1) with the addition of 10% v/v sodium acetate [41].

### RNA extraction and cDNA synthesis

Total RNA was extracted directly from the cell culture using TrizolLS (Invitrogen) reagent (1:3 cell culture: TrizolLS) with subsequent chloroform extraction (1:5 chloroform: TrizolLS) and isopropanol precipitation (1:1). RNA was treated by DNase I (Thermo Scientific) and used for cDNA synthesis with H-minus Mu-MLV reverse transcriptase (Thermo Scientific).

### Real-time PCR and droplet-digital PCR

Real-time PCR was performed using iQ SYBR Green Supermix (Bio-Rad) and CFX96™ Real-Time PCR Detection System (Bio-Rad) PCR machine. Droplet digital PCR allows direct quantification of DNA molecules in a sample [42]. It was performed using ddPCR™ Supermix for Probes (Bio-Rad) and QX100 system (droplet generator and droplet reader) along with a DNA Engine Tetrad 2 (Bio-Rad) PCR machine. Real-time and ddPCR data was analyzed with CFX Manager and QuantaSoft (Bio-Rad) software, respectively. Primers and probes are listed in Additional file 3. All PCR experiments were carried out in three biological and two technical replicates.

### Quantification of RNA copy number per cell

The copy number of RNA and DNA molecules was measured using ddPCR. Final data was normalized with the respect of cell culture volume which results in a copy number of RNA and DNA per unit of culture volume. The copy number of RNA per cell was estimated as a ratio of RNA per DNA copy number based on the assumption that cells have one copy of genomic DNA.

### Determination of sub-lethal conditions

Sub-lethal conditions were determined as conditions when stressful conditions are maximal but most of the cells are still viable. Working under such conditions ensures the maximal response in the absence of massive cell death, which can significantly affect results. Cell viability was estimated by the determination of colony forming units that are formed by cells after stress. Sub-lethal conditions for different stresses were found to be the following: 46°C for 1 hour for heat stress, 1.2 M NaCl for 1 hour, 0.02% H_2_O_2_ for 1 hour, 2 μg of ciprofloxacin for 4 hours, and 8 μg of tetracycline for 1 hour.

### Protein extraction and 1D electrophoresis

Cells harvested as described were washed twice in a wash buffer (150 mM NaCl, 50 mM Tris–HCl, 2 mM MgCl_2_, pH = 7.4). Cells were lysed in 20 μl of 1% SDS in 100 mM NH_4_HCO_3_ and incubated in an ultrasonic bath for 15 min with subsequent centrifugation at 10000 g at 4°С for 5 min. Supernatant was taken, and protein concentration was determined by Bicinchoninic acid protein assay kit (Sigma). 20 μl of 2x Laemmli reagents were then added, and samples were incubated at 95°С for 5 min. Then 50 μg of protein was loaded to polyacrylamide gel (10×0.1 cm, 12% polyacrylamide), and electrophoresis was performed according to Laemmli [43] (10 mA current). Electrophoresis was stopped when the front dye reached 1.5 cm in separating gel.

### Trypsinolysis in polyacrylamide gel

The polyacrylamide gel was fixed in a fixation buffer (20% CH_3_OH and 10% CH_3_COOH) for 30 min and washed twice in H_2_0. The gel was cut in pieces 1×1 mm, transferred into tubes, and treated with 10 mM DTT and 100 mM NH_4_HCO_3_ for 30 min at 56°C. Then proteins were alkylated with 55 mM iodoacetamide in 100 mM NH_4_HCO_3_ for 20 min in the dark. Then water was removed from gel pieces by the addition of 100% acetonitrile.

Dehydrated samples were treated with a 150 μl trypsin solution (40 mM NH_4_HCO_3_, 10% acetonitrile, 20 ng/μl Trypsin Gold, mass spectrometry grade, Promega). Samples were incubated for 60 min at 40°C and for 16–18 h. at 37°C. Peptides were extracted one time by 5% formic acid and two times by 50% acetonitrile with 5% formic acid. Extracts were joined and dried on a vacuum centrifuge at 45°C. Precipitate was diluted in 50 μl of 5% acetonitrile with 0.1% formic acid.

### Chromato-mass-spectrometry

Peptides were analyzed using a TripleTOF 5600+ (ABSciex) mass-spectrometer with NanoSpray III ion source and NanoLC Ultra 2D + chromatograph (Eksigent). Chromatographic separation was carried out in the gradient of acetonitrile in water (5 to 40% of acetonitrile in 120 min) with 0.1% formic acid on 75×150 μm columns with Phenomenex Luna C18 3 μm sorbent and a flow rate of 300 nL/min.

The IDA mode of a mass-spectrometer was used to analyze peptides. Based on the first MS1 spectrum (mass range for analysis and subsequent ion selection for MS2 analysis was 300–1250 m/z, signal accumulation was 250 ms), 50 parent ions with maximum intensity in the current spectrum were chosen for subsequent MS/MS analysis (resolution of quadrupole UNIT was 0.7 Da, measurement mass range was 200–1800 m/z, optimization of ion beam focus was to obtain maximal sensitivity, signal accumulation was 50 ms for each parent ion). Nitrogen was used for collision dissociation with fixed average energy of 40 V. Collision energy was linearly increased from 25 to 55 V during signal accumulation time (50 ms). Parental ions that had already been analyzed were excluded from analysis for 15 sec.

### Analysis of mass-spectrometry data

Raw data was analyzed with ProteinPilot 4.5 revision 1656 (ABSciex) using search algorithm Paragon 4.5.0.0, revision 1654 (ABSciex), and standard search settings to search against a database of all proteins of *M. gallisepticum S6* (genbank id: AFFR01000000). The following parameters were used for this search: alkylation of cysteine by iodoacetamide, trypsin digestion, TripleTOF 5600 equipment, and a deep search with additional statistical analysis of results reliability. Specters were grouped with default settings by a ProGroup algorithm build-in to ProteinPilot. The statistical analysis of results reliability (and identification of threshold value of unused score) was carried out by a ProteomicS Performance Evaluation Pipeline Software (PSPEP) algorithm build-in to ProteinPilot.

The mass spectrometry proteomics data have been deposited to the ProteomeXchange Consortium (http://proteomecentral.proteomexchange.org) via the PRIDE partner repository [44] with the dataset identifier PXD000249 and DOI 10.6019/PXD000249.

### Comparative analysis and in-silico reconstruction of DNA repair system

We have used Gene Ontology database (http://www.geneontology.org/) and literature analysis, we compiled the list of all the genes involved in DNA repair in E. coli and (or) B. subtilis. For all of the selected genes we found homologues in the genome of M. gallisepticum using blastn and blastp algorithms (e-value <1e-25). We aligned selected proteins of *M. gallisepticum* with its respective homologues of *E. coli* and (or) B. subtilis in order to analyze the amino acid substitutions in the active center with the help of ClustalW2 algorithm (http://www.ebi.ac.uk/Tools/msa/clustalw2/) and PDB database (http://www.rcsb.org/pdb/home/home.do). The results are shown in the Table 1 and the Additional file 4.

### Statistical analysis of mRNA expression levels by quantitative RT-PCR (qRT-PCR)

Data presented are the average of three individual biological experiments with calculated standard deviation (Additional file 1); within each experiment, technical duplicates were performed. The level of each mRNA (log2) identified in each condition was compared with the control, which was exponential growth phase for all conditions and stationary phase for stationary phase heat stress. To identify the significance of a mRNA level change, we used a t-test with multiple hypothesis testing correction by the Benjamini-Hochberg procedure [45].

### Identification of genes expression patterns during heat stress

The Gene expression levels (log2) during 15 and 30 min heat stress were averaged. After that, a rank of each mRNA was calculated for control, 5 min and an average 15–30 min stress. The condition with the maximal expression gained a rank of “1”, and the condition with the minimal expression gained a rank of “3”.

## Competing interests

The authors declare that they have no competing interests.

## Authors’ contributions

AG designed the project, performed experimental and bioinformatics data and wrote the paper; GF designed the project, performed bioinformatics, qRT-PCR data and wrote the paper; MI performed qRT-PCR data and corrected the paper; DE performed functional protein domain search; PM performed bioinformatics and statistical calculations and wrote the paper; DA performed calculation for mass-spectrometry data and corrected the paper; OP performed protein extraction and sample preparation for chromato-mass-spectrometry; TG performed trypsinolysis for chromato-mass-spectrometry; SK performed mass-spectrometry data and wrote the paper; DK designed the project and wrote the paper, VG designed and supervised the project and wrote the paper. All authors read and approved the final manuscript.

## Supplementary Material

Additional file 1 Determination of culture growth rate. Click here for file

Additional file 2 Synthetic oligonucleotides. Click here for file

Additional file 3 **The results of transcriptional profiling by qRT-PCR.** Data presented are the average of three individual experiments; within each experiment, technical duplicates were performed. Click here for file

Additional file 4 **DNA repair proteins of *M. gallisepticum *** **alignments with *E. coli *** **and (or) *B. subtilis *** **homologs.** Click here for file
