# Supplementary material for: DNA repair in Mycoplasma gallisepticum
Source: BMC Genomics. 2013 Oct 23;14:726. doi: 10.1186/1471-2164-14-726 (PMC4007778; doi:10.1186/1471-2164-14-726)
Supplement: Additional file 1 — Determination of culture growth rate. [file 1471-2164-14-726-S1.docx]

**Additional file 1**

**Synthetic oligonucleotides**

| **Ген** | **Forward primers 5′-3′** | **Reverse primers 5′-3′** | **Molecular probes 5′-3′** |
| --- | --- | --- | --- |
| ***clpB*** | GAAAGATTACAGGCAAAAGGTG | GCGCTCTTCCTCTTAGTACTGC | FAM-ATCAAATTGTCGGTAAATTCGCTCAATTCT-BHQ1 |
| ***16SrRNA*** | CCGTGTCTCAGTCCCATTGT | GCAAGTCGATCGGATGTAGC | FAM-CGGGTTAACTGAAATTCTTTCACGACCAATAGTAAC-BHQ1 |
| ***23SrRNA*** | CATCCCGTAAGTTCGCAAGA | ACCCGTACCTGGATTTCACC | FAM-ATGGAGTGACGGAGAAGGTTAATGCATC-BHQ1 |
| ***hup2*** | ATTTGTGCGAATCTACTGCA | AATGGCAGACGAAACTAACC | FAM-TTGAAACAGTTAGTTTGATTTTCTTCCCTGCTG-BHQ1 |
| ***hup1*** | AGGCGGAATTCTAGTTTGTG | ATTATCGCTGAATGTACTGGAG | FAM-CGGGTTAACTGAAATTCTTTCACGACCAATAGTAAC-BHQ1 |
| ***parE*** | CATGACTTCCACCTTCAGAG | CATTTATTGCCAAAGACGGGA | FAM-CAGCGTTTTGAATTGAGACTTGGATTAGTTGG-BHQ1 |
| ***uvrB*** | TGTGTTGTCGGAATTAACCT | ATCGCTTTAGTCATCTCATCAG | FAM-TGACGCTGACAAACCTGGTTATTTCAGAAG-BHQ1 |
| ***parC*** | AAACAACCCTAATCCCAAACC | CTTAAACTAATGCCAGGACCA | FAM-AGTTTGTTTGCTTCTAAGATCTCTTCAATCGAACG-BHQ1 |
| ***uvrD*** | GTGTTGTAAGTTATGGTCCGA | CAGTGGTTTGACGATCATAGG | FAM-TGGTGTTAATCGTTTAAATGTAGCAATCACTCGAG-BHQ1 |
| ***uvrC*** | TATGAAGTGATCTATCGCAGGT | AGTGACGATCTTATCTGTTTGG | FAM-TTGTTGATCTACCTGATCTGATTATTCTAGATGGTGG-BHQ1 |
| ***gyrA*** | TACCACTTGAACCATTTGCT | CCCTCATAGTGATAGTGCGA | FAM-CAATCGGTTCTTGTTCTGAAGCATCATAGTTATCA-BHQ1 |
| ***dinB*** | CCAAGTCTAATGGTTCTATCGT | ATCGCTTGCAGATATCTCAC | FAM-TAGCTAGAAAATTTGGCGTTCGGTCTGC-BHQ1 |
| ***uvrA*** | AAACCCTGCTTCACTTACTG | TTTCCACTACCAGAAACTCC | FAM-TCCACCCAGGGAACGGACAAAAGATC-BHQ1 |
| ***recA*** | TGGTATGAGAATCAACTACCTG | GTAATCCCGAGGTTACAACTG | FAM-TCGCTTTTGATCTAATCCCTATTGCATTATTTGC-BHQ1 |
| ***nei*** | GAACGTCATGTGTTAGTACGA | GCATAAATATTACCGATCCCAG | FAM-TTGAACTGCACTATCATGACACAAGAAGATTTGG-BHQ1 |
| ***nfo*** | ATTATGTTGTTCACGCTCCT | ATCAGCCATTGTTTCAAGAC | FAM-AGCTAATGGTGATTCAACTAAACGAGAACGC-BHQ1 |
| ***recR*** | TAATTTGCTGTTGTTTGCCC | GTCAGGGTTGATCAAATCGT | FAM-ACTTACCATGATAAGCTTGGGATTCTTCAATCAC-BHQ1 |
| ***ligA*** | CTTAGGGATGATCTCAGCAG | GTTACACCTCTAAGTTTCCCA | FAM-AGTGATAATCGAACCTTCTAATTCAATTGGGGC-BHQ1 |
| ***gyrB*** | GTGTTGTGTCAGAACTATTGGA | GACCAGATTTAGCTGAACCA | FAM-CTAAAGCAGTTTCAGCAATGTTTTCACGTAAACG-BHQ1 |
| ***ruvB*** | TTGTGGGTGATAACGAATGG | AGCCAGAATAAAGCATTAGACC | FAM-TTCATTAGCAATAATCTGTGCCAACGATGTTTTG-BHQ1 |
| ***ruvA*** | TGCTTCAATCACGATCTCTG | GCACTGTTAACGATCAATGG | FAM-AGGTCTTTGCTATCATCAATTTTCTCATCGTCATC-BHQ1 |
| ***ung*** | TGATTAATTCTTCCCAACCCAG | GTAATCATTGGTCAAGATCCGT | FAM-TTGATTAATAACACCCCTTGGTTAGCCCAG-BHQ1 |
