# Supplementary material for: DNA repair in Mycoplasma gallisepticum
Source: BMC Genomics. 2013 Oct 23;14:726. doi: 10.1186/1471-2164-14-726 (PMC4007778; doi:10.1186/1471-2164-14-726)
Supplement: Additional file 2 — Synthetic oligonucleotides. [file 1471-2164-14-726-S2.docx]

**Additional file 2**

**The results of transcriptional profiling by qRT-PCR.**

Data presented are the average of three individual experiments; within each experiment, technical duplicates were performed.

| **Gene** | **Control**  **(log-phase), log2** | **St. dev.** | **Ciprofloxacin, log2** | **St. dev.** | **Tetracycline, log2** | **St. dev.** | **46С 5 min, log2** | **St. dev.** | **46С 15 min, log2** | **St. dev.** |
| --- | --- | --- | --- | --- | --- | --- | --- | --- | --- | --- |
| ***clpB*** | 23,71 | 0,22 | 25,84 | 0,20 | 23,05 | 0,07 | 19,18 | 0,35 | 19,32 | 0,68 |
| ***16S*** | 10,07 | 0,37 | 10,20 | 0,04 | 9,89 | 0,14 | 9,69 | 0,05 | 11,09 | 0,68 |
| ***23S*** | 11,28 | 0,04 | 11,29 | 0,01 | 11,33 | 0,04 | 11,29 | 0,01 | 11,17 | 0,07 |
| ***hup2*** | 24,88 | 0,45 | 27,64 | 0,44 | 28,26 | 0,17 | 26,21 | 0,14 | 27,21 | 0,72 |
| ***hup1*** | 24,45 | 0,20 | 26,87 | 0,45 | 24,46 | 0,16 | 24,59 | 0,47 | 25,95 | 0,20 |
| ***parE*** | 25,67 | 1,31 | 27,28 | 0,32 | 25,41 | 0,15 | 26,61 | 0,58 | 26,60 | 1,28 |
| ***uvrB*** | 27,39 | 0,33 | 26,80 | 0,56 | 26,74 | 0,09 | 27,01 | 0,50 | 28,02 | 0,31 |
| ***parC*** | 27,27 | 0,07 | 26,37 | 0,09 | 26,22 | 0,77 | 25,99 | 0,54 | 26,69 | 0,12 |
| ***uvrD*** | 25,84 | 0,41 | 25,74 | 0,14 | 24,42 | 0,54 | 25,14 | 0,44 | 23,95 | 0,09 |
| ***uvrC*** | 27,40 | 0,30 | 26,81 | 0,22 | 26,06 | 0,69 | 26,67 | 0,38 | 26,39 | 0,03 |
| ***gyrA*** | 25,55 | 0,11 | 25,98 | 0,31 | 23,49 | 0,32 | 25,24 | 0,27 | 24,32 | 0,38 |
| ***dinB*** | 29,18 | 0,11 | 25,99 | 0,21 | 27,20 | 0,56 | 26,72 | 0,44 | 28,52 | 0,34 |
| ***uvrA*** | 26,06 | 0,31 | 27,87 | 1,48 | 24,80 | 1,31 | 25,08 | 0,23 | 23,99 | 0,06 |
| ***recA*** | 27,52 | 0,24 | 26,38 | 0,23 | 24,54 | 0,09 | 25,40 | 0,28 | 25,44 | 0,20 |
| ***nei*** | 27,08 | 0,43 | 27,75 | 0,12 | 25,03 | 1,19 | 27,45 | 0,33 | 26,82 | 0,35 |
| ***nfo*** | 26,25 | 0,27 | 27,10 | 0,63 | 23,98 | 1,06 | 25,59 | 0,11 | 24,66 | 0,33 |
| ***recR*** | 27,48 | 0,28 | 26,43 | 0,06 | 24,60 | 0,64 | 25,33 | 0,34 | 25,38 | 0,31 |
| ***ligA*** | 27,54 | 0,17 | 25,72 | 0,27 | 24,87 | 0,87 | 25,68 | 0,53 | 24,34 | 0,15 |
| ***gyrB*** | 26,71 | 0,19 | 25,81 | 0,32 | 23,34 | 0,41 | 25,50 | 0,18 | 24,00 | 0,44 |
| ***ruvB*** | 26,90 | 0,52 | 25,94 | 0,24 | 24,30 | 1,54 | 25,70 | 0,36 | 23,90 | 0,16 |
| ***ruvA*** | 27,75 | 0,21 | 25,90 | 0,27 | 25,04 | 1,58 | 25,98 | 0,33 | 24,54 | 0,52 |
| ***ung*** | 27,55 | 0,17 | 25,70 | 0,15 | 23,04 | 1,27 | 25,78 | 0,17 | 23,82 | 0,37 |
|  |  |  |  |  |  |  |  |  |  |  |
| **Gene** | **46С 30 min, log2** | **St. dev.** | **H2O2, log2** | **St. dev.** | **NaCl, log2** | **St. dev.** | **Stationary phase, log2** | **St. dev.** | **46С 15 min stationary phase, log2** | **St. dev.** |
| ***clpB*** | 21,03 | 0,11 | 23,22 | 0,03 | 21,62 | 0,31 | 19,83 | 0,59 | 19,66 | 0,55 |
| ***16S*** | 13,05 | 0,80 | 9,85 | 0,04 | 10,26 | 0,01 | 10,57 | 0,04 | 10,37 | 0,24 |
| ***23S*** | 11,98 | 0,69 | 11,27 | 0,01 | 11,29 | 0,01 | 11,26 | 0,03 | 10,89 | 0,42 |
| ***hup2*** | 27,57 | 0,13 | 25,94 | 0,72 | 25,63 | 0,05 | 24,50 | 1,33 | 24,51 | 1,40 |
| ***hup1*** | 26,40 | 0,02 | 26,15 | 0,27 | 25,47 | 0,21 | 30,77 | 1,16 | 30,92 | 1,00 |
| ***parE*** | 26,39 | 0,02 | 25,35 | 0,31 | 24,48 | 0,15 | 32,57 | 2,24 | 32,96 | 2,37 |
| ***uvrB*** | 28,16 | 0,01 | 27,04 | 0,33 | 27,24 | 0,47 | 33,47 | 1,60 | 33,48 | 1,22 |
| ***parC*** | 27,21 | 0,06 | 27,58 | 0,15 | 27,34 | 0,14 | 35,79 | 4,21 | 36,68 | 3,75 |
| ***uvrD*** | 23,88 | 0,23 | 26,69 | 0,24 | 26,11 | 0,07 | 30,99 | 1,55 | 31,58 | 1,56 |
| ***uvrC*** | 26,41 | 0,17 | 27,26 | 0,38 | 26,87 | 0,08 | 31,45 | 1,20 | 32,28 | 1,21 |
| ***gyrA*** | 23,68 | 0,19 | 26,83 | 0,03 | 26,62 | 0,17 | 32,56 | 1,34 | 33,56 | 1,21 |
| ***dinB*** | 27,90 | 0,09 | 29,56 | 0,14 | 27,38 | 0,28 | 37,03 | 1,22 | 37,49 | 1,13 |
| ***uvrA*** | 23,93 | 0,12 | 26,08 | 0,22 | 25,90 | 0,00 | 31,96 | 1,14 | 32,48 | 1,46 |
| ***recA*** | 25,72 | 0,31 | 26,34 | 0,12 | 26,41 | 0,16 | 31,27 | 1,20 | 32,41 | 1,30 |
| ***nei*** | 25,97 | 0,29 | 27,45 | 0,41 | 26,17 | 0,23 | 33,14 | 0,18 | 33,54 | 0,84 |
| ***nfo*** | 25,23 | 0,10 | 25,19 | 0,15 | 25,06 | 0,27 | 35,37 | 1,26 | 34,87 | 1,03 |
| ***recR*** | 25,58 | 0,25 | 26,34 | 0,04 | 26,23 | 0,14 | 31,17 | 1,21 | 32,21 | 1,30 |
| ***ligA*** | 24,46 | 0,22 | 27,57 | 0,07 | 27,19 | 0,26 | 33,11 | 1,15 | 33,62 | 1,14 |
| ***gyrB*** | 23,33 | 0,13 | 27,68 | 0,20 | 26,23 | 0,33 | 31,98 | 0,89 | 32,60 | 0,91 |
| ***ruvB*** | 23,52 | 0,12 | 27,31 | 0,08 | 25,92 | 0,11 | 32,76 | 0,59 | 32,47 | 0,65 |
| ***ruvA*** | 24,68 | 0,07 | 28,00 | 0,13 | 26,74 | 0,16 | 31,59 | 0,36 | 31,94 | 0,26 |
| ***ung*** | 24,06 | 0,09 | 27,44 | 0,10 | 25,75 | 0,07 | 35,69 | 0,96 | 35,71 | 1,92 |

*St. dev. – standard deviation
