# Supplementary material for: DNA repair in Mycoplasma gallisepticum
Source: BMC Genomics. 2013 Oct 23;14:726. doi: 10.1186/1471-2164-14-726 (PMC4007778; doi:10.1186/1471-2164-14-726)

**Additional file 3**

Determination of the growth kinetics of the liquid culture *M. gallisepticum*. The red curve represents the accumulation of ribosomal RNA, blue - genomic DNA. The horizontal axis shows the time in hours and the vertical - the relative abundance of RNA or DNA on a logarithmic scale (log2). An increase of one unit corresponds to a twofold increase.


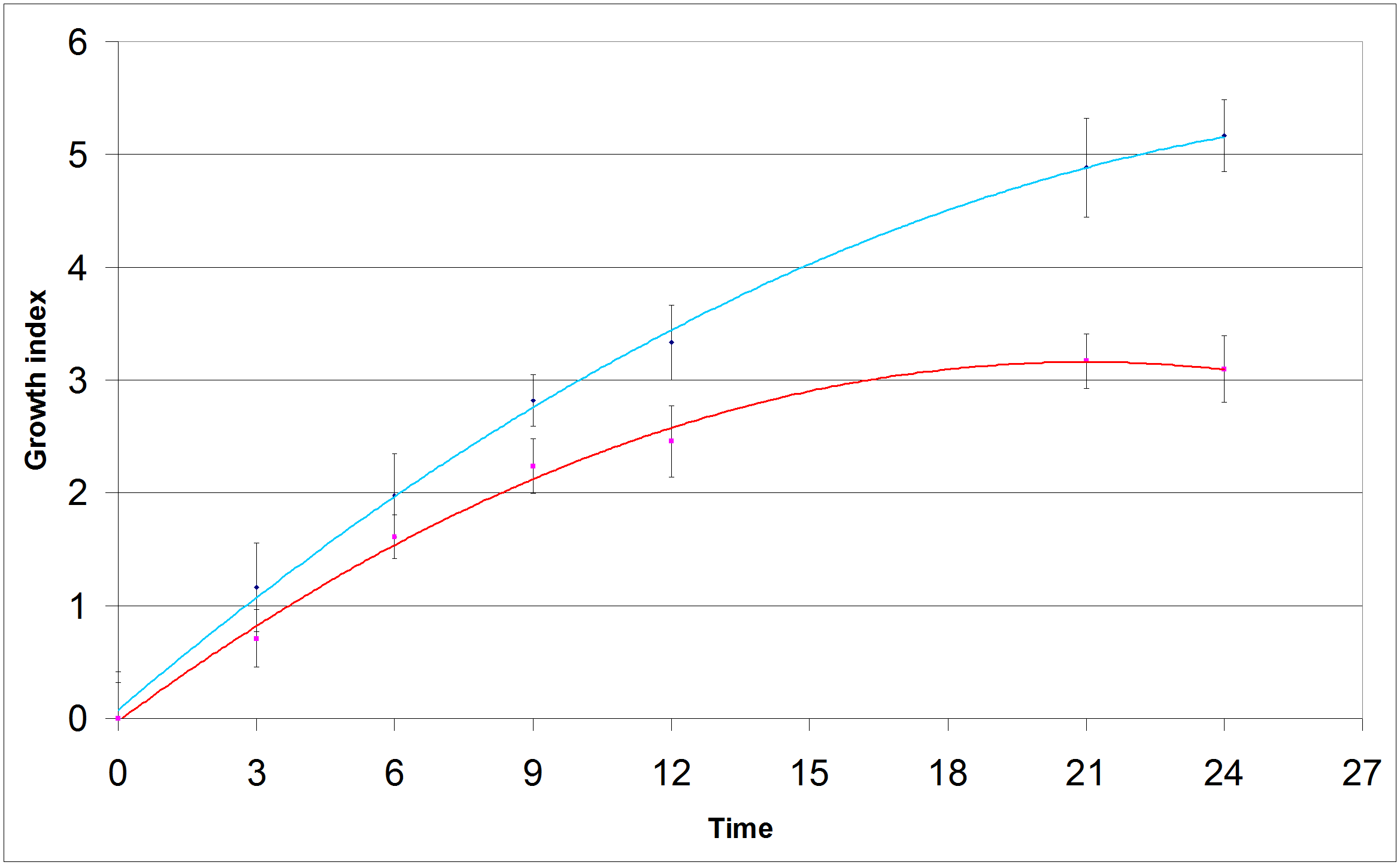

Supplement: Additional file 3 — The results of transcriptional profiling by qRT-PCR. Data presented are the average of three individual experiments; within each experiment, technical duplicates were performed. [file 1471-2164-14-726-S3.docx]
