# Supplementary material for: DNA repair in Mycoplasma gallisepticum
Source: BMC Genomics. 2013 Oct 23;14:726. doi: 10.1186/1471-2164-14-726 (PMC4007778; doi:10.1186/1471-2164-14-726)
Supplement: Additional file 4 — DNA repair proteins of M. gallisepticum alignments with E. coli and (or) B. subtilis homologs. [file 1471-2164-14-726-S4.docx]

**Additional file 4.**

**DNA repair proteins of *M. gallisepticum* alignments with *E. coli* and (or) *B. subtilis* homologs**

1. **DNA ligase (gene name *ligA*)**

>sp|P15042|DNLJ_ECOLI DNA ligase OS=Escherichia coli (strain K12) GN=ligA PE=1 SV=2

MESIEQQLTELRTTLRHHEYLYHVMDAPEIPDAEYDRLMRELRELETKHPELITPDSPTQ

RVGAAPLAAFSQIRHEVPMLSLDNVFDEESFLAFNKRVQDRLKNNEKVTWCCELKLDGLA

VSILYENGVLVSAATRGDGTTGEDITSNVRTIRAIPLKLHGENIPARLEVRGEVFLPQAG

FEKINEDARRTGGKVFANPRNAAAGSLRQLDPRITAKRPLTFFCYGVGVLEGGELPDTHL

GRLLQFKKWGLPVSDRVTLCESAEEVLAFYHKVEEDRPTLGFDIDGVVIKVNSLAQQEQL

GFVARAPRWAVAFKFPAQEQMTFVRDVEFQVGRTGAITPVARLEPVHVAGVLVSNATLHN

ADEIERLGLRIGDKVVIRRAGDVIPQVVNVVLSERPEDTREVVFPTHCPVCGSDVERVEG

EAVARCTGGLICGAQRKESLKHFVSRRAMDVDGMGDKIIDQLVEKEYVHTPADLFKLTAG

KLTGLERMGPKSAQNVVNALEKAKETTFARFLYALGIREVGEATAAGLAAYFGTLEALEA

ASIEELQKVPDVGIVVASHVHNFFAEESNRNVISELLAEGVHWPAPIVINAEEIDSPFAG

KTVVLTGSLSQMSRDDAKARLVELGAKVAGSVSKKTDLVIAGEAAGSKLAKAQELGIEVI

DEAEMLRLLGS

>sp|O31498|DNLJ_BACSU DNA ligase OS=Bacillus subtilis (strain 168) GN=ligA PE=3 SV=1

MDKETAKQRAEELRRTINKYSYEYYTLDEPSVPDAEYDRLMQELIAIEEEHPDLRTPDSP

TQRVGGAVLEAFQKVTHGTPMLSLGNAFNADDLRDFDRRVRQSVGDDVAYNVELKIDGLA

VSLRYEDGYFVRGATRGDGTTGEDITENLKTIRNIPLKMNRELSIEVRGEAYMPKRSFEA

LNEERIKNEEEPFANPRNAAAGSLRQLDPKIAAKRNLDIFVYSIAELDEMGVETQSQGLD

FLDELGFKTNQERKKCGSIEEVITLIDELQAKRADLPYEIDGIVIKVDSLDQQEELGFTA

KSPRWAIAYKFPAEEVVTKLLDIELNVGRTGVITPTAILEPVKVAGTTVSRASLHNEDLI

KEKDIRILDKVVVKKAGDIIPEVVNVLVDQRTGEEKEFSMPTECPECGSELVRIEGEVAL

RCINPECPAQIREGLIHFVSRNAMNIDGLGERVITQLFEENLVRNVADLYKLTKERVIQL

ERMGEKSTENLISSIQKSKENSLERLLFGLGIRFIGSKAAKTLAMHFESLENLKKASKEE

LLAVDEIGEKMADAVITYFHKEEMLELLNELQELGVNTLYKGPKKVKAEDSDSYFAGKTI

VLTGKLEELSRNEAKAQIEALGGKLTGSVSKNTDLVIAGEAAGSKLTKAQELNIEVWNEE

QLMGELKK

>sp|Q7NAF8|DNLJ_MYCGA DNA ligase OS=Mycoplasma gallisepticum (strain R(low / passage 15 / clone 2)) GN=ligA PE=3 SV=2

MIEKKNESIKEIIEDLVKKLTKWEHEYYVLSNPSVSDEVYDNTYRTLLGYERKYPQYVLS

YSPTQRVGSSISNKFIKVKHDYLMLSLGNCFNFEELLNFNENIAKISKQEDNPYVLEPKI

DGLSISLIYLDGVLSEALTRGDGVFGESVIANIKTIKTIPLKINTTIKKIVIRGEVYVSN

QDFEAINASRDEDKKFANSRNYASGSLRNIDVSEVAKRKLNAFFYYIPNAYELGFETQYQ

VIQQLKEWGFNVAKEIKLFSNIKELYTSLKELENNKNKLDYRIDGAVIKYNNFKDYEIIG

YTSKFPKWAIAYKFAPTQVQTQLKDIILNVGRTGKLTFVAQLAPIELEGSIITYATLHNL

EYINDLDIRINDYVYLIKAAEIIPKVIGVNLDKRPNNAKKLEFDYNCPSCHQPLVKKPEE

VDWYCIYDQCKQKQLQYLIYYCSKPIMNIEGLSESTLALFFNTKVNDVIRECNELITNQN

VSEDLSLFKLLDNEEQTFVNSVLDIYQLERYKEIIIKPWLKKGFSKSSLKYNFRFQEKSF

DKLINSINESKNRELYRLLAALNIKYIGIATAKSIANTYHDIDQLKNLTVEDYMRLADIS

SITANSLFSFFSDEKNWELIEQLKTLSINTKDEINNDLVDSSSIYYDKKFVITGSFSISR

NDIIKKLSLKYKIKFVSGVSKNVDFVLAGNSPTAKKINQAKVLNIPIIQEEIWNK

sp|P15042|DNLJ_ECOLI -----MESIEQQLTELRTTLRHHEYLYHVMDAPEIPDAEYDRLMRELRELETKHPELITP 55

sp|O31498|DNLJ_BACSU ---MDKETAKQRAEELRRTINKYSYEYYTLDEPSVPDAEYDRLMQELIAIEEEHPDLRTP 57

sp|Q7NAF8|DNLJ_MYCGA MIEKKNESIKEIIEDLVKKLTKWEHEYYVLSNPSVSDEVYDNTYRTLLGYERKYPQYVLS 60

*: :: :* .: : .: *:.:. *.:.* **. : * * ::*: .

sp|P15042|DNLJ_ECOLI DSPTQRVGAAPLAAFSQIRHEVPMLSLDNVFDEESFLAFNKRVQDRLKNNEKVTWCCELK 115

sp|O31498|DNLJ_BACSU DSPTQRVGGAVLEAFQKVTHGTPMLSLGNAFNADDLRDFDRRVRQSVG--DDVAYNVELK 115

sp|Q7NAF8|DNLJ_MYCGA YSPTQRVGSSISNKFIKVKHDYLMLSLGNCFNFEELLNFNENIAKISKQ-EDNPYVLEPK 119

*******.: * :: * ****.* *: :.: *:..: . :. .: * *

sp|P15042|DNLJ_ECOLI LDGLAVSILYENGVLVSAATRGDGTTGEDITSNVRTIRAIPLKLHGENIPARLEVRGEVF 175

sp|O31498|DNLJ_BACSU IDGLAVSLRYEDGYFVRGATRGDGTTGEDITENLKTIRNIPLKMNRELS---IEVRGEAY 172

sp|Q7NAF8|DNLJ_MYCGA IDGLSISLIYLDGVLSEALTRGDGVFGESVIANIKTIKTIPLKINTTIK--KIVIRGEVY 177

:***::*: * :* : . *****. **.: *::**: ****:: : :***.:

sp|P15042|DNLJ_ECOLI LPQAGFEKINEDARRTGGKVFANPRNAAAGSLRQLDPRITAKRPLTFFCYGVGVLEGGEL 235

sp|O31498|DNLJ_BACSU MPKRSFEALNEERIKNEEEPFANPRNAAAGSLRQLDPKIAAKRNLDIFVYSIAELDEMGV 232

sp|Q7NAF8|DNLJ_MYCGA VSNQDFEAIN--ASRDEDKKFANSRNYASGSLRNIDVSEVAKRKLNAFFYYIPNAYELGF 235

:.: .** :* : : ***.** *:****::* .*** * * * : .

sp|P15042|DNLJ_ECOLI PDTHLGRLLQFKKWGLPVSDRVTLCESAEEVLAFYHKVEEDRPTLGFDIDGVVIKVNSLA 295

sp|O31498|DNLJ_BACSU ETQSQG-LDFLDELGFKTNQERKKCGSIEEVITLIDELQAKRADLPYEIDGIVIKVDSLD 291

sp|Q7NAF8|DNLJ_MYCGA ETQYQV-IQQLKEWGFNVAKEIKLFSNIKELYTSLKELENNKNKLDYRIDGAVIKYNNFK 294

: :.: *: . .. . . :*: : .::: .: * : *** *** :.:

sp|P15042|DNLJ_ECOLI QQEQLGFVARAPRWAVAFKFPAQEQMTFVRDVEFQVGRTGAITPVARLEPVHVAGVLVSN 355

sp|O31498|DNLJ_BACSU QQEELGFTAKSPRWAIAYKFPAEEVVTKLLDIELNVGRTGVITPTAILEPVKVAGTTVSR 351

sp|Q7NAF8|DNLJ_MYCGA DYEIIGYTSKFPKWAIAYKFAPTQVQTQLKDIILNVGRTGKLTFVAQLAPIELEGSIITY 354

: * :*:.:: *:**:*:**.. : * : *: ::***** :* .* * *:.: * ::

sp|P15042|DNLJ_ECOLI ATLHNADEIERLGLRIGDKVVIRRAGDVIPQVVNVVLSERPEDTREVVFPTHCPVCGSDV 415

sp|O31498|DNLJ_BACSU ASLHNEDLIKEKDIRILDKVVVKKAGDIIPEVVNVLVDQRTGEEKEFSMPTECPECGSEL 411

sp|Q7NAF8|DNLJ_MYCGA ATLHNLEYINDLDIRINDYVYLIKAAEIIPKVIGVNLDKRPNNAKKLEFDYNCPSCHQPL 414

*:*** : *: .:** * * : :*.::**:*:.* :.:*. : ::. : .** * . :

sp|P15042|DNLJ_ECOLI ERVEGEAVARCTGGLICGAQRKESLKHFVSRRAMDVDGMGDKIIDQLV------------ 463

sp|O31498|DNLJ_BACSU VRIEGEVALRCIN-PECPAQIREGLIHFVSRNAMNIDGLGERVITQLF------------ 458

sp|Q7NAF8|DNLJ_MYCGA VKKPEEVDWYCIY-DQCKQKQLQYLIYYCSKPIMNIEGLSESTLALFFNTKVNDVIRECN 473

: *. * * : : * :: *: *:::*:.: : :.

sp|P15042|DNLJ_ECOLI ---------------------EKEYVHTPADLFKLTAGKLTGLE---------------- 486

sp|O31498|DNLJ_BACSU ---------------------EENLVRNVADLYKLTKERVIQLE---------------- 481

sp|Q7NAF8|DNLJ_MYCGA ELITNQNVSEDLSLFKLLDNEEQTFVNSVLDIYQLERYKEIIIKPWLKKGFSKSSLKYNF 533

*: *.. *:::* : ::

sp|P15042|DNLJ_ECOLI RMGPKSAQNVVNALEKAKETTFARFLYALGIREVGEATAAGLAAYFGTLEALEAASIEEL 546

sp|O31498|DNLJ_BACSU RMGEKSTENLISSIQKSKENSLERLLFGLGIRFIGSKAAKTLAMHFESLENLKKASKEEL 541

sp|Q7NAF8|DNLJ_MYCGA RFQEKSFDKLINSINESKNRELYRLLAALNIKYIGIATAKSIANTYHDIDQLKNLTVEDY 593

*: ** ::::.:::::*: : *:* .*.*: :* :* :* : :: *: : *:

sp|P15042|DNLJ_ECOLI QKVPDVGIVVASHVHNFFAEESNRNVISELLAEGVH--WPAPIVINAEEIDSPFAGKTVV 604

sp|O31498|DNLJ_BACSU LAVDEIGEKMADAVITYFHKEEMLELLNELQELGVNTLYKGPKKVKAEDSDSYFAGKTIV 601

sp|Q7NAF8|DNLJ_MYCGA MRLADISSITANSLFSFFSDEKNWELIEQLKTLSIN--TKDEINNDLVDSSSIYYDKKFV 651

: ::. *. : .:* .*. :::.:* .:: . : .* : .*..*

sp|P15042|DNLJ_ECOLI LTGSLSQMSRDDAKARLVELGAKVAGSVSKKTDLVIAG-EAAGSKLAKAQELGIEVIDEA 663

sp|O31498|DNLJ_BACSU LTGKLEELSRNEAKAQIEALGGKLTGSVSKNTDLVIAG-EAAGSKLTKAQELNIEVWNEE 660

sp|Q7NAF8|DNLJ_MYCGA ITGSFSISRNDIIKKLSLKYKIKFVSGVSKNVDFVLAGNSPTAKKINQAKVLNIPIIQEE 711

:**.:. .: * *....***:.*:*:** ..:..*: :*: *.* : :*

sp|P15042|DNLJ_ECOLI EMLRLLGS 671

sp|O31498|DNLJ_BACSU QLMGELKK 668

sp|Q7NAF8|DNLJ_MYCGA IWNK---- 715

| **Organism** | **Active site (N6-AMP-lysine intermediate)** | **Metal binding (Zn)** | **Binding site (NAD)** | **Site (Interaction with target DNA)** |
| --- | --- | --- | --- | --- |
| E. coli (strain K12) | K115 | 408, 411, 426, 432 | 113,136, 173, 290, 314 | 487, 492 |
| B. subtilis (strain 168) | K115 | 404, 407, 422, 427 | 113, 136, 170, 286, 310 | No information |
| M. gallisepticum (strain R(low / passage 15 / clone 2)) | K119 | 407, 410, 425, 430 | 117, 140, 175, 289, 313 | No information |

1. **DNA-methyltransferase (gene name *hsdM*)**

Gene is absent in *B. subtilis* (strain 168) genome.

>sp|P08957|T1MK_ECOLI Type I restriction enzyme EcoKI M protein OS=Escherichia coli (strain K12) GN=hsdM PE=1 SV=1

MNNNDLVAKLWKLCDNLRDGGVSYQNYVNELASLLFLKMCKETGQEAEYLPEGYRWDDLK

SRIGQEQLQFYRKMLVHLGEDDKKLVQAVFHNVSTTITEPKQITALVSNMDSLDWYNGAH

GKSRDDFGDMYEGLLQKNANETKSGAGQYFTPRPLIKTIIHLLKPQPREVVQDPAAGTAG

FLIEADRYVKSQTNDLDDLDGDTQDFQIHRAFIGLELVPGTRRLALMNCLLHDIEGNLDH

GGAIRLGNTLGSDGENLPKAHIVATNPPFGSAAGTNITRTFVHPTSNKQLCFMQHIIETL

HPGGRAAVVVPDNVLFEGGKGTDIRRDLMDKCHLHTILRLPTGIFYAQGVKTNVLFFTKG

TVANPNQDKNCTDDVWVYDLRTNMPSFGKRTPFTDEHLQPFERVYGEDPHGLSPRTEGEW

SFNAEETEVADSEENKNTDQHLATSRWRKFSREWIRTAKSDSLDISWLKDKDSIDADSLP

EPDVLAAEAMGELVQALSELDALMRELGASDEADLQRQLLEEAFGGVKE

>tr|Q7NAH5|Q7NAH5_MYCGA Type I restriction-modification system methyltransferase (M) subunit OS=Mycoplasma gallisepticum (strain R(low / passage 15 / clone 2)) GN=hsdM PE=4 SV=2

MTKQELAREIWAMANEMRGNIEANDYKDYILGFLFYKYLSDKQDEYFASKNVVKDEDKKQ

YLVALAEADKKGIASIIHKCKKDLGYYIAYENLFSTWIKNYNPGDDLSDKVSTALNSFER

SILEKYEESFKDIFKDLQAGIQKLGNTAYERSEAIWNICNLINKIPITSKQDYDILGFVY

EYLISMFAANAGKKAGEFYTPHEVSQLMSVIAANHLKGLKNVSIYDPTSGSLLITLGREL

KKIDKNVKIQYYAQEVIDTTYNITRMNLLMNDVHSVNMFAKCGDTLKEDWPFVYEEQKYK

SKRTDAVVSNPPYSLAWNTENKENDPRFRYGLAPKSKSELAFLLHSLYHLEDHGILTIVL

PHGVLFRGGSELQIRQNLISHDHIDAIIGLPSNIFFGTGIPTIIMVLKRSKTKKEKNNVL

FIDASKYFTKEGNKNKLQSSDIVRIYDAFSAREDIPGFARVVSHEEIKANEYNLNIPKYI

DLVDNGDNHNLYSSIFSGIPHNDIDKLSDFWSTFPTLKKALLNDNGKNYQLKDHDVEKVI

SNNEEVKKYLSDFNKSLESLRTYFKKSLIDTDLNVIDLYNVYQQFLDKIQSVLKSYKLLD

YYQAFQLFDNEWTIIENDLKVIKSADNKNSFDTIRELKEHDSSTISAKADKKLVSKNTTY

GIYQTPVIPFEFVTKLKFNNQLEALEINSNKVEEINARLEELLNEVAGYESDVVNNFYKK

EENKLNFDEIKKQLKNLSVVAKSQPESVEALLVEALSIDKEKRALNSAIRKAKLQLEKDT

IQAYSKLTDEEAKTLLCLKWIDPLITAISKLTKYKIENLASELNRLDKKYIDKLSDLEVQ

IIQTQNSLIELINQLEGPDLDMEGLNELKKILGKK

| **Organism** | **Binding site (S-adenosyl-L-methionine)** | **Active site** |
| --- | --- | --- |
| E. coli (strain K12) | E216 | No information |
| M. gallisepticum (strain R(low / passage 15 / clone 2)) | No information | No information |

sp|P08957|T1MK_ECOLI MNNNDLVAKLWKLCDNLRDGGVSYQNYVNELASLLFLKMCKETG------ 44

tr|Q7NAH5|Q7NAH5_MYCGA MTKQELAREIWAMANEMR-GNIEANDYKDYILGFLFYKYLSDKQDEYFAS 49

*.:::*. ::* :.:::* *.:. ::* : : .:** * .:.

sp|P08957|T1MK_ECOLI -------------------------------------------QEAEYLP 51

tr|Q7NAH5|Q7NAH5_MYCGA KNVVKDEDKKQYLVALAEADKKGIASIIHKCKKDLGYYIAYENLFSTWIK 99

: ::

sp|P08957|T1MK_ECOLI EGYRWDDLKSRIGQEQLQFYRKMLVHLGEDDKKLVQAVFHNVSTT-ITEP 100

tr|Q7NAH5|Q7NAH5_MYCGA NYNPGDDLSDKVSTALNSFERSILEKYEESFKDIFKDLQAGIQKLGNTAY 149

: ***..::. .* *.:* : *. *.:.: : .:.. *

sp|P08957|T1MK_ECOLI KQITALVSNMDSLDWYNGAHGKSRDDFGDMYEGLLQKNANETKSGAGQYF 150

tr|Q7NAH5|Q7NAH5_MYCGA ERSEAIWNICNLINKIPITSKQDYDILGFVYEYLISMFAANAGKKAGEFY 199

:: *: . : :: : :. * :* :** *:. * :: . **:::

sp|P08957|T1MK_ECOLI TPRPLIKTIIHLLKPQPREVVQDPAAGTAGFLIEADRYVKSQTNDLDDLD 200

tr|Q7NAH5|Q7NAH5_MYCGA TPHEVSQLMSVIAANHLKGLKNVSIYDPT-----SGSLLITLGRELKKID 244

**: : : : : : : : : . ..: :. : : .:*..:*

sp|P08957|T1MK_ECOLI GDTQDFQIHRAFIGLELVPGTRRLALMNCLLHDIEGN---LDHGGAIRLG 247

tr|Q7NAH5|Q7NAH5_MYCGA KN-----VKIQYYAQEVIDTTYNITRMNLLMNDVHSVNMFAKCGDTLKED 289

: :: : . *:: * .:: ** *::*:.. . *.::: .

sp|P08957|T1MK_ECOLI NTLGSDGENLP--KAHIVATNPPFGSAAGTN------ITRTFVHPTSNKQ 289

tr|Q7NAH5|Q7NAH5_MYCGA WPFVYEEQKYKSKRTDAVVSNPPYSLAWNTENKENDPRFRYGLAPKSKSE 339

.: : :: ::. *.:***:. * .*: * : *.*:.:

sp|P08957|T1MK_ECOLI LCFMQHIIETLHPGGRAAVVVPDNVLFEGGKGTDIRRDLMDKCHLHTILR 339

tr|Q7NAH5|Q7NAH5_MYCGA LAFLLHSLYHLEDHGILTIVLPHGVLFRGGSELQIRQNLISHDHIDAIIG 389

*.*: * : *. * ::*:*..***.**. :**::*:.: *:.:*:

sp|P08957|T1MK_ECOLI LPTGIFYAQGVKTNVLFFTKG------------------TVANPNQDKNC 371

tr|Q7NAH5|Q7NAH5_MYCGA LPSNIFFGTGIPTIIMVLKRSKTKKEKNNVLFIDASKYFTKEGNKNKLQS 439

**:.**:. *: * ::.:.:. * . ::. :.

sp|P08957|T1MK_ECOLI TDDVWVYDLRTN---MPSFGKRTPFTDEHLQPFER--------VYGEDPH 410

tr|Q7NAH5|Q7NAH5_MYCGA SDIVRIYDAFSAREDIPGFARVVSHEEIKANEYNLNIPKYIDLVDNGDNH 489

:* * :** : :*.*.: ... : : : :: * . * *

sp|P08957|T1MK_ECOLI GLSPRTEG------------------------------------------ 418

tr|Q7NAH5|Q7NAH5_MYCGA NLYSSIFSGIPHNDIDKLSDFWSTFPTLKKALLNDNGKNYQLKDHDVEKV 539

.* . .

sp|P08957|T1MK_ECOLI --------------------------------------------------

tr|Q7NAH5|Q7NAH5_MYCGA ISNNEEVKKYLSDFNKSLESLRTYFKKSLIDTDLNVIDLYNVYQQFLDKI 589

sp|P08957|T1MK_ECOLI ---------------------EWSFNAEETEVADSEENKN---------- 437

tr|Q7NAH5|Q7NAH5_MYCGA QSVLKSYKLLDYYQAFQLFDNEWTIIENDLKVIKSADNKNSFDTIRELKE 639

**:: :: :* .* :***

sp|P08957|T1MK_ECOLI ---------TDQHLATS--RWRKFSREWIRTAKSDSLDISWLKDKDSIDA 476

tr|Q7NAH5|Q7NAH5_MYCGA HDSSTISAKADKKLVSKNTTYGIYQTPVIPFEFVTKLKFNNQLEALEINS 689

:*::*.:. : :. * .*.:. : .*::

sp|P08957|T1MK_ECOLI DSLPEPDVLAAEAMGELVQALS--------------ELDALMRELG---- 508

tr|Q7NAH5|Q7NAH5_MYCGA NKVEEINARLEELLNEVAGYESDVVNNFYKKEENKLNFDEIKKQLKNLSV 739

:.: * :. * :.*:. * ::* : ::*

sp|P08957|T1MK_ECOLI -ASDEADLQRQLLEEAFGGVKE---------------------------- 529

tr|Q7NAH5|Q7NAH5_MYCGA VAKSQPESVEALLVEALSIDKEKRALNSAIRKAKLQLEKDTIQAYSKLTD 789

*..:.: . ** **:. **

sp|P08957|T1MK_ECOLI --------------------------------------------------

tr|Q7NAH5|Q7NAH5_MYCGA EEAKTLLCLKWIDPLITAISKLTKYKIENLASELNRLDKKYIDKLSDLEV 839

sp|P08957|T1MK_ECOLI ------------------------------------

tr|Q7NAH5|Q7NAH5_MYCGA QIIQTQNSLIELINQLEGPDLDMEGLNELKKILGKK 875

1. **5′- 3′-exonuclease (gene name *exo*)**

>sp|P00582|DPO1_ECOLI DNA polymerase I OS=Escherichia coli (strain K12) GN=polA PE=1 SV=1

MVQIPQNPLILVDGSSYLYRAYHAFPPLTNSAGEPTGAMYGVLNMLRSLIMQYKPTHAAV

VFDAKGKTFRDELFEHYKSHRPPMPDDLRAQIEPLHAMVKAMGLPLLAVSGVEADDVIGT

LAREAEKAGRPVLISTGDKDMAQLVTPNITLINTMTNTILGPEEVVNKYGVPPELIIDFL

ALMGDSSDNIPGVPGVGEKTAQALLQGLGGLDTLYAEPEKIAGLSFRGAKTMAAKLEQNK

EVAYLSYQLATIKTDVELELTCEQLEVQQPAAEELLGLFKKYEFKRWTADVEAGKWLQAK

GAKPAAKPQETSVADEAPEVTATVISYDNYVTILDEETLKAWIAKLEKAPVFAFDTETDS

LDNISANLVGLSFAIEPGVAAYIPVAHDYLDAPDQISRERALELLKPLLEDEKALKVGQN

LKYDRGILANYGIELRGIAFDTMLESYILNSVAGRHDMDSLAERWLKHKTITFEEIAGKG

KNQLTFNQIALEEAGRYAAEDADVTLQLHLKMWPDLQKHKGPLNVFENIEMPLVPVLSRI

ERNGVKIDPKVLHNHSEELTLRLAELEKKAHEIAGEEFNLSSTKQLQTILFEKQGIKPLK

KTPGGAPSTSEEVLEELALDYPLPKVILEYRGLAKLKSTYTDKLPLMINPKTGRVHTSYH

QAVTATGRLSSTDPNLQNIPVRNEEGRRIRQAFIAPEDYVIVSADYSQIELRIMAHLSRD

KGLLTAFAEGKDIHRATAAEVFGLPLETVTSEQRRSAKAINFGLIYGMSAFGLARQLNIP

RKEAQKYMDLYFERYPGVLEYMERTRAQAKEQGYVETLDGRRLYLPDIKSSNGARRAAAE

RAAINAPMQGTAADIIKRAMIAVDAWLQAEQPRVRMIMQVHDELVFEVHKDDVDAVAKQI

HQLMENCTRLDVPLLVEVGSGENWDQAH

>sp|O34996|DPO1_BACSU DNA polymerase I OS=Bacillus subtilis (strain 168) GN=polA PE=3 SV=1

MTERKKLVLVDGNSLAYRAFFALPLLSNDKGVHTNAVYGFAMILMKMLEDEKPTHMLVAF

DAGKTTFRHGTFKEYKGGRQKTPPELSEQMPFIRELLDAYQISRYELEQYEADDIIGTLA

KSAEKDGFEVKVFSGDKDLTQLATDKTTVAITRKGITDVEFYTPEHVKEKYGLTPEQIID

MKGLMGDSSDNIPGVPGVGEKTAIKLLKQFDSVEKLLESIDEVSGKKLKEKLEEFKDQAL

MSKELATIMTDAPIEVSVSGLEYQGFNREQVIAIFKDLGFNTLLERLGEDSAEAEQDQSL

EDINVKTVTDVTSDILVSPSAFVVEQIGDNYHEEPILGFSIVNETGAYFIPKDIAVESEV

FKEWVENDEQKKWVFDSKRAVVALRWQGIELKGAEFDTLLAAYIINPGNSYDDVASVAKD

YGLHIVSSDESVYGKGAKRAVPSEDVLSEHLGRKALAIQSLREKLVQELENNDQLELFEE

LEMPLALILGEMESTGVKVDVDRLKRMGEELGAKLKEYEEKIHEIAGEPFNINSPKQLGV

ILFEKIGLPVVKKTKTGYSTSADVLEKLADKHDIVDYILQYRQIGKLQSTYIEGLLKVTR

PDSHKVHTRFNQALTQTGRLSSTDPNLQNIPIRLEEGRKIRQAFVPSEKDWLIFAADYSQ

IELRVLAHISKDENLIEAFTNDMDIHTKTAMDVFHVAKDEVTSAMRRQAKAVNFGIVYGI

SDYGLSQNLGITRKEAGAFIDRYLESFQGVKAYMEDSVQEAKQKGYVTTLMHRRRYIPEL

TSRNFNIRSFAERTAMNTPIQGSAADIIKKAMIDMAAKLKEKQLKARLLLQVHDELIFEA

PKEEIEILEKLVPEVMEHALALDVPLKVDFASGPSWYDAK

>tr|Q7NBN5|Q7NBN5_MYCGA 5'-3' exonuclease OS=Mycoplasma gallisepticum (strain R(low / passage 15 / clone 2)) GN=exo PE=4 SV=2

MKSTKKALIIDGNSLVFRAFYATLSMYEYAIKKGIRPSNGIKTSLKMINKILNSDQYDYA

LVAFDSKEKTDRAKIYEGYKATRKKPVEGLIEQLVALQDGFSYLGLNVLSSPGIEADDLI

GSFSALANKDQITCHIYTSDQDIFQLVNQYNVVYQFVKGVSVFNQVHERNFQEHFHDLKP

EDVIQYKALVGDSSDNIPGVKGIGEKTAVQLIKDYLNIDNIYANLDQIKPSIKDKLVANK

ANCYLSKELATIRTDCLVDQYINNFKLKPLDQQNYFAFCEYYKISHLD

sp|P00582|DPO1_ECOLI MVQIPQNPLILVDGSSYLYRAYHAFPPLTNSAGEP----TGAMYGVLNML 46

sp|O34996|DPO1_BACSU --MTERKKLVLVDGNSLAYRAFFALPLLSNDKGVH----TNAVYGFAMIL 44

tr|Q7NBN5|Q7NBN5_MYCGA --MKSTKKALIIDGNSLVFRAFYATLSMYEYAIKKGIRPSNGIKTSLKMI 48

: :::**.* :**:.* : : :..: ::

sp|P00582|DPO1_ECOLI RSLIMQYKPTHAAVVFDAKGKTFRDELFEHYKSHRPPMPDDLRAQIEPLH 96

sp|O34996|DPO1_BACSU MKMLEDEKPTHMLVAFDAGKTTFRHGTFKEYKGGRQKTPPELSEQMPFIR 94

tr|Q7NBN5|Q7NBN5_MYCGA NKILNSDQYDYALVAFDSKEKTDRAKIYEGYKATRKKPVEGLIEQLVALQ 98

.:: . : : *.**: .* * :: **. * * *: ::

sp|P00582|DPO1_ECOLI AMVKAMGLPLLAVSGVEADDVIGTLAREAEKAGRPVLISTGDKDMAQLVT 146

sp|O34996|DPO1_BACSU ELLDAYQISRYELEQYEADDIIGTLAKSAEKDGFEVKVFSGDKDLTQLAT 144

tr|Q7NBN5|Q7NBN5_MYCGA DGFSYLGLNVLSSPGIEADDLIGSFSALANKDQITCHIYTSDQDIFQLVN 148

.. : ****:**::: *:* : :.*:*: **..

sp|P00582|DPO1_ECOLI PNITLINTMT----NTILGPEEVVNKYG-VPPELIIDFLALMGDSSDNIP 191

sp|O34996|DPO1_BACSU DKTTVAITRKGITDVEFYTPEHVKEKYG-LTPEQIIDMKGLMGDSSDNIP 193

tr|Q7NBN5|Q7NBN5_MYCGA QYNVVYQFVKGVSVFNQVHERNFQEHFHDLKPEDVIQYKALVGDSSDNIP 198

.: . ... ::: : ** :*: .*:********

sp|P00582|DPO1_ECOLI GVPGVGEKTAQALLQGLGGLDTLYAEPEKIAGLSFRGAKTMAAKLEQNKE 241

sp|O34996|DPO1_BACSU GVPGVGEKTAIKLLKQFDSVEKLLESIDEVS------GKKLKEKLEEFKD 237

tr|Q7NBN5|Q7NBN5_MYCGA GVKGIGEKTAVQLIKDYLNIDNIYANLDQIK-------PSIKDKLVANKA 241

** *:***** *:: .::.: . ::: .: ** *

sp|P00582|DPO1_ECOLI VAYLSYQLATIKTDVELELTCEQLEVQQPAAEELLGLFKKYEFKRWTADV 291

sp|O34996|DPO1_BACSU QALMSKELATIMTDAPIEVSVSGLEYQGFNREQVIAIFKDLGFN------ 281

tr|Q7NBN5|Q7NBN5_MYCGA NCYLSKELATIRTDCLVDQYINNFKLKPLDQQNYFAFCEYYKIS------ 285

. :* :**** ** :: . :: : :: :.: : :.

sp|P00582|DPO1_ECOLI EAGKWLQAKGAKPAAKPQETSVADEAPEVTATVISYDNYVTILDEETLKA 341

sp|O34996|DPO1_BACSU ---TLLERLGEDSAEAEQDQSLEDINVKTVTDVTS--------------- 313

tr|Q7NBN5|Q7NBN5_MYCGA ----HLD------------------------------------------- 288

*:

sp|P00582|DPO1_ECOLI WIAKLEKAPVFAFDTETDSLDNISANLVGLSFAIEPGVAAYIPVAHDYLD 391

sp|O34996|DPO1_BACSU --DILVSPSAFVVEQIGDNYH--EEPILGFSIVNETG--AYF-------- 349

tr|Q7NBN5|Q7NBN5_MYCGA --------------------------------------------------

sp|P00582|DPO1_ECOLI APDQISRERALELLKPLLEDEKALKVGQNLKYDRGILANYGIELRGIAFD 441

sp|O34996|DPO1_BACSU IPKDIAVES--EVFKEWVENDEQKKWVFDSKRAVVALRWQGIELKGAEFD 397

tr|Q7NBN5|Q7NBN5_MYCGA --------------------------------------------------

sp|P00582|DPO1_ECOLI TMLESYILNSVAGRHDMDSLAERWLKHKTITFEEIAGKGKNQLTFNQIAL 491

sp|O34996|DPO1_BACSU TLLAAYIINPGNSYDDVASVAKDYGLHIVSSDESVYGKGAKRAVPSEDVL 447

tr|Q7NBN5|Q7NBN5_MYCGA --------------------------------------------------

sp|P00582|DPO1_ECOLI EEAGRYAAEDADVTLQLHLKMWPDLQKHKGPLNVFENIEMPLVPVLSRIE 541

sp|O34996|DPO1_BACSU SEH---LGRKALAIQSLREKLVQELENND-QLELFEELEMPLALILGEME 493

tr|Q7NBN5|Q7NBN5_MYCGA --------------------------------------------------

sp|P00582|DPO1_ECOLI RNGVKIDPKVLHNHSEELTLRLAELEKKAHEIAGEEFNLSSTKQLQTILF 591

sp|O34996|DPO1_BACSU STGVKVDVDRLKRMGEELGAKLKEYEEKIHEIAGEPFNINSPKQLGVILF 543

tr|Q7NBN5|Q7NBN5_MYCGA --------------------------------------------------

sp|P00582|DPO1_ECOLI EKQGIKPLKKTPGGAPSTSEEVLEELALDYPLPKVILEYRGLAKLKSTYT 641

sp|O34996|DPO1_BACSU EKIGLPVVKKTKTGY-STSADVLEKLADKHDIVDYILQYRQIGKLQSTYI 592

tr|Q7NBN5|Q7NBN5_MYCGA --------------------------------------------------

sp|P00582|DPO1_ECOLI DKLPLMINPKTGRVHTSYHQAVTATGRLSSTDPNLQNIPVRNEEGRRIRQ 691

sp|O34996|DPO1_BACSU EGLLKVTRPDSHKVHTRFNQALTQTGRLSSTDPNLQNIPIRLEEGRKIRQ 642

tr|Q7NBN5|Q7NBN5_MYCGA --------------------------------------------------

sp|P00582|DPO1_ECOLI AFIAP-EDYVIVSADYSQIELRIMAHLSRDKGLLTAFAEGKDIHRATAAE 740

sp|O34996|DPO1_BACSU AFVPSEKDWLIFAADYSQIELRVLAHISKDENLIEAFTNDMDIHTKTAMD 692

tr|Q7NBN5|Q7NBN5_MYCGA --------------------------------------------------

sp|P00582|DPO1_ECOLI VFGLPLETVTSEQRRSAKAINFGLIYGMSAFGLARQLNIPRKEAQKYMDL 790

sp|O34996|DPO1_BACSU VFHVAKDEVTSAMRRQAKAVNFGIVYGISDYGLSQNLGITRKEAGAFIDR 742

tr|Q7NBN5|Q7NBN5_MYCGA --------------------------------------------------

sp|P00582|DPO1_ECOLI YFERYPGVLEYMERTRAQAKEQGYVETLDGRRLYLPDIKSSNGARRAAAE 840

sp|O34996|DPO1_BACSU YLESFQGVKAYMEDSVQEAKQKGYVTTLMHRRRYIPELTSRNFNIRSFAE 792

tr|Q7NBN5|Q7NBN5_MYCGA --------------------------------------------------

sp|P00582|DPO1_ECOLI RAAINAPMQGTAADIIKRAMIAVDAWLQAEQPRVRMIMQVHDELVFEVHK 890

sp|O34996|DPO1_BACSU RTAMNTPIQGSAADIIKKAMIDMAAKLKEKQLKARLLLQVHDELIFEAPK 842

tr|Q7NBN5|Q7NBN5_MYCGA --------------------------------------------------

sp|P00582|DPO1_ECOLI DDVDAVAKQIHQLMENCTRLDVPLLVEVGSGENWDQAH 928

sp|O34996|DPO1_BACSU EEIEILEKLVPEVMEHALALDVPLKVDFASGPSWYDAK 880

tr|Q7NBN5|Q7NBN5_MYCGA --------------------------------------

1. **Putative vsr protein (gene name *MGA_0793*)**

Gene is absent in *B. subtilis* (strain 168) genome.

>sp|P09184|VSR_ECOLI Very short patch repair protein OS=Escherichia coli (strain K12) GN=vsr PE=1 SV=3

MADVHDKATRSKNMRAIATRDTAIEKRLASLLTGQGLAFRVQDASLPGRPDFVVDEYRCV

IFTHGCFWHHHHCYLFKVPATRTEFWLEKIGKNVERDRRDISRLQELGWRVLIVWECALR

GREKLTDEALTERLEEWICGEGASAQIDTQGIHLLA

>tr|Q7NC14|Q7NC14_MYCGA Putative helicase superfamily protein OS=Mycoplasma gallisepticum (strain R(low / passage 15 / clone 2)) GN=MYCGA0950 PE=4 SV=2

MANDWTWLRTRLINNKTKSNTFWIRTRGSSVMDIGVLLKLTSNIYDLSIKGILSYLNSND

TLELDLRKLRELSDLEAFNLFGIETAKELYKEYTDQFSKIFKKLVKEERVTGDTSLFIGL

PIIEGCNQWGDSYRAPLLYVEVVLYPVNQYQKIVFKINRSEFWINTTILAVEASKRGILF

ENKYDSSKLDFEQALEIFRTFDIGFKKPSTNELINFKEMTKKAFLENWEQNGGINNIVNN

VVLGNFDIKGDKLLKDFTEILDKDPDTVDEVFNNKKDLLFNYEKFANEYSLSDIYLTSHL

DFFQQLAVKHALEGDVVIEGPPGTGKSETILNILINIALKGKTALFVSEKTTATEVVYNR

LGKFKHLALYIPSLNKEPGKFYRQFSDYENYFSENYYDQVHKTPNAKFDPDYLKRYLEQS

YIIHKIYNYEINSGENVYSFLNLILNYKPMDVDHINIDDYTRFDEWLKIYTNQDWMTKHQ

EYIALFNEIDTKWKASTFATFLKIHQKDPNDIKTLLYAIHLYAKKGIVKDEYRVSFFFRP

HEKVIESAKLVTEQINKFIELEQYKSETKKRTILKNLEIDIKRYHKQYFNSWFVQNHSGA

FLSKLNSAQSTLDNLTDNYSSDVDVYIQSCKRNLKAEIIKNFYELYRHDKRGLLDVCRQG

RNKSFKNIAWWFKLNREIIKKMFKIHIMSFETASILLENKKDLYDYVIVDEASQVFLERA

IPALYRAKKYIIAGDTKQLQPSSFFSSRSDYDDVALDKLADEEILEVEESVNAVSLIHYL

KERSRINVVLRYHYRSNFGDLIAFTNDHVYDNELIFMNKAIKQKESFIVHDIIDGKWKDR

KNIPEAQAIVSRIQRLTKTADYQKSLGIVAFNRSQADLIELMLDKLNDPLVNEWRERNND

NGEYIGLFVKSVENVQGDERDIIIFSVAYDKSVVSYGPISSTTNGVNRLNVAITRAKDRI

ELFKTNKASEYNGWGSSSAGTRLFVEYLSYCENVANHSDYTTYDRQTTEIEEKLKDKSLI

FDDVKSTLEKAFGQYFTIKRNVDNGSYNFDFVIYHEEVPFLVIDLDIKPFKGMADFNESF

IYRNIFLKNRGWKHFIIWSTEWKLNKRKVLLAIKDILDKRIQMNQK

sp|P09184|VSR_ECOLI 1 ----------------------------------------------------------------------------MADV 4

tr|Q7NC14|Q7NC14_MYCGA 1 MANDWTWLRTRLINNKTKSNTFWIRTRGSSVMDIGVLLKLTSNIYDLSIKGILSYLNSNDTLELDLRKLRELSDLEAFNL 80

sp|P09184|VSR_ECOLI 5 HDKATRSKNMRAIATRDTAIEKRLASLLTGQGLAFRVQDASLPGRPDFVVDEYRCVIFTHGCFWHHHHCY---LFKVPAT 81

tr|Q7NC14|Q7NC14_MYCGA 81 FGIETAKELYKEYTDQFSKIFKKLVKEERVTGDTSLFIGLPIIEGCNQWGDSYRAPLLYVEVVLYPVNQYQKIVFKI--N 158

sp|P09184|VSR_ECOLI 82 RTEFWLEKIGKNVERDRR--------DISRLQ-ELGWRVLIVWECALRG---REKLTDEALTER--LEEWICGEGAS--- 144

tr|Q7NC14|Q7NC14_MYCGA 159 RSEFWINTTILAVEASKRGILFENKYDSSKLDFEQALEIFRTFDIGFKKPSTNELINFKEMTKKAFLENWEQNGGINNIV 238

sp|P09184|VSR_ECOLI 145 -----AQIDTQGIHLLA--------------------------------------------------------------- 156

tr|Q7NC14|Q7NC14_MYCGA 239 NNVVLGNFDIKGDKLLKDFTEILDKDPDTVDEVFNNKKDLLFNYEKFANEYSLSDIYLTSHLDFFQQLAVKHALEGDVVI 318

sp|P09184|VSR_ECOLI --------------------------------------------------------------------------------

tr|Q7NC14|Q7NC14_MYCGA 319 EGPPGTGKSETILNILINIALKGKTALFVSEKTTATEVVYNRLGKFKHLALYIPSLNKEPGKFYRQFSDYENYFSENYYD 398

sp|P09184|VSR_ECOLI --------------------------------------------------------------------------------

tr|Q7NC14|Q7NC14_MYCGA 399 QVHKTPNAKFDPDYLKRYLEQSYIIHKIYNYEINSGENVYSFLNLILNYKPMDVDHINIDDYTRFDEWLKIYTNQDWMTK 478

sp|P09184|VSR_ECOLI --------------------------------------------------------------------------------

tr|Q7NC14|Q7NC14_MYCGA 479 HQEYIALFNEIDTKWKASTFATFLKIHQKDPNDIKTLLYAIHLYAKKGIVKDEYRVSFFFRPHEKVIESAKLVTEQINKF 558

sp|P09184|VSR_ECOLI --------------------------------------------------------------------------------

tr|Q7NC14|Q7NC14_MYCGA 559 IELEQYKSETKKRTILKNLEIDIKRYHKQYFNSWFVQNHSGAFLSKLNSAQSTLDNLTDNYSSDVDVYIQSCKRNLKAEI 638

sp|P09184|VSR_ECOLI --------------------------------------------------------------------------------

tr|Q7NC14|Q7NC14_MYCGA 639 IKNFYELYRHDKRGLLDVCRQGRNKSFKNIAWWFKLNREIIKKMFKIHIMSFETASILLENKKDLYDYVIVDEASQVFLE 718

sp|P09184|VSR_ECOLI --------------------------------------------------------------------------------

tr|Q7NC14|Q7NC14_MYCGA 719 RAIPALYRAKKYIIAGDTKQLQPSSFFSSRSDYDDVALDKLADEEILEVEESVNAVSLIHYLKERSRINVVLRYHYRSNF 798

sp|P09184|VSR_ECOLI --------------------------------------------------------------------------------

tr|Q7NC14|Q7NC14_MYCGA 799 GDLIAFTNDHVYDNELIFMNKAIKQKESFIVHDIIDGKWKDRKNIPEAQAIVSRIQRLTKTADYQKSLGIVAFNRSQADL 878

sp|P09184|VSR_ECOLI --------------------------------------------------------------------------------

tr|Q7NC14|Q7NC14_MYCGA 879 IELMLDKLNDPLVNEWRERNNDNGEYIGLFVKSVENVQGDERDIIIFSVAYDKSVVSYGPISSTTNGVNRLNVAITRAKD 958

sp|P09184|VSR_ECOLI --------------------------------------------------------------------------------

tr|Q7NC14|Q7NC14_MYCGA 959 RIELFKTNKASEYNGWGSSSAGTRLFVEYLSYCENVANHSDYTTYDRQTTEIEEKLKDKSLIFDDVKSTLEKAFGQYFTI 1038

sp|P09184|VSR_ECOLI --------------------------------------------------------------------------------

tr|Q7NC14|Q7NC14_MYCGA 1039 KRNVDNGSYNFDFVIYHEEVPFLVIDLDIKPFKGMADFNESFIYRNIFLKNRGWKHFIIWSTEWKLNKRKVLLAIKDILD 1118

sp|P09184|VSR_ECOLI --------

tr|Q7NC14|Q7NC14_MYCGA 1119 KRIQMNQK 1126

1. **Putative MutH analogue (gene name *MGA_0195*)**

Gene is absent in *B. subtilis* (strain 168) genome.

>sp|P06722|MUTH_ECOLI DNA mismatch repair protein MutH OS=Escherichia coli (strain K12) GN=mutH PE=1 SV=3

MSQPRPLLSPPETEEQLLAQAQQLSGYTLGELAALVGLVTPENLKRDKGWIGVLLEIWLG

ASAGSKPEQDFAALGVELKTIPVDSLGRPLETTFVCVAPLTGNSGVTWETSHVRHKLKRV

LWIPVEGERSIPLAQRRVGSPLLWSPNEEEDRQLREDWEELMDMIVLGQVERITARHGEY

LQIRPKAANAKALTEAIGARGERILTLPRGFYLKKNFTSALLARHFLIQ

>tr|Q7NAY4|Q7NAY4_MYCGA Uncharacterized protein OS=Mycoplasma gallisepticum (strain R(low / passage 15 / clone 2)) GN=MYCGA5010 PE=4 SV=1

MLINYISFIKHGFYIIILINKNMPFKKDLNLSDCFKIVDNQLVLSEKYKQEYGSKFKKIT

GSRFPNVLGINEFNTPFIEWLKMVNLYYETMDPILSKAGVVIEPKVREYIMNKFKINYKT

YDPIKVGFDLFKDNQIFGGIPDGEPVDQDGNLLYDENHPMLEIKTTSIDKLSYKKVDGLL

RMMVDQSGLPIVKAKREKYFEWYDENKQIKVKKEYVLQLSLYLYLRNAKYGRFGVIFLRP

EDYQNPEAIDLSQRLIDVVDMKVEKSSIEPYIEQATQWYQDHIIKGISPKMTRSDLEFLK

LHKII

p|P06722|MUTH_ECOLI -----------------------------------MSQPRPLLSPPETEE 15

tr|Q7NAY4|Q7NAY4_MYCGA MLINYISFIKHGFYIIILINKNMPFKKDLNLSDCFKIVDNQLVLSEKYKQ 50

. *: . : ::

sp|P06722|MUTH_ECOLI QLLAQAQQLSGYTLGELAALVGLVTP--ENLKRDKGWIGVLLEIWLGASA 63

tr|Q7NAY4|Q7NAY4_MYCGA EYGSKFKKITGSRFPNVLGINEFNTPFIEWLKMVNLYYETMDPILSKAGV 100

: :: ::::* : :: .: : ** * ** : : .: * *..

sp|P06722|MUTH_ECOLI GSKP---EQDFAALGVELKTIPVDSLGRPLETTFVCVA------PLTGNS 104

tr|Q7NAY4|Q7NAY4_MYCGA VIEPKVREYIMNKFKINYKTYDPIKVGFDLFKDNQIFGGIPDGEPVDQDG 150

:* * : : :: ** .:* * . .. *: :.

sp|P06722|MUTH_ECOLI GVTWETSHVRHKLKRVLWIPVEGERS---IPLAQRRVGSPLLWSPNE--- 148

tr|Q7NAY4|Q7NAY4_MYCGA NLLYDENHPMLEIKTTSIDKLSYKKVDGLLRMMVDQSGLPIVKAKREKYF 200

.: :: .* ::* . :. :: : : : * *:: : .*

sp|P06722|MUTH_ECOLI ---EEDRQLREDWEELMDMIVLGQVERITARHGEYLQIRPKAAN------ 189

tr|Q7NAY4|Q7NAY4_MYCGA EWYDENKQIKVKKEYVLQLSLYLYLRNAKYGRFGVIFLRPEDYQNPEAID 250

:*::*:: . * :::: : :.. . : : :**: :

sp|P06722|MUTH_ECOLI -AKALTEAIGARGERILTLP--------------RGFYLKKNFTSALLAR 224

tr|Q7NAY4|Q7NAY4_MYCGA LSQRLIDVVDMKVEKSSIEPYIEQATQWYQDHIIKGISPKMTRSDLEFLK 300

:: * :.:. : *: * :*: * . :. : :

sp|P06722|MUTH_ECOLI HFLIQ 229

tr|Q7NAY4|Q7NAY4_MYCGA LHKII 305

. *

1. **Excinuclease ABC subunit A (gene name *uvrA*)**

>sp|P0A698|UVRA_ECOLI UvrABC system protein A OS=Escherichia coli (strain K12) GN=uvrA PE=1 SV=1

MDKIEVRGARTHNLKNINLVIPRDKLIVVTGLSGSGKSSLAFDTLYAEGQRRYVESLSAY

ARQFLSLMEKPDVDHIEGLSPAISIEQKSTSHNPRSTVGTITEIHDYLRLLFARVGEPRC

PDHDVPLAAQTVSQMVDNVLSQPEGKRLMLLAPIIKERKGEHTKTLENLASQGYIRARID

GEVCDLSDPPKLELQKKHTIEVVVDRFKVRDDLTQRLAESFETALELSGGTAVVADMDDP

KAEELLFSANFACPICGYSMRELEPRLFSFNNPAGACPTCDGLGVQQYFDPDRVIQNPEL

SLAGGAIRGWDRRNFYYFQMLKSLADHYKFDVEAPWGSLSANVHKVVLYGSGKENIEFKY

MNDRGDTSIRRHPFEGVLHNMERRYKETESSAVREELAKFISNRPCASCEGTRLRREARH

VYVENTPLPAISDMSIGHAMEFFNNLKLAGQRAKIAEKILKEIGDRLKFLVNVGLNYLTL

SRSAETLSGGEAQRIRLASQIGAGLVGVMYVLDEPSIGLHQRDNERLLGTLIHLRDLGNT

VIVVEHDEDAIRAADHVIDIGPGAGVHGGEVVAEGPLEAIMAVPESLTGQYMSGKRKIEV

PKKRVPANPEKVLKLTGARGNNLKDVTLTLPVGLFTCITGVSGSGKSTLINDTLFPIAQR

QLNGATIAEPAPYRDIQGLEHFDKVIDIDQSPIGRTPRSNPATYTGVFTPVRELFAGVPE

SRARGYTPGRFSFNVRGGRCEACQGDGVIKVEMHFLPDIYVPCDQCKGKRYNRETLEIKY

KGKTIHEVLDMTIEEAREFFDAVPALARKLQTLMDVGLTYIRLGQSATTLSGGEAQRVKL

ARELSKRGTGQTLYILDEPTTGLHFADIQQLLDVLHKLRDQGNTIVVIEHNLDVIKTADW

IVDLGPEGGSGGGEILVSGTPETVAECEASHTARFLKPML

>sp|O34863|UVRA_BACSU UvrABC system protein A OS=Bacillus subtilis (strain 168) GN=uvrA PE=3 SV=1

MAMDRIEVKGARAHNLKNIDVTIPRDQLVVVTGLSGSGKSSLAFDTIYAEGQRRYVESLS

AYARQFLGQMDKPDVDAIEGLSPAISIDQKTTSRNPRSTVGTVTEIYDYLRLLYARVGKP

HCPEHGIEITSQTIEQMVDRILEYPERTKLQVLAPIVSGRKGAHVKVLEQIRKQGYVRVR

IDGEMAELSDDIELEKNKKHSIEVVIDRIVVKEGVAARLSDSLETALRLGEGRVMIDVIG

EEELMFSEHHACPHCGFSIGELEPRLFSFNSPFGACPTCDGLGMKLEVDADLVIPNQDLS

LKENAVAPWTPISSQYYPQLLEAVCTHYGIDMDVPVKDLPKHQLDKVLYGSGDDLIYFRY

ENDFGQIREGEIQFEGVLRNIERRYKETGSDFIREQMEQYMSQKSCPTCKGYRLKKEALA

VLIDGRHIGKITELSVADALAFFKDLTLSEKDMQIANLILREIVERLSFLDKVGLDYLTL

SRAAGTLSGGEAQRIRLATQIGSRLSGVLYILDEPSIGLHQRDNDRLISALKNMRDLGNT

LIVVEHDEDTMMAADYLIDIGPGAGIHGGQVISAGTPEEVMEDPNSLTGSYLSGKKFIPL

PPERRKPDGRYIEIKGASENNLKKVNAKFPLGTFTAVTGVSGSGKSTLVNEILHKALAQK

LHKAKAKPGSHKEIKGLDHLDKVIDIDQAPIGRTPRSNPATYTGVFDDIRDVFAQTNEAK

VRGYKKGRFSFNVKGGRCEACRGDGIIKIEMHFLPDVYVPCEVCHGKRYNRETLEVTYKG

KSISDVLDMTVEDALSFFENIPKIKRKLQTLYDVGLGYITLGQPATTLSGGEAQRVKLAS

ELHKRSTGRTLYILDEPTTGLHVDDIARLLVVLQRLVDNGDTVLVIEHNLDIIKTADYIV

DLGPEGGAGGGTIVASGTPEEITEVEESYTGRYLKPVIERDKTRMKSLLKAKETATS

>tr|Q7NC22|Q7NC22_MYCGA UvrABC system protein A OS=Mycoplasma gallisepticum (strain R(low / passage 15 / clone 2)) GN=uvrA PE=3 SV=2

MKKIKKDSANYISIVGARQNNLKNISLDIPKNQLVVITGLSGSGKSSLAFKTIYAEGQRR

YLESLSPYARQFLGNNDKPDVDSIEGLSPSISIDQKSTSHNPRSTVGTVTEVYDFLRLLW

SRVGDAYCINGHGMIKTTTIKQIIDHVLELEDDSKLQILAPVIKLQKGTFKNEFEKFYKQ

GFMRVLVDGVVYSLDDKIELDKNQKHDISIVIDRLILNKDNQTKLRITDAIETALTVSNG

LIQIISNDQAKYEFSLNHSCDQCGFFIPELEPRLFSFNSPIGACDYCKGLGFTYEPDVDK

IIPNKDLTINEGAIDYFKNRINTSSQDWQRFYSIIRHYQIDLNTPIKNLSKKEINYLLEG

SDEPIEIVIESANRNGISSRLDYVEGIAKLIQRRHLETKSSAARDNYSKYTSEQKCKTCD

GKKLSPAALSVKIGGLDIIEFTNLNVNKALDFILGLEFNEEKTKIAKFVLKEILDRLYFL

VNVGLEYLTLSRNASTLSGGESQRIRLATQIGSRLSGVLYVLDEPSIGLHQKDNDKLIKT

LLSMRDLGNSLIVVEHDEETMMSADYLIDIGPGAGTYGGKVVAAGTVEEVMKNPASLTGQ

YLSKKLEIEQPKKLHPGNGQKIVLKGASANNLKNINVEFPLNKLVVVTGVSGSGKSTLIN

QTLVNGIEKALFNKHVEVGKYKSLIGINNIDKVIKVSQDPIGRTPRSNPATYVSVFDDIR

EVFANVFEAKARGYTKSRFSFNVSGGRCDDCQGDGVKCIEMHFLPDVYVKCSSCNGKKYN

EATLEIKYKNKSIYDVLEMSCEEALEFFKVIPAINRKLQLMCDVGLGYMKLSTNATELSG

GEAQRIKLAKYLQRKATGNTIYVLDEPTTGLHAHDIKKLLSVLNRLVDNGDSVIVIEHNL

ELIKVADHIIDLGPNGGDDGGYLICAGTPQELVKNYTDSSYTARYLAKIMKS

sp|P0A698|UVRA_ECOLI --------MDKIEVRGARTHNLKNINLVIPRDKLIVVTGLSGSGKSSLAF 42

sp|O34863|UVRA_BACSU ------MAMDRIEVKGARAHNLKNIDVTIPRDQLVVVTGLSGSGKSSLAF 44

tr|Q7NC22|Q7NC22_MYCGA MKKIKKDSANYISIVGARQNNLKNISLDIPKNQLVVITGLSGSGKSSLAF 50

: *.: *** :*****.: **:::*:*:*************

sp|P0A698|UVRA_ECOLI DTLYAEGQRRYVESLSAYARQFLSLMEKPDVDHIEGLSPAISIEQKSTSH 92

sp|O34863|UVRA_BACSU DTIYAEGQRRYVESLSAYARQFLGQMDKPDVDAIEGLSPAISIDQKTTSR 94

tr|Q7NC22|Q7NC22_MYCGA KTIYAEGQRRYLESLSPYARQFLGNNDKPDVDSIEGLSPSISIDQKSTSH 100

.*:********:****.******. :***** ******:***:**:**:

sp|P0A698|UVRA_ECOLI NPRSTVGTITEIHDYLRLLFARVGEPRCPDHDVPLAAQTVSQMVDNVLSQ 142

sp|O34863|UVRA_BACSU NPRSTVGTVTEIYDYLRLLYARVGKPHCPEHGIEITSQTIEQMVDRILEY 144

tr|Q7NC22|Q7NC22_MYCGA NPRSTVGTVTEVYDFLRLLWSRVGDAYCINGHGMIKTTTIKQIIDHVLEL 150

********:**::*:****::***.. * : : : *:.*::*.:*.

sp|P0A698|UVRA_ECOLI PEGKRLMLLAPIIKERKGEHTKTLENLASQGYIRARIDGEVCDLSDPPKL 192

sp|O34863|UVRA_BACSU PERTKLQVLAPIVSGRKGAHVKVLEQIRKQGYVRVRIDGEMAELSDDIEL 194

tr|Q7NC22|Q7NC22_MYCGA EDDSKLQILAPVIKLQKGTFKNEFEKFYKQGFMRVLVDGVVYSLDDKIEL 200

: .:* :***::. :** . : :*:: .**::*. :** : .*.* :*

sp|P0A698|UVRA_ECOLI ELQKKHTIEVVVDRFKVRDD--LTQRLAESFETALELSGGTAVVADMDDP 240

sp|O34863|UVRA_BACSU EKNKKHSIEVVIDRIVVKEG--VAARLSDSLETALRLGEGRVMIDVIGE- 241

tr|Q7NC22|Q7NC22_MYCGA DKNQKHDISIVIDRLILNKDNQTKLRITDAIETALTVSNGLIQIISNDQ- 249

: ::** *.:*:**: :... *:::::**** :. * : .:

sp|P0A698|UVRA_ECOLI KAEELLFSANFACPICGYSMRELEPRLFSFNNPAGACPTCDGLGVQQYFD 290

sp|O34863|UVRA_BACSU --EELMFSEHHACPHCGFSIGELEPRLFSFNSPFGACPTCDGLGMKLEVD 289

tr|Q7NC22|Q7NC22_MYCGA --AKYEFSLNHSCDQCGFFIPELEPRLFSFNSPIGACDYCKGLGFTYEPD 297

: ** :.:* **: : **********.* *** *.***. *

sp|P0A698|UVRA_ECOLI PDRVIQNPELSLAGGAIRGWD-RRNFYY--FQMLKSLADHYKFDVEAPWG 337

sp|O34863|UVRA_BACSU ADLVIPNQDLSLKENAVAPWTPISSQYY--PQLLEAVCTHYGIDMDVPVK 337

tr|Q7NC22|Q7NC22_MYCGA VDKIIPNKDLTINEGAIDYFKNRINTSSQDWQRFYSIIRHYQIDLNTPIK 347

* :* * :*:: .*: : . * : :: ** :*::.*

sp|P0A698|UVRA_ECOLI SLSANVHKVVLYGSGKENIEFKYMNDRGDTSIRRHPFEGVLHNMERRYKE 387

sp|O34863|UVRA_BACSU DLPKHQLDKVLYGSGDDLIYFRYENDFGQIREGEIQFEGVLRNIERRYKE 387

tr|Q7NC22|Q7NC22_MYCGA NLSKKEINYLLEGSDEPIEIVIESANRNGISSRLDYVEGIAKLIQRRHLE 397

.*. : . :* **.. . : . .**: : ::**: *

sp|P0A698|UVRA_ECOLI TESSAVREELAKFISNRPCASCEGTRLRREARHVYVENTPLPAISDMSIG 437

sp|O34863|UVRA_BACSU TGSDFIREQMEQYMSQKSCPTCKGYRLKKEALAVLIDGRHIGKITELSVA 437

tr|Q7NC22|Q7NC22_MYCGA TKSSAARDNYSKYTSEQKCKTCDGKKLSPAALSVKIGGLDIIEFTNLNVN 447

* *. *:: :: *:: * :*.* :* * * : . : ::::.:

sp|P0A698|UVRA_ECOLI HAMEFFNNLKLAGQRAKIAEKILKEIGDRLKFLVNVGLNYLTLSRSAETL 487

sp|O34863|UVRA_BACSU DALAFFKDLTLSEKDMQIANLILREIVERLSFLDKVGLDYLTLSRAAGTL 487

tr|Q7NC22|Q7NC22_MYCGA KALDFILGLEFNEEKTKIAKFVLKEILDRLYFLVNVGLEYLTLSRNASTL 497

.*: *: .* : : :**: :*:** :** ** :***:****** * **

sp|P0A698|UVRA_ECOLI SGGEAQRIRLASQIGAGLVGVMYVLDEPSIGLHQRDNERLLGTLIHLRDL 537

sp|O34863|UVRA_BACSU SGGEAQRIRLATQIGSRLSGVLYILDEPSIGLHQRDNDRLISALKNMRDL 537

tr|Q7NC22|Q7NC22_MYCGA SGGESQRIRLATQIGSRLSGVLYVLDEPSIGLHQKDNDKLIKTLLSMRDL 547

****:******:***: * **:*:**********:**::*: :* :***

sp|P0A698|UVRA_ECOLI GNTVIVVEHDEDAIRAADHVIDIGPGAGVHGGEVVAEGPLEAIMAVPESL 587

sp|O34863|UVRA_BACSU GNTLIVVEHDEDTMMAADYLIDIGPGAGIHGGQVISAGTPEEVMEDPNSL 587

tr|Q7NC22|Q7NC22_MYCGA GNSLIVVEHDEETMMSADYLIDIGPGAGTYGGKVVAAGTVEEVMKNPASL 597

**::*******::: :**::******** :**:*:: *. * :* * **

sp|P0A698|UVRA_ECOLI TGQYMSGKRKIEVPKKRVPANPEKVLKLTGARGNNLKDVTLTLPVGLFTC 637

sp|O34863|UVRA_BACSU TGSYLSGKKFIPLPPERRKPD-GRYIEIKGASENNLKKVNAKFPLGTFTA 636

tr|Q7NC22|Q7NC22_MYCGA TGQYLSKKLEIEQPKKLHPGN-GQKIVLKGASANNLKNINVEFPLNKLVV 646

**.*:* * * * : : : : :.** ****.:. :*:. :.

sp|P0A698|UVRA_ECOLI ITGVSGSGKSTLINDTLFPIAQRQLNGATIAEPAPYRDIQGLEHFDKVID 687

sp|O34863|UVRA_BACSU VTGVSGSGKSTLVNEILHKALAQKLH-KAKAKPGSHKEIKGLDHLDKVID 685

tr|Q7NC22|Q7NC22_MYCGA VTGVSGSGKSTLINQTLVNGIEKALF-NKHVEVGKYKSLIGINNIDKVIK 695

:***********:*: * : * .: . ::.: *::::****.

sp|P0A698|UVRA_ECOLI IDQSPIGRTPRSNPATYTGVFTPVRELFAGVPESRARGYTPGRFSFNVRG 737

sp|O34863|UVRA_BACSU IDQAPIGRTPRSNPATYTGVFDDIRDVFAQTNEAKVRGYKKGRFSFNVKG 735

tr|Q7NC22|Q7NC22_MYCGA VSQDPIGRTPRSNPATYVSVFDDIREVFANVFEAKARGYTKSRFSFNVSG 745

:.* *************..** :*::** . *::.***. .****** *

sp|P0A698|UVRA_ECOLI GRCEACQGDGVIKVEMHFLPDIYVPCDQCKGKRYNRETLEIKYKGKTIHE 787

sp|O34863|UVRA_BACSU GRCEACRGDGIIKIEMHFLPDVYVPCEVCHGKRYNRETLEVTYKGKSISD 785

tr|Q7NC22|Q7NC22_MYCGA GRCDDCQGDGVKCIEMHFLPDVYVKCSSCNGKKYNEATLEIKYKNKSIYD 795

***: *:***: :*******:** *. *:**:**. ***:.**.*:* :

sp|P0A698|UVRA_ECOLI VLDMTIEEAREFFDAVPALARKLQTLMDVGLTYIRLGQSATTLSGGEAQR 837

sp|O34863|UVRA_BACSU VLDMTVEDALSFFENIPKIKRKLQTLYDVGLGYITLGQPATTLSGGEAQR 835

tr|Q7NC22|Q7NC22_MYCGA VLEMSCEEALEFFKVIPAINRKLQLMCDVGLGYMKLSTNATELSGGEAQR 845

**:*: *:* .**. :* : **** : **** *: *. ** ********

sp|P0A698|UVRA_ECOLI VKLARELSKRGTGQTLYILDEPTTGLHFADIQQLLDVLHKLRDQGNTIVV 887

sp|O34863|UVRA_BACSU VKLASELHKRSTGRTLYILDEPTTGLHVDDIARLLVVLQRLVDNGDTVLV 885

tr|Q7NC22|Q7NC22_MYCGA IKLAKYLQRKATGNTIYVLDEPTTGLHAHDIKKLLSVLNRLVDNGDSVIV 895

:*** * ::.**.*:*:********* ** :** **::* *:*::::*

sp|P0A698|UVRA_ECOLI IEHNLDVIKTADWIVDLGPEGGSGGGEILVSGTPETVAE--CEASHTARF 935

sp|O34863|UVRA_BACSU IEHNLDIIKTADYIVDLGPEGGAGGGTIVASGTPEEITE--VEESYTGRY 933

tr|Q7NC22|Q7NC22_MYCGA IEHNLELIKVADHIIDLGPNGGDDGGYLICAGTPQELVKNYTDSSYTARY 945

*****::**.** *:****:** .** :: :***: :.: : *:*.*:

sp|P0A698|UVRA_ECOLI LKPML------------------- 940

sp|O34863|UVRA_BACSU LKPVIERDKTRMKSLLKAKETATS 957

tr|Q7NC22|Q7NC22_MYCGA LAKIMKS----------------- 952

* ::

| **Organism** | **ATP hydrolysis*** |
| --- | --- |
| E. coli (strain K12) | K37, K646 |
| B. subtilis (strain 168) | No information |
| M. gallisepticum (strain R(low / passage 15 / clone 2)) | No information |

*Site-specific mutagenesis of conserved residues within Walker A and B sequences of Escherichia coli UvrA protein. (<http://pubs.acs.org/doi/pdf/10.1021/bi00230a004>)

1. **Excinuclease ABC subunit B (gene name *uvrB*)**

>sp|P0A8F8|UVRB_ECOLI UvrABC system protein B OS=Escherichia coli (strain K12) GN=uvrB PE=1 SV=2

MSKPFKLNSAFKPSGDQPEAIRRLEEGLEDGLAHQTLLGVTGSGKTFTIANVIADLQRPT

MVLAPNKTLAAQLYGEMKEFFPENAVEYFVSYYDYYQPEAYVPSSDTFIEKDASVNEHIE

QMRLSATKAMLERRDVVVVASVSAIYGLGDPDLYLKMMLHLTVGMIIDQRAILRRLAELQ

YARNDQAFQRGTFRVRGEVIDIFPAESDDIALRVELFDEEVERLSLFDPLTGQIVSTIPR

FTIYPKTHYVTPRERIVQAMEEIKEELAARRKVLLENNKLLEEQRLTQRTQFDLEMMNEL

GYCSGIENYSRFLSGRGPGEPPPTLFDYLPADGLLVVDESHVTIPQIGGMYRGDRARKET

LVEYGFRLPSALDNRPLKFEEFEALAPQTIYVSATPGNYELEKSGGDVVDQVVRPTGLLD

PIIEVRPVATQVDDLLSEIRQRAAINERVLVTTLTKRMAEDLTEYLEEHGERVRYLHSDI

DTVERMEIIRDLRLGEFDVLVGINLLREGLDMPEVSLVAILDADKEGFLRSERSLIQTIG

RAARNVNGKAILYGDKITPSMAKAIGETERRREKQQKYNEEHGITPQGLNKKVVDILALG

QNIAKTKAKGRGKSRPIVEPDNVPMDMSPKALQQKIHELEGLMMQHAQNLEFEEAAQIRD

QLHQLRELFIAAS

>sp|P37954|UVRB_BACSU UvrABC system protein B OS=Bacillus subtilis (strain 168) GN=uvrB PE=1 SV=2

MKDRFELVSKYQPQGDQPKAIEKLVKGIQEGKKHQTLLGATGTGKTFTVSNLIKEVNKPT

LVIAHNKTLAGQLYSEFKEFFPNNAVEYFVSYYDYYQPEAYVPQTDTFIEKDASINDEID

KLRHSATSALFERRDVIIIASVSCIYGLGSPEEYREMVVSLRTEMEIERNELLRKLVDIQ

YARNDIDFQRGTFRVRGDVVEIFPASRDEHCVRVEFFGDEIERIREVDALTGEILGDRDH

VAIFPASHFVTRAEKMEKAIQNIEKELEEQLKVMHENGKLLEAQRLEQRTRYDLEMMREM

GFCSGIENYSRHLTLRPPGSTPYTLLDYFPDDFMIVVDESHVTIPQVRGMFNGDQARKQV

LVDHGFRLPSALDNRPLRFEEFEKHMHNIVYVSATPGPYEIEHTDEMVEQIIRPTGLLDP

LIDVRPIEGQIDDLIGEIQARIERNERVLVTTLTKKMSEDLTDYLKEIGIKVNYLHSEIK

TLERIEIIRDLRLGKYDVLVGINLLREGLDIPEVSLVAILDADKEGFLRSERSLIQTIGR

AARNAEGRVIMYADKITKSMEIAINETKRRREQQERFNEEHGITPKTINKEIRDVIRATV

AAEDKAEYKTKAAPKLSKMTKKERQKVVEQMEHEMKEAAKALDFERAAELRDLLLELKAE

G

>tr|Q7NC43|Q7NC43_MYCGA UvrABC system protein B OS=Mycoplasma gallisepticum (strain R(low / passage 15 / clone 2)) GN=uvrB PE=3 SV=2

MAEKKFKLVSKNKPAGHQPEAIKKLVDGINKNKKYQTLLGATGTGKTFTIANVIEKTQKK

TLILAHNKTLAAQLYLEFKELFPNNAVEYFVSYFDFYQPEAYIPRTDMYIEKSSVTNDEI

EMLRLASLNSLSTRNDVIVVASVACIYPAANPEDFDIYRIILKVGNTLKLSDLKENLIRL

NYARSPECNEPGTFRIKGDVVDIFPGYVSDHIIRLSFFGDELEEIRKIHPTDSSVIEKYT

SYVLGPANEYILNFERKDTAIKRIQEELMFRVQEFKNQQKLVEAQRLQQRTEYDIDAIKE

FGFCNGIENYAFHLELREKGSTPWTLFDFFGDDWLMVIDESHISVPQVKGMFNTDKSRKT

TLVEYGFRLPSALENRPLNYDEFSNKSDQVIFVSATPNDEEIKLSNNEIIEQIVRPTGLL

DPTVEIRPRLDQINDLMNELKKQKDKNERTFITVTTIKMAEDLTEYLKERNFKCAYIHNE

LKTLERSLILNDLRRGKYDCVVGINLLREGLDIPEVSLVCIFDADKPGYFRSDKALIQTI

GRAARNQNGRVIMYADEMTKAMKIAVDETNRRRKIQEKFNKDHKITPKTIIKPIYDDLKN

KASHKQIEEVMRKTKAKGDKFIKMIEDLRNEMLEAAKNQNYEHAASLRDLIIELETQQLS

KTNK

sp|P0A8F8|UVRB_ECOLI -MSKPFKLNSAFKPSGDQPEAIRRLEEGLEDGLAHQTLLGVTGSGKTFTI 49

sp|P37954|UVRB_BACSU -MKDRFELVSKYQPQGDQPKAIEKLVKGIQEGKKHQTLLGATGTGKTFTV 49

tr|Q7NC43|Q7NC43_MYCGA MAEKKFKLVSKNKPAGHQPEAIKKLVDGINKNKKYQTLLGATGTGKTFTI 50

.. *:* * :* *.**:**.:* .*::.. :*****.**:*****:

sp|P0A8F8|UVRB_ECOLI ANVIADLQRPTMVLAPNKTLAAQLYGEMKEFFPENAVEYFVSYYDYYQPE 99

sp|P37954|UVRB_BACSU SNLIKEVNKPTLVIAHNKTLAGQLYSEFKEFFPNNAVEYFVSYYDYYQPE 99

tr|Q7NC43|Q7NC43_MYCGA ANVIEKTQKKTLILAHNKTLAAQLYLEFKELFPNNAVEYFVSYFDFYQPE 100

:*:* . :: *:::* *****.*** *:**:**:*********:*:****

sp|P0A8F8|UVRB_ECOLI AYVPSSDTFIEKDASVNEHIEQMRLSATKAMLERRDVVVVASVSAIYGLG 149

sp|P37954|UVRB_BACSU AYVPQTDTFIEKDASINDEIDKLRHSATSALFERRDVIIIASVSCIYGLG 149

tr|Q7NC43|Q7NC43_MYCGA AYIPRTDMYIEKSSVTNDEIEMLRLASLNSLSTRNDVIVVASVACIYPAA 150

**:* :* :***.: *:.*: :* :: .:: *.**:::***:.** .

sp|P0A8F8|UVRB_ECOLI DPDLYLKMMLHLTVGMIIDQRAILRRLAELQYARNDQAFQRGTFRVRGEV 199

sp|P37954|UVRB_BACSU SPEEYREMVVSLRTEMEIERNELLRKLVDIQYARNDIDFQRGTFRVRGDV 199

tr|Q7NC43|Q7NC43_MYCGA NPEDFDIYRIILKVGNTLKLSDLKENLIRLNYARSPECNEPGTFRIKGDV 200

.*: : : * . :. : ..* ::***. : ****::*:*

sp|P0A8F8|UVRB_ECOLI IDIFPAESDDIALRVELFDEEVERLSLFDPLTGQIVSTIPRFTIYPKTHY 249

sp|P37954|UVRB_BACSU VEIFPASRDEHCVRVEFFGDEIERIREVDALTGEILGDRDHVAIFPASHF 249

tr|Q7NC43|Q7NC43_MYCGA VDIFPGYVSDHIIRLSFFGDELEEIRKIHPTDSSVIEKYTSYVLGPANEY 250

::***. .: :*:.:*.:*:*.: ... ..:: .: * ..:

sp|P0A8F8|UVRB_ECOLI VTPRERIVQAMEEIKEELAARRKVLLENNKLLEEQRLTQRTQFDLEMMNE 299

sp|P37954|UVRB_BACSU VTRAEKMEKAIQNIEKELEEQLKVMHENGKLLEAQRLEQRTRYDLEMMRE 299

tr|Q7NC43|Q7NC43_MYCGA ILNFERKDTAIKRIQEELMFRVQEFKNQQKLVEAQRLQQRTEYDIDAIKE 300

: *: *::.*::** : : : :: **:* *** ***.:*:: :.*

sp|P0A8F8|UVRB_ECOLI LGYCSGIENYSRFLSGRGPGEPPPTLFDYLPADGLLVVDESHVTIPQIGG 349

sp|P37954|UVRB_BACSU MGFCSGIENYSRHLTLRPPGSTPYTLLDYFPDDFMIVVDESHVTIPQVRG 349

tr|Q7NC43|Q7NC43_MYCGA FGFCNGIENYAFHLELREKGSTPWTLFDFFGDDWLMVIDESHISVPQVKG 350

:*:*.*****: .* * *..* **:*:: * ::*:****:::**: *

sp|P0A8F8|UVRB_ECOLI MYRGDRARKETLVEYGFRLPSALDNRPLKFEEFEALAPQTIYVSATPGNY 399

sp|P37954|UVRB_BACSU MFNGDQARKQVLVDHGFRLPSALDNRPLRFEEFEKHMHNIVYVSATPGPY 399

tr|Q7NC43|Q7NC43_MYCGA MFNTDKSRKTTLVEYGFRLPSALENRPLNYDEFSNKSDQVIFVSATPNDE 400

*:. *::** .**::********:****.::**. : ::*****.

sp|P0A8F8|UVRB_ECOLI ELEKSGGDVVDQVVRPTGLLDPIIEVRPVATQVDDLLSEIRQRAAINERV 449

sp|P37954|UVRB_BACSU EIEHT-DEMVEQIIRPTGLLDPLIDVRPIEGQIDDLIGEIQARIERNERV 448

tr|Q7NC43|Q7NC43_MYCGA EIKLSNNEIIEQIVRPTGLLDPTVEIRPRLDQINDLMNELKKQKDKNERT 450

*:: : .::::*::******** :::** *::**:.*:: : ***.

sp|P0A8F8|UVRB_ECOLI LVTTLTKRMAEDLTEYLEEHGERVRYLHSDIDTVERMEIIRDLRLGEFDV 499

sp|P37954|UVRB_BACSU LVTTLTKKMSEDLTDYLKEIGIKVNYLHSEIKTLERIEIIRDLRLGKYDV 498

tr|Q7NC43|Q7NC43_MYCGA FITVTTIKMAEDLTEYLKERNFKCAYIHNELKTLERSLILNDLRRGKYDC 500

::*. * :*:****:**:* . : *:*.::.*:** *:.*** *::*

sp|P0A8F8|UVRB_ECOLI LVGINLLREGLDMPEVSLVAILDADKEGFLRSERSLIQTIGRAARNVNGK 549

sp|P37954|UVRB_BACSU LVGINLLREGLDIPEVSLVAILDADKEGFLRSERSLIQTIGRAARNAEGR 548

tr|Q7NC43|Q7NC43_MYCGA VVGINLLREGLDIPEVSLVCIFDADKPGYFRSDKALIQTIGRAARNQNGR 550

:***********:******.*:**** *::**:::*********** :*:

sp|P0A8F8|UVRB_ECOLI AILYGDKITPSMAKAIGETERRREKQQKYNEEHGITPQGLNKKVVDILAL 599

sp|P37954|UVRB_BACSU VIMYADKITKSMEIAINETKRRREQQERFNEEHGITPKTINKEIRDVIRA 598

tr|Q7NC43|Q7NC43_MYCGA VIMYADEMTKAMKIAVDETNRRRKIQEKFNKDHKITPKTIIKPIYDDLKN 600

.*:*.*::* :* *:.**:***: *:::*::* ***: : * : * :

sp|P0A8F8|UVRB_ECOLI GQNIAKTKAKGRGKSRPIVEPDNVPMDMSPKALQQKIHELEGLMMQHAQN 649

sp|P37954|UVRB_BACSU -TVAAEDKAEYKTKAAPKLS------KMTKKERQKVVEQMEHEMKEAAKA 641

tr|Q7NC43|Q7NC43_MYCGA -KASHKQIEEVMRKTKAKGD-----------KFIKMIEDLRNEMLEAAKN 638

: : *: . . : :.::. * : *:

sp|P0A8F8|UVRB_ECOLI LEFEEAAQIRDQLHQLRELFIAAS-- 673

sp|P37954|UVRB_BACSU LDFERAAELRDLLLELKAEG------ 661

tr|Q7NC43|Q7NC43_MYCGA QNYEHAASLRDLIIELETQQLSKTNK 664

::*.**.:** : :*.

| **Organism** | **Nucleotide binding (ATP) - potential** | **Motif (Beta-hairpin)** | **Domain (Helicase ATP-binding, Helicase C-terminal, UVR)** |
| --- | --- | --- | --- |
| E. coli (strain K12) | 39-46 | 92-115 | 26-415, 431-597, 633-668 |
| B. subtilis (strain 168) | 39-46 | 92-115 | 26-413, 430-596, 625-660 |
| M. gallisepticum (strain R(low / passage 15 / clone 2)) | 40-47 | 93-116 | 27-184, 432-594, 622-657 |

1. **Excinuclease ABC subunit С (gene name *uvrC*)**

>sp|P0A8G0|UVRC_ECOLI UvrABC system protein C OS=Escherichia coli (strain K12) GN=uvrC PE=1 SV=1

MSDQFDAKAFLKTVTSQPGVYRMYDAGGTVIYVGKAKDLKKRLSSYFRSNLASRKTEALV

AQIQQIDVTVTHTETEALLLEHNYIKLYQPRYNVLLRDDKSYPFIFLSGDTHPRLAMHRG

AKHAKGEYFGPFPNGYAVRETLALLQKIFPIRQCENSVYRNRSRPCLQYQIGRCLGPCVE

GLVSEEEYAQQVEYVRLFLSGKDDQVLTQLISRMETASQNLEFEEAARIRDQIQAVRRVT

EKQFVSNTGDDLDVIGVAFDAGMACVHVLFIRQGKVLGSRSYFPKVPGGTELSEVVETFV

GQFYLQGSQMRTLPGEILLDFNLSDKTLLADSLSELAGRKINVQTKPRGDRARYLKLART

NAATALTSKLSQQSTVHQRLTALASVLKLPEVKRMECFDISHTMGEQTVASCVVFDANGP

LRAEYRRYNITGITPGDDYAAMNQVLRRRYGKAIDDSKIPDVILIDGGKGQLAQAKNVFA

ELDVSWDKNHPLLLGVAKGADRKAGLETLFFEPEGEGFSLPPDSPALHVIQHIRDESHDH

AIGGHRKKRAKVKNTSSLETIEGVGPKRRQMLLKYMGGLQGLRNASVEEIAKVPGISQGL

AEKIFWSLKH

>sp|P14951|UVRC_BACSU UvrABC system protein C OS=Bacillus subtilis (strain 168) GN=uvrC PE=3 SV=2

MNKQLKEKLALLPDQPGCYLMKDRQQTVIYVGKAKVLKNRVRSYFTGSHDAKTQRLVTEI

EDFEYIVTSSNLEALILEMNLIKKHDPKYNVMLKDDKTYPFIKLTHERHPRLIVTRNVKK

DKGRYFGPYPNVQAARETKKLLDRLYPLRKCSKLPDRVCLYYHLGQCLAPCVKDISEETN

RELVESITRFLRGGYNEVKKELEEKMHEAAENLEFERAKELRDQIAHIESTMEKQKMTMN

DLVDRDVFAYAYDKGWMCVQVFFIRQGKLIERDVSMFPLYQEADEEFLTFIGQFYSKNNH

FLPKEILVPDSIDQSMIEQLLETNVHQPKKGPKKELLMLAHKNAKIALKEKFSLIERDEE

RSIGAVQKLGEALNIYTPHRIEAFDNSNIQGTNPVSAMIVFIDGKPYKKEYRKYKIKTVT

GPDDYGSMREVVRRRYTRVLRENLPLPDLIIIDGGKGQINAARDVIENELGLDIPIAGLA

KDEKHRTSNLLIGDPLEVAYLERNSQEFYLLQRIQDEVHRFAISFHRQIRGKSAFQSVLD

DIPGIGEKRKKMLLKHFGSVKKMKEASLEDIKKAGVPAAAAQLLYDKLQK

>sp|Q7NBC4|UVRC_MYCGA UvrABC system protein C OS=Mycoplasma gallisepticum (strain R(low / passage 15 / clone 2)) GN=uvrC PE=3 SV=2

MNFNLKQKLDLAPKKPGCYLWKNHLNEIIYIGKAKNIYKRVHQYFNGPKDLKTSKLVNEI

FYVEFIEVNNENEALLLEANLIKKHKPRYNILLKDNNGYPYILMTKEKYPRLIYTRNFDP

KKGKHYGPFASSEMKAYDLYNLLLKLFPLKNCFNKKGRKCEFYDLNLCMKACTHEVSEAD

YEVMKKKIDYFFHNGADQVLKDLKEKESIASEKFDFEQAKKYLDLQKAINLIFDKQIINL

YSAKERIDVLAYQIKENVICIVLFSYVSSQLVSKNTICDFYYGEEQEVITSYLSQYYKDN

IKPKILYASLDQANATLLKDSLGIEIINPTSGKMNEIMSLALQNVTNELSQKYDSLVKKE

QRINLALDQLKKLIKVDKLNHLEVYDNSNLFNTDKVSAMIVFENNQFNKKKYRKYKIKDQ

QALGDYHYMYEVIYRRLYQALKNNFVDLPDLIILDGGKHQVLAAKKAIVDLQIDKKINLI

GLAKNNKHQTDKIVTFDLDEISLDKSSALYFFLANLQEEVHKFAISFFRKTKAKSLYDSI

LDQIKGLGKKRKQQLIEHFKTIDEIKKASIASLSQVLPIEIAKKLKQKLDQS

sp|P0A8G0|UVRC_ECOLI MSDQFDAKAFLKTVTSQPGVYRMYDAGGTVIYVGKAKDLKKRLSSYFRSNLASRKTEALV 60

sp|P14951|UVRC_BACSU MNKQLKEK--LALLPDQPGCYLMKDRQQTVIYVGKAKVLKNRVRSYFTG-SHDAKTQRLV 57

sp|Q7NBC4|UVRC_MYCGA --MNFNLKQKLDLAPKKPGCYLWKNHLNEIIYIGKAKNIYKRVHQYFNG-PKDLKTSKLV 57

::. * * ..:** * : :**:**** : :*: .** . . **. **

sp|P0A8G0|UVRC_ECOLI AQIQQIDVTVTHTETEALLLEHNYIKLYQPRYNVLLRDDKSYPFIFLSGDTHPRLAMHRG 120

sp|P14951|UVRC_BACSU TEIEDFEYIVTSSNLEALILEMNLIKKHDPKYNVMLKDDKTYPFIKLTHERHPRLIVTRN 117

sp|Q7NBC4|UVRC_MYCGA NEIFYVEFIEVNNENEALLLEANLIKKHKPRYNILLKDNNGYPYILMTKEKYPRLIYTRN 117

:* .: . .: ***:** * ** :.*:**::*:*:: **:* :: : :*** *.

sp|P0A8G0|UVRC_ECOLI AKHAKGEYFGPFPN-GYAVRETLALLQKIFPIRQCENSVYRNRSRPCLQYQIGRCLGPCV 179

sp|P14951|UVRC_BACSU VKKDKGRYFGPYPN-VQAARETKKLLDRLYPLRKCS----KLPDRVCLYYHLGQCLAPCV 172

sp|Q7NBC4|UVRC_MYCGA FDPKKGKHYGPFASSEMKAYDLYNLLLKLFPLKNCFN----KKGRKCEFYDLNLCMKACT 173

. **.::**:.. . : ** :::*:::* .* * *.:. *: .*.

sp|P0A8G0|UVRC_ECOLI EGLVSEEEYAQQVEYVRLFLSGKDDQVLTQLISRMETASQNLEFEEAARIRDQIQAVRRV 239

sp|P14951|UVRC_BACSU K-DISEETNRELVESITRFLRGGYNEVKKELEEKMHEAAENLEFERAKELRDQIAHIEST 231

sp|Q7NBC4|UVRC_MYCGA H-EVSEADYEVMKKKIDYFFHNGADQVLKDLKEKESIASEKFDFEQAKKYLDLQKAINLI 232

. :** : : *: . ::* .:* .: *:::::**.* . * :.

sp|P0A8G0|UVRC_ECOLI TEKQ-FVSNTGDD-LDVIGVAFDAGMACVHVLFIRQGKVLG-SRSYFPKVPGGTELSEVV 296

sp|P14951|UVRC_BACSU MEKQKMTMNDLVD-RDVFAYAYDKGWMCVQVFFIRQGKLIERDVSMFPLYQ---EADEEF 287

sp|Q7NBC4|UVRC_MYCGA FDKQIINLYSAKERIDVLAYQIKENVICIVLFSYVSSQLVSKNTICDFYYG---EEQEVI 289

:** : : **:. . . *: :: ..::: . * .* .

sp|P0A8G0|UVRC_ECOLI ETFVGQFYLQGSQMRTLPGEILLDFNLSDKTLLADSLSELAGRKINVQTKPRGDRARYLK 356

sp|P14951|UVRC_BACSU LTFIGQFYSKNN--HFLPKEILVP-DSIDQSMIEQLL------ETNVHQPKKGPKKELLM 338

sp|Q7NBC4|UVRC_MYCGA TSYLSQYYKDNIK----PKILYASLDQANATLLKDSLG------IEIINPTSGKMNEIMS 339

:::.*:* .. * : : : ::: : * :: * . :

sp|P0A8G0|UVRC_ECOLI LARTNAATALTSKLSQQSTVHQR----LTALASVLKLPEVKRMECFDISHTMGEQTVASC 412

sp|P14951|UVRC_BACSU LAHKNAKIALKEKFSLIERDEERSIGAVQKLGEALNIYTPHRIEAFDNSNIQGTNPVSAM 398

sp|Q7NBC4|UVRC_MYCGA LALQNVTNELSQKYDSLVKKEQRINLALDQLKKLIKVDKLNHLEVYDNSNLFNTDKVSAM 399

** *. *..* . .:* : * . ::: :::* :* *: . : *::

sp|P0A8G0|UVRC_ECOLI VVFDANGPLRAEYRRYNITGITPGDDYAAMNQVLRRRYGKAIDDSK--IPDVILIDGGKG 470

sp|P14951|UVRC_BACSU IVFIDGKPYKKEYRKYKIKTVTGPDDYGSMREVVRRRYTRVLRENLP-LPDLIIIDGGKG 457

sp|Q7NBC4|UVRC_MYCGA IVFENNQFNKKKYRKYKIKDQQALGDYHYMYEVIYRRLYQALKNNFVDLPDLIILDGGKH 459

:** . : :**:*:*. .** * :*: ** :.: :. :**:*::****

sp|P0A8G0|UVRC_ECOLI QLAQAKNVFAELDVSWDKNHPLLLGVAKGADRKAGLETLFFEPEGEGFSLPPDSPALHVI 530

sp|P14951|UVRC_BACSU QINAARDVIEN-ELGLDIP---IAGLAK--DEKHRTSNLLIGDPLEVAYLERNSQEFYLL 511

sp|Q7NBC4|UVRC_MYCGA QVLAAKKAIVDLQIDKKIN---LIGLAK---NNKHQTDKIVTFDLDEISLDKSSALYFFL 513

*: *:..: : ::. . : *:** .: :. : * .* ..:

sp|P0A8G0|UVRC_ECOLI QHIRDESHDHAIGGHRKKRAKVKNTSSLETIEGVGPKRRQMLLKYMGGLQGLRNASVEEI 590

sp|P14951|UVRC_BACSU QRIQDEVHRFAISFHRQIRGKSAFQSVLDDIPGIGEKRKKMLLKHFGSVKKMKEASLEDI 571

sp|Q7NBC4|UVRC_MYCGA ANLQEEVHKFAISFFRKTKAKSLYDSILDQIKGLGKKRKQQLIEHFKTIDEIKKASIASL 573

.:::* * .**. .*: :.* * *: * *:* **:: *:::: :. :::**: .:

sp|P0A8G0|UVRC_ECOLI AKVPGISQGLAEKIFWSLKH- 610

sp|P14951|UVRC_BACSU KKA-GVPAAAAQLLYDKLQK- 590

sp|Q7NBC4|UVRC_MYCGA SQV--LPIEIAKKLKQKLDQS 592

:. :. *: : .*.:

1. **DNA helicase II (gene name *uvrD*)**

>sp|P03018|UVRD_ECOLI DNA helicase II OS=Escherichia coli (strain K12) GN=uvrD PE=1 SV=1

MDVSYLLDSLNDKQREAVAAPRSNLLVLAGAGSGKTRVLVHRIAWLMSVENCSPYSIMAV

TFTNKAAAEMRHRIGQLMGTSQGGMWVGTFHGLAHRLLRAHHMDANLPQDFQILDSEDQL

RLLKRLIKAMNLDEKQWPPRQAMWYINSQKDEGLRPHHIQSYGNPVEQTWQKVYQAYQEA

CDRAGLVDFAELLLRAHELWLNKPHILQHYRERFTNILVDEFQDTNNIQYAWIRLLAGDT

GKVMIVGDDDQSIYGWRGAQVENIQRFLNDFPGAETIRLEQNYRSTSNILSAANALIENN

NGRLGKKLWTDGADGEPISLYCAFNELDEARFVVNRIKTWQDNGGALAECAILYRSNAQS

RVLEEALLQASMPYRIYGGMRFFERQEIKDALSYLRLIANRNDDAAFERVVNTPTRGIGD

RTLDVVRQTSRDRQLTLWQACRELLQEKALAGRAASALQRFMELIDALAQETADMPLHVQ

TDRVIKDSGLRTMYEQEKGEKGQTRIENLEELVTATRQFSYNEEDEDLMPLQAFLSHAAL

EAGEGQADTWQDAVQLMTLHSAKGLEFPQVFIVGMEEGMFPSQMSLDEGGRLEEERRLAY

VGVTRAMQKLTLTYAETRRLYGKEVYHRPSRFIGELPEECVEEVRLRATVSRPVSHQRMG

TPMVENDSGYKLGQRVRHAKFGEGTIVNMEGSGEHSRLQVAFQGQGIKWLVAAYARLESV

>sp|O34580|PCRA_BACSU ATP-dependent DNA helicase PcrA OS=Bacillus subtilis (strain 168) GN=pcrA PE=1 SV=1

MNYISNQLLSGLNPVQQEAVKTTDGPLLLMAGAGSGKTRVLTHRIAYLMAEKHVAPWNIL

AITFTNKAAREMKERVESILGPGADDIWISTFHSMCVRILRRDIDRIGINRNFSILDTAD

QLSVIKGILKERNLDPKKFDPRSILGTISSAKNELTEPEEFSKVAGGYYDQVVSDVYADY

QKKLLKNQSLDFDDLIMTTIKLFDRVPEVLEFYQRKFQYIHVDEYQDTNRAQYMLVKQLA

ERFQNLCVVGDSDQSIYRWRGADITNILSFEKDYPNASVILLEQNYRSTKRILRAANEVI

KNNSNRKPKNLWTENDEGIKISYYRGDNEFGEGQFVAGKIHQLHSTGKRKLSDIAILYRT

NAQSRVIEETLLKAGLNYNIVGGTKFYDRKEIKDILAYLRLVSNPDDDISFTRIVNVPKR

GVGATSLEKIASYAAINGLSFFQAIQQVDFIGVSAKAANALDSFRQMIENLTNMQDYLSI

TELTEEILDKTEYREMLKAEKSIEAQSRLENIDEFLSVTKNFEQKSEDKTLVAFLTDLAL

IADIDQLDQKEEESGGKDAITLMTLHAAKGLEFPVVFLMGLEEGVFPHSRSLMEEAEMEE

ERRLAYVGITRAEQELYLTNAKMRTLFGRTNMNPESRFIAEIPDDLLENLNEKKETRATS

ARKMQPRRGPVSRPVSYASKTGGDTLNWAVGDKAGHKKWGTGTVVSVKGEGEGTELDIAF

PSPVGVKRLLAAFAPIEKQ

>tr|Q7NB99|Q7NB99_MYCGA DNA helicase II OS=Mycoplasma gallisepticum (strain R(low / passage 15 / clone 2)) GN=uvrD PE=4 SV=2

MQDYLKSLNKQQYDVVTSDLIPIFVVAGAGTGKTKVLTSRIAYLIEHFKIPEYKILAITF

TNKAAKEMQHRLEKLLNKEKTQVSFRTFHGFCAQVLREEVNNVDRLNDRFNILDEVDQAK

LIEDLLKSQKYEYYYSQYTDFKKNKVMSIINDAKTYNLDVAEFLASDLNKLGEDHILTPN

LVQPLSNFYHDYEKALKELNAIDFNDLLNIAYNLFLNDPIILKKWQNRYEAILVDEFQDA

NEIQYKIVKLLREKNNNFLFVGDPDQSIYGWRGANSEIGDSIRFDFNDLVVKYLTQNYRS

KQSILNLANDAIKMNNSRYFKSLLSHDLTDLGPKPIWINFSNIEYQNRFVMDKIKELVAS

KQYTYGDFAILYRTNFSSVSLERLIKENRIPYEIFGGYKFFLRKEIKDLIGYLKLVDTNN

DIAFDRIINTPRRMIGDTSIEIIKELANKKSITEYEALDYLDESNIKANVKKSAQNFKKM

IEDLRANQGNWSVYQTINEIIKRINYYDYLNEPTKHDSVNEFIDFLNKYEKEYENDFGTK

LTINDFIQNLALEGDLDNNQPNHNKNALKLMTIHSAKGLEFKNVFVINMNENILPSSRSI

AATNNKAKLAEERRIVYVAYTRAKHNLWLCSNQDYDARTKEPYQPSRFLYELSDLVLDKQ

DQAKTYFDKHNFLTDDDGWFNSKKSPSKLDAWNSTNEVEHNYFVGEIIYHQLYGEGIVRE

IDDLTIKVSFKDKKAGTKDLIKNHKLITYAK

sp|P03018|UVRD_ECOLI --MDVSYLLDSLNDKQREAVAAPRSNLLVLAGAGSGKTRVLVHRIAWLMS 48

sp|O34580|PCRA_BACSU MNYISNQLLSGLNPVQQEAVKTTDGPLLLMAGAGSGKTRVLTHRIAYLMA 50

tr|Q7NB99|Q7NB99_MYCGA ----MQDYLKSLNKQQYDVVTSDLIPIFVVAGAGTGKTKVLTSRIAYLIE 46

. *..** * :.* : ::::****:***:**. ***:*:

sp|P03018|UVRD_ECOLI VENCSPYSIMAVTFTNKAAAEMRHRIGQLMGTSQGGMWVGTFHGLAHRLL 98

sp|O34580|PCRA_BACSU EKHVAPWNILAITFTNKAAREMKERVESILGPGADDIWISTFHSMCVRIL 100

tr|Q7NB99|Q7NB99_MYCGA HFKIPEYKILAITFTNKAAKEMQHRLEKLLNKEKTQVSFRTFHGFCAQVL 96

: . :.*:*:******* **:.*: .::. : . ***.:. ::*

sp|P03018|UVRD_ECOLI RAHHMDAN-LPQDFQILDSEDQLRLLKRLIKAMNLDE-----KQWPPRQA 142

sp|O34580|PCRA_BACSU RRDIDRIG-INRNFSILDTADQLSVIKGILKERNLDP-----KKFDPRSI 144

tr|Q7NB99|Q7NB99_MYCGA REEVNNVDRLNDRFNILDEVDQAKLIEDLLKSQKYEYYYSQYTDFKKNKV 146

* . . : *.*** ** ::: ::* : : ..: ..

sp|P03018|UVRD_ECOLI MWYINSQKDEGLRP-HHIQSYG----------NPVEQTWQKVYQAYQEAC 181

sp|O34580|PCRA_BACSU LGTISSAKNELTEPEEFSKVAG----------GYYDQVVSDVYADYQKKL 184

tr|Q7NB99|Q7NB99_MYCGA MSIINDAKTYNLDVAEFLASDLNKLGEDHILTPNLVQPLSNFYHDYEKAL 196

: *.. * .. * ...* *::

sp|P03018|UVRD_ECOLI DRAGLVDFAELLLRAHELWLNKPHILQHYRERFTNILVDEFQDTNNIQYA 231

sp|O34580|PCRA_BACSU LKNQSLDFDDLIMTTIKLFDRVPEVLEFYQRKFQYIHVDEYQDTNRAQYM 234

tr|Q7NB99|Q7NB99_MYCGA KELNAIDFNDLLNIAYNLFLNDPIILKKWQNRYEAILVDEFQDANEIQYK 246

. :** :*: : :*: . * :*: ::.:: * ***:**:*. **

sp|P03018|UVRD_ECOLI WIRLLAGDTGKVMIVGDDDQSIYGWRGAQVENIQRFLNDFPGAETIRLEQ 281

sp|O34580|PCRA_BACSU LVKQLAERFQNLCVVGDSDQSIYRWRGADITNILSFEKDYPNASVILLEQ 284

tr|Q7NB99|Q7NB99_MYCGA IVKLLREKNNNFLFVGDPDQSIYGWRGANSEIGDSIRFDFNDLVVKYLTQ 296

:: * :. .*** ***** ****: : *: . . * *

sp|P03018|UVRD_ECOLI NYRSTSNILSAANALIENNNGRLGKKLWTD--GADGEPISLYCAFNELDE 329

sp|O34580|PCRA_BACSU NYRSTKRILRAANEVIKNNSNRKPKNLWTE--NDEGIKISYYRGDNEFGE 332

tr|Q7NB99|Q7NB99_MYCGA NYRSKQSILNLANDAIKMNNSRYFKSLLSHDLTDLGPKPIWINFSNIEYQ 346

****.. ** ** *: *..* *.* :. * * :

sp|P03018|UVRD_ECOLI ARFVVNRIKTWQDNG-GALAECAILYRSNAQSRVLEEALLQASMPYRIYG 378

sp|O34580|PCRA_BACSU GQFVAGKIHQLHSTGKRKLSDIAILYRTNAQSRVIEETLLKAGLNYNIVG 382

tr|Q7NB99|Q7NB99_MYCGA NRFVMDKIKELVASKQYTYGDFAILYRTNFSSVSLERLIKENRIPYEIFG 396

:** .:*: . .: *****:* .* :*. : : : *.* *

sp|P03018|UVRD_ECOLI GMRFFERQEIKDALSYLRLIANRNDDAAFERVVNTPTRGIGDRTLDVVRQ 428

sp|O34580|PCRA_BACSU GTKFYDRKEIKDILAYLRLVSNPDDDISFTRIVNVPKRGVGATSLEKIAS 432

tr|Q7NB99|Q7NB99_MYCGA GYKFFLRKEIKDLIGYLKLVDTNN-DIAFDRIINTPRRMIGDTSIEIIKE 445

* :*: *:**** :.**:*: . : * :* *::*.* * :* ::: : .

sp|P03018|UVRD_ECOLI TSRDRQLTLWQACRELLQEKALAGRAASALQRFMELIDALAQETADMPLH 478

sp|O34580|PCRA_BACSU YAAINGLSFFQAIQQ-VDFIGVSAKAANALDSFRQMIENLTNMQDYLSIT 481

tr|Q7NB99|Q7NB99_MYCGA LANKKSITEYEALDY-LDESNIKANVKKSAQNFKKMIEDLRANQGNWSVY 494

: . :: ::* :: : ... .: : * ::*: * .:

sp|P03018|UVRD_ECOLI VQTDRVIKDSGLRTMYEQEKGEKGQTRIENLEELVTATRQFSYNEEDED- 527

sp|O34580|PCRA_BACSU ELTEEILDKTEYREMLKAEKSIEAQSRLENIDEFLSVTKNFEQKSEDK-- 529

tr|Q7NB99|Q7NB99_MYCGA QTINEIIKRINYYDYLN------EPTKHDSVNEFIDFLNKYEKEYENDFG 538

:.::. : :: :.::*:: .::. : *:.

sp|P03018|UVRD_ECOLI -LMPLQAFLSHAALEAGEGQADT------WQDAVQLMTLHSAKGLEFPQV 570

sp|O34580|PCRA_BACSU ---TLVAFLTDLALIADIDQLDQKEEESGGKDAITLMTLHAAKGLEFPVV 576

tr|Q7NB99|Q7NB99_MYCGA TKLTINDFIQNLALEGDLDNNQP----NHNKNALKLMTIHSAKGLEFKNV 584

.: *: . ** .. .: : ::*: ***:*:****** *

sp|P03018|UVRD_ECOLI FIVGMEEGMFPSQMSLDEG---GRLEEERRLAYVGVTRAMQKLTLTYAET 617

sp|O34580|PCRA_BACSU FLMGLEEGVFPHSRSLMEE---AEMEEERRLAYVGITRAEQELYLTNAKM 623

tr|Q7NB99|Q7NB99_MYCGA FVINMNENILPSSRSIAATNNKAKLAEERRIVYVAYTRAKHNLWLCSNQD 634

*::.::*.::* . *: ..: ****:.**. *** ::* * :

sp|P03018|UVRD_ECOLI RRLYGKEVYHRPSRFIGELPEECVEEVR----LRATVSR-------PVSH 656

sp|O34580|PCRA_BACSU RTLFGRTNMNPESRFIAEIPDDLLENLNEKKETRATSARKMQPRRGPVSR 673

tr|Q7NB99|Q7NB99_MYCGA YDARTKEPYQ-PSRFLYELSDLVLDKQDQAKTYFDKHNFLTDDDGWFNSK 683

: : ***: *:.: ::: . *:

sp|P03018|UVRD_ECOLI QRMGTPMVENDSG-YKLGQRVRHAKFGEGTIVNMEGSGEHSRLQVAFQG- 704

sp|O34580|PCRA_BACSU PVSYASKTGGDTLNWAVGDKAGHKKWGTGTVVSVKGEGEGTELDIAFPSP 723

tr|Q7NB99|Q7NB99_MYCGA KSPSKLDAWNSTNEVEHNYFVGEIIYHQLYGEGIVREIDDLTIKVSFKDK 733

. ..: . . . : .: . : :.::* .

sp|P03018|UVRD_ECOLI -QGIKWLVAAYARLESV- 720

sp|O34580|PCRA_BACSU -VGVKRLLAAFAPIEKQ- 739

tr|Q7NB99|Q7NB99_MYCGA KAGTKDLIKNHKLITYAK 751

* * *: . :

1. **Formamidopyrimidine-DNA glycosylase (gene name *fpg (mutM)*)**

>sp|P05523|FPG_ECOLI Formamidopyrimidine-DNA glycosylase OS=Escherichia coli (strain K12) GN=mutM PE=1 SV=3

MPELPEVETSRRGIEPHLVGATILHAVVRNGRLRWPVSEEIYRLSDQPVLSVQRRAKYLL

LELPEGWIIIHLGMSGSLRILPEELPPEKHDHVDLVMSNGKVLRYTDPRRFGAWLWTKEL

EGHNVLTHLGPEPLSDDFNGEYLHQKCAKKKTAIKPWLMDNKLVVGVGNIYASESLFAAG

IHPDRLASSLSLAECELLARVIKAVLLRSIEQGGTTLKDFLQSDGKPGYFAQELQVYGRK

GEPCRVCGTPIVATKHAQRATFYCRQCQK

>sp|O34403|FPG_BACSU Formamidopyrimidine-DNA glycosylase OS=Bacillus subtilis (strain 168) GN=mutM PE=3 SV=4

MPELPEVETVRRTLTGLVKGKTIKSVEIRWPNIIKRPAEPEEFARKLAGETIQSIGRRGK

FLLFHLDHYVMVSHLRMEGKYGLHQAEEPDDKHVHVIFTMTDGTQLRYRDVRKFGTMHLF

KPGEEAGELPLSQLGPEPDAEEFTSAYLKDRLAKTNRAVKTALLDQKTVVGLGNIYVDEA

LFRAGVHPETKANQLSDKTIKTLHAEIKNTLQEAIDAGGSTVRSYINSQGEIGMFQLQHF

VYGKKDEPCKNCGTMISKIVVGGRGTHFCAKCQTKK

>tr|Q7NBN4|Q7NBN4_MYCGA Formamidopyrimidine-DNA glycosylase OS=Mycoplasma gallisepticum (strain R(low / passage 15 / clone 2)) GN=mutM PE=3 SV=2

MPELPEVQTVINYLKTKIINQKINNVIVSALKVLKNATAKEFKKFLVNEHFVDIKRIGKY

IIFILSNNKVLVSHLRMEGKYKISQFKAKYDERHVLVRFILDDFELHYHDTRRFGTFHIH

SVLDYQDQDYLKKLAIDPTQQEWDWKYLKNNAQKSSRVIKSVLLDQSVVAGIGNIYADEI

LFLSKINPAKKANELTDQQFKEISKNATKVLLKAIELNGTTIFSYQFKENHAGSYQDYLN

VHLQKDKPCKVCGNLVKKTKLNNRGTYYCAKCQK

sp|P05523|FPG_ECOLI MPELPEVETSRRGIEPHLVGATILHAVVRN-GRLRWPVSEEIYR--LSDQ 47

sp|O34403|FPG_BACSU MPELPEVETVRRTLTGLVKGKTIKSVEIRWPNIIKRPAEPEEFARKLAGE 50

tr|Q7NBN4|Q7NBN4_MYCGA MPELPEVQTVINYLKTKIINQKINNVIVSALKVLKN-ATAKEFKKFLVNE 49

*******:* . : : . .* . : :: . : : * .:

sp|P05523|FPG_ECOLI PVLSVQRRAKYLLLELPEG-WIIIHLGMSGSLRILPEELPPEKHDHVDLV 96

sp|O34403|FPG_BACSU TIQSIGRRGKFLLFHLDHY-VMVSHLRMEGKYGLHQAEEPDDKHVHVIFT 99

tr|Q7NBN4|Q7NBN4_MYCGA HFVDIKRIGKYIIFILSNNKVLVSHLRMEGKYKISQFKAKYDERHVLVRF 99

. .: * .*:::: * . :: ** *.*. : : ::: :

sp|P05523|FPG_ECOLI MSNGKVLRYTDPRRFGAWLWTK--ELEGHNVLTHLGPEPLSDDFNGEYLH 144

sp|O34403|FPG_BACSU MTDGTQLRYRDVRKFGTMHLFKPGEEAGELPLSQLGPEPDAEEFTSAYLK 149

tr|Q7NBN4|Q7NBN4_MYCGA ILDDFELHYHDTRRFGTFHIHSVLDYQDQDYLKKLAIDPTQQEWDWKYLK 149

: :. *:* * *:**: . : .. *.:*. :* ::: **:

sp|P05523|FPG_ECOLI QKCAKKKTAIKPWLMDNKLVVGVGNIYASESLFAAGIHPDRLASSLSLAE 194

sp|O34403|FPG_BACSU DRLAKTNRAVKTALLDQKTVVGLGNIYVDEALFRAGVHPETKANQLSDKT 199

tr|Q7NBN4|Q7NBN4_MYCGA NNAQKSSRVIKSVLLDQSVVAGIGNIYADEILFLSKINPAKKANELTDQQ 199

:. *.. .:*. *:*:. *.*:****..* ** : ::* *..*:

sp|P05523|FPG_ECOLI CELLARVIKAVLLRSIEQGGTTLKDFLQSDGKPGYFAQELQVYGRKGEPC 244

sp|O34403|FPG_BACSU IKTLHAEIKNTLQEAIDAGGSTVRSYINSQGEIGMFQLQHFVYGKKDEPC 249

tr|Q7NBN4|Q7NBN4_MYCGA FKEISKNATKVLLKAIELNGTTIFSYQFKENHAGSYQDYLNVHLQKDKPC 249

: : . .* .:*: .*:*: .: .:.. * : *: :*.:**

sp|P05523|FPG_ECOLI RVCGTPIVATKHAQRATFYCRQCQK-- 269

sp|O34403|FPG_BACSU KNCGTMISKIVVGGRGTHFCAKCQTKK 276

tr|Q7NBN4|Q7NBN4_MYCGA KVCGNLVKKTKLNNRGTYYCAKCQK-- 274

: **. : *.*.:* :**.

1. **Uracil-DNA glycosylase (gene name *ung*)**

>sp|P12295|UNG_ECOLI Uracil-DNA glycosylase OS=Escherichia coli (strain K12) GN=ung PE=1 SV=2

MANELTWHDVLAEEKQQPYFLNTLQTVASERQSGVTIYPPQKDVFNAFRFTELGDVKVVI

LGQDPYHGPGQAHGLAFSVRPGIAIPPSLLNMYKELENTIPGFTRPNHGYLESWARQGVL

LLNTVLTVRAGQAHSHASLGWETFTDKVISLINQHREGVVFLLWGSHAQKKGAIIDKQRH

HVLKAPHPSPLSAHRGFFGCNHFVLANQWLEQRGETPIDWMPVLPAESE

>sp|P39615|UNG_BACSU Uracil-DNA glycosylase OS=Bacillus subtilis (strain 168) GN=ung PE=1 SV=1

MKQLLQDSWWNQLKEEFEKPYYQELREMLKREYAEQTIYPDSRDIFNALHYTSYDDVKVV

ILGQDPYHGPGQAQGLSFSVKPGVKQPPSLKNIFLELQQDIGCSIPNHGSLVSWAKQGVL

LLNTVLTVRRGQANSHKGKGWERLTDRIIDVLSERERPVIFILWGRHAQMKKERIDTSKH

FIIESTHPSPFSARNGFFGSRPFSRANAYLEKMGEAPIDWCIKDL

>tr|Q7NAM7|Q7NAM7_MYCGA Uracil-DNA glycosylase OS=Mycoplasma gallisepticum (strain R(low / passage 15 / clone 2)) GN=ung PE=3 SV=1

MLEQLIGEIQTNWKDLINQFFATHKTIYHQLDQLIKNRSEKNELIPKKELIFNAFNFFDY

QETKVVIIGQDPYADLKKANGLAFGVDNNNPPVSLRNIIKELINNLKLDEQQLDDFDYSL

KSWANQGVLLINTILTVKKQNPLSDQNLGWEELIKFLILKLLENQTQPVFVLWGKKAQGF

LEPYQLKHVLKSAHPSFFSAKQFFNNNHFNLINELLKTKNEQLIQWVKQNK

sp|P12295|UNG_ECOLI ----MANELT--WHDVLAEEKQ-QPYFLNTLQTVASERQSGVTIYPPQKD 43

sp|P39615|UNG_BACSU ----MKQLLQDSWWNQLKEEFE-KPYYQ-ELREMLKREYAEQTIYPDSRD 44

tr|Q7NAM7|Q7NAM7_MYCGA MLEQLIGEIQTNWKDLINQFFATHKTIYHQLDQLIKNRSEKNELIPKKEL 50

: : * : : : : * : ... : * ..

sp|P12295|UNG_ECOLI VFNAFRFTELGDVKVVILGQDPYHGPGQAHGLAFSVRPGIAIPPSLLNMY 93

sp|P39615|UNG_BACSU IFNALHYTSYDDVKVVILGQDPYHGPGQAQGLSFSVKPGVKQPPSLKNIF 94

tr|Q7NAM7|Q7NAM7_MYCGA IFNAFNFFDYQETKVVIIGQDPYADLKKANGLAFGVDNNNP-PVSLRNII 99

:***:.: . :.****:***** . :*:**:*.* . * ** *:

sp|P12295|UNG_ECOLI KELENTIPGFTRPNHG---YLESWARQGVLLLNTVLTVRAGQAHSHASLG 140

sp|P39615|UNG_BACSU LELQQDI-GCSIPNHG---SLVSWAKQGVLLLNTVLTVRRGQANSHKGKG 140

tr|Q7NAM7|Q7NAM7_MYCGA KELINNLKLDEQQLDDFDYSLKSWANQGVLLINTILTVKKQNPLSDQNLG 149

** : : .. * ***.*****:**:***: :. *. . *

sp|P12295|UNG_ECOLI WETFTDKVISLINQHREGVVFLLWGSHAQKKGAIIDKQRHHVLKAPHPSP 190

sp|P39615|UNG_BACSU WERLTDRIIDVLSERERPVIFILWGRHAQMKKERIDTSKHFIIESTHPSP 190

tr|Q7NAM7|Q7NAM7_MYCGA WEELIKFLILKLLENQTQPVFVLWGKKAQG--FLEPYQLKHVLKSAHPSF 197

** : . :* : :.. :*:*** :** . :.::::.***

sp|P12295|UNG_ECOLI LSAHRGFFGCNHFVLANQWLEQRGETPIDWMPVLPAESE 229

sp|P39615|UNG_BACSU FSARNGFFGSRPFSRANAYLEKMGEAPIDWCIKDL---- 225

tr|Q7NAM7|Q7NAM7_MYCGA FSAKQ-FFNNNHFNLINELLKTKNEQLIQWVKQNK---- 231

:**:. **. . * * *: .* *:*

| **Organism** | **Active site (Proton acceptor)** |
| --- | --- |
| E. coli (strain K12) | D64 |
| B. subtilis (strain 168) | D65 |
| M. gallisepticum (strain R(low / passage 15 / clone 2)) | No information |

1. **Endonuclease IV (gene name *nfo*)**

>sp|P0A6C1|END4_ECOLI Endonuclease 4 OS=Escherichia coli (strain K12) GN=nfo PE=1 SV=1

MKYIGAHVSAAGGLANAAIRAAEIDATAFALFTKNQRQWRAAPLTTQTIDEFKAACEKYH

YTSAQILPHDSYLINLGHPVTEALEKSRDAFIDEMQRCEQLGLSLLNFHPGSHLMQISEE

DCLARIAESINIALDKTQGVTAVIENTAGQGSNLGFKFEHLAAIIDGVEDKSRVGVCIDT

CHAFAAGYDLRTPAECEKTFADFARTVGFKYLRGMHLNDAKSTFGSRVDRHHSLGEGNIG

HDAFRWIMQDDRFDGIPLILETINPDIWAEEIAWLKAQQTEKAVA

>sp|P54476|END4_BACSU Probable endonuclease 4 OS=Bacillus subtilis (strain 168) GN=nfo PE=3 SV=1

MLRIGSHVSMSGKHMLLAASQEAVSYGANTFMIYTGAPQNTRRKKIEDLNIEAGRAHMQE

NGIDEIIVHAPYIINIGNTTNPSTFELGVDFLRSEIERTAAIGAKQIVLHPGAHVGAGAE

AGIKKIIEGLNEVIDPNQNVQIALETMAGKGSECGRSFEELAQIIEGVTHNEQLSVCFDT

CHTHDAGYNIVEDFDGVLNEFDKIIGIDRIKVLHINDSKNVKGARKDRHENIGFGEIGFD

ALQYVVHHEQLKDIPKILETPYVGEDKKNKKPPYRFEIEMLKEKQFDDTLLEKILQQ

>sp|Q7NBA9|END4_MYCGA Probable endonuclease 4 OS=Mycoplasma gallisepticum (strain R(low / passage 15 / clone 2)) GN=nfo PE=3 SV=1

MKSNKIKYLGCFVGATKPDFMLGMVKTVVDYGATSFMFYSGPPQSFRRTPTAQFKLDLAK

AYLAKHNLGDLGDNYVVHAPYLINLANGDSTKRERSFNFFLDELKRTNELGAKYFVLHPG

SALNVKDKTQALDHLATELNRAISMTKDTIICLETMADKGQQICSKFEELRYVIDQISDK

SRIGVCFDTCHVHDAGYDLAKTQELIDHFDQVIGLKYLYVIHLNDSKNPMGARKDRHANI

GYGKIGFENLLNFIYHKEICNKIIILETPWIDDPIRGEVPLYKEEIEMIRNKKFVEGLVN

EES

sp|P54476|END4_BACSU -----MLRIGSHVSMSGKHMLLAASQEAVSYGANTFMIYTGAPQNTRRKKIEDLNIEAGR 55

sp|Q7NBA9|END4_MYCGA MKSNKIKYLGCFVGATKPDFMLGMVKTVVDYGATSFMFYSGPPQSFRRTPTAQFKLDLAK 60

sp|P0A6C1|END4_ECOLI -----MKYIGAHVSAAGG--LANAAIRAAEIDATAFALFTKNQRQWRAAPLTTQTIDEFK 53

: :*..*. : : ... .*.:* ::: :. * .:: :

sp|P54476|END4_BACSU AHMQENGI----DEIIVHAPYIINIGNTTNPSTFELGVDFLRSEIERTAAIGAKQIVLHP 111

sp|Q7NBA9|END4_MYCGA AYLAKHNLGDLGDNYVVHAPYLINLANG-DSTKRERSFNFFLDELKRTNELGAKYFVLHP 119

sp|P0A6C1|END4_ECOLI AACEKYHYTS--AQILPHDSYLINLGHP-VTEALEKSRDAFIDEMQRCEQLGLSLLNFHP 110

* : : : * .*:**:.: . * . : : .*::* :* . : :**

sp|P54476|END4_BACSU GAHVGAG-AEAGIKKIIEGLNEVIDPNQNVQIALETMAGKGSECGRSFEELAQIIEGVTH 170

sp|Q7NBA9|END4_MYCGA GSALNVKDKTQALDHLATELNRAISMTKDTIICLETMADKGQQICSKFEELRYVIDQISD 179

sp|P0A6C1|END4_ECOLI GSHLMQISEEDCLARIAESINIALDKTQGVTAVIENTAGQGSNLGFKFEHLAAIIDGVED 170

*: : : :: :* .:. .:.. :*. *.:*.: .**.* :*: : .

sp|P54476|END4_BACSU NEQLSVCFDTCHTHDAGYNI--VEDFDGVLNEFDKIIGIDRIKVLHINDSKNVKGARKDR 228

sp|Q7NBA9|END4_MYCGA KSRIGVCFDTCHVHDAGYDL--AKTQE-LIDHFDQVIGLKYLYVIHLNDSKNPMGARKDR 236

sp|P0A6C1|END4_ECOLI KSRVGVCIDTCHAFAAGYDLRTPAECEKTFADFARTVGFKYLRGMHLNDAKSTFGSRVDR 230

:.::.**:****.. ***:: : : .* : :*:. : :*:**:*. *:* **

sp|P54476|END4_BACSU HENIGFGEIGFDALQYVVHHEQLKDIPKILETPYVGEDKKNKKPPYRFEIEMLKEKQFDD 288

sp|Q7NBA9|END4_MYCGA HANIGYGKIGFENLLNFIYHKEICNKIIILETPWIDDPIRGEVPLYKEEIEMIRNKKFVE 296

sp|P0A6C1|END4_ECOLI HHSLGEGNIGHDAFRWIMQDDRFDGIPLILETINPD--------IWAEEIAWLKAQQTEK 282

* .:* *:**.: : .: ...: . **** . : ** :: :: .

sp|P54476|END4_BACSU TLLEKILQQ 297

sp|Q7NBA9|END4_MYCGA GLVNEES-- 303

sp|P0A6C1|END4_ECOLI AVA------ 285

1. **Recombinase RecA (gene name *recA*)**

>sp|P0A7G6|RECA_ECOLI Protein RecA OS=Escherichia coli (strain K12) GN=recA PE=1 SV=2

MAIDENKQKALAAALGQIEKQFGKGSIMRLGEDRSMDVETISTGSLSLDIALGAGGLPMG

RIVEIYGPESSGKTTLTLQVIAAAQREGKTCAFIDAEHALDPIYARKLGVDIDNLLCSQP

DTGEQALEICDALARSGAVDVIVVDSVAALTPKAEIEGEIGDSHMGLAARMMSQAMRKLA

GNLKQSNTLLIFINQIRMKIGVMFGNPETTTGGNALKFYASVRLDIRRIGAVKEGENVVG

SETRVKVVKNKIAAPFKQAEFQILYGEGINFYGELVDLGVKEKLIEKAGAWYSYKGEKIG

QGKANATAWLKDNPETAKEIEKKVRELLLSNPNSTPDFSVDDSEGVAETNEDF

>sp|P16971|RECA_BACSU Protein RecA OS=Bacillus subtilis (strain 168) GN=recA PE=1 SV=2

MSDRQAALDMALKQIEKQFGKGSIMKLGEKTDTRISTVPSGSLALDTALGIGGYPRGRII

EVYGPESSGKTTVALHAIAEVQQQGGQAAFIDAEHALDPVYAQKLGVNIEELLLSQPDTG

EQALEIAEALVRSGAVDIVVVDSVAALVPKAEIEGDMGDSHVGLQARLMSQALRKLSGAI

NKSKTIAIFINQIREKVGVMFGNPETTPGGRALKFYSSVRLEVRRAEQLKQGNDVMGNKT

KIKVVKNKVAPPFRTAEVDIMYGEGISKEGEIIDLGTELDIVQKSGSWYSYEEERLGQGR

ENAKQFLKENKDIMLMIQEQIREHYGLDNNGVVQQQAEETQEELEFEE

>tr|F8WJY9|F8WJY9_MYCGA Protein RecA OS=Mycoplasma gallisepticum (strain R(low / passage 15 / clone 2)) GN=recA PE=3 SV=1

MFNKKNYDKIMNNIKNSKRNNDRITNITNYDVLKLLQQKFGKTNIYLNEKDELKDLEAIS

TGSIKLDHALGTDGFIKGRIVEIYGNESCGKTTLALSTIKQAIDRNMRVAFIDAEHALDL

RYVKRLGIDLTKLIIARPDYGEQGFEIIKSLIKTELIDLIVVDSVAALVPKVEIEGKMED

QTMGTHARMMSRGLSRIQPLLAKHNVSVIFINQLREKVGIMFGNPEVTTGGKALKFYSST

RLELRRAEIIKDAANNAIGIRSKATITKNKLSTPMTTTYIDFYFKSGISEVNEIIDLAID

YQIIEQSGSWFSYQKEKIAQGKANLITKLGESEELYKLIKTEVLNKLKDCQ

sp|P0A7G6|RECA_ECOLI ----------------MAIDENKQKALA--AALGQIEKQFGKGSIMRLGE 32

sp|P16971|RECA_BACSU -------------------MSDRQAALD--MALKQIEKQFGKGSIMKLGE 29

tr|F8WJY9|F8WJY9_MYCGA MFNKKNYDKIMNNIKNSKRNNDRITNITNYDVLKLLQQKFGKTNIYLNEK 50

.:: : .* ::::*** .* :

sp|P0A7G6|RECA_ECOLI DRSMDVETISTGSLSLDIALGAGGLPMGRIVEIYGPESSGKTTLTLQVIA 82

sp|P16971|RECA_BACSU KTDTRISTVPSGSLALDTALGIGGYPRGRIIEVYGPESSGKTTVALHAIA 79

tr|F8WJY9|F8WJY9_MYCGA DELKDLEAISTGSIKLDHALGTDGFIKGRIVEIYGNESCGKTTLALSTIK 100

. :.::.:**: ** *** .* ***:*:** **.****::* .*

sp|P0A7G6|RECA_ECOLI AAQREGKTCAFIDAEHALDPIYARKLGVDIDNLLCSQPDTGEQALEICDA 132

sp|P16971|RECA_BACSU EVQQQGGQAAFIDAEHALDPVYAQKLGVNIEELLLSQPDTGEQALEIAEA 129

tr|F8WJY9|F8WJY9_MYCGA QAIDRNMRVAFIDAEHALDLRYVKRLGIDLTKLIIARPDYGEQGFEIIKS 150

. .. ********** *.::**::: :*: ::** ***.:** .:

sp|P0A7G6|RECA_ECOLI LARSGAVDVIVVDSVAALTPKAEIEGEIGDSHMGLAARMMSQAMRKLAGN 182

sp|P16971|RECA_BACSU LVRSGAVDIVVVDSVAALVPKAEIEGDMGDSHVGLQARLMSQALRKLSGA 179

tr|F8WJY9|F8WJY9_MYCGA LIKTELIDLIVVDSVAALVPKVEIEGKMEDQTMGTHARMMSRGLSRIQPL 200

* :: :*::********.**.****.: *. :* **:**:.: ::

sp|P0A7G6|RECA_ECOLI LKQSNTLLIFINQIRMKIGVMFGNPETTTGGNALKFYASVRLDIRRIGAV 232

sp|P16971|RECA_BACSU INKSKTIAIFINQIREKVGVMFGNPETTPGGRALKFYSSVRLEVRRAEQL 229

tr|F8WJY9|F8WJY9_MYCGA LAKHNVSVIFINQLREKVGIMFGNPEVTTGGKALKFYSSTRLELRRAEII 250

: : :. *****:* *:*:******.*.**.*****:*.**::** :

sp|P0A7G6|RECA_ECOLI KEGEN-VVGSETRVKVVKNKIAAPFKQAEFQILYGEGINFYGELVDLGVK 281

sp|P16971|RECA_BACSU KQGND-VMGNKTKIKVVKNKVAPPFRTAEVDIMYGEGISKEGEIIDLGTE 278

tr|F8WJY9|F8WJY9_MYCGA KDAANNAIGIRSKATITKNKLSTPMTTTYIDFYFKSGISEVNEIIDLAID 300

*:. : .:* .:: .:.***::.*: : .:: : .**. .*::**. .

sp|P0A7G6|RECA_ECOLI EKLIEKAGAWYSYKGEKIGQGKANATAWLKDNPETAKEIEKKVRELLLSN 331

sp|P16971|RECA_BACSU LDIVQKSGSWYSYEEERLGQGRENAKQFLKENKDIMLMIQEQIREHYGLD 328

tr|F8WJY9|F8WJY9_MYCGA YQIIEQSGSWFSYQKEKIAQGKANLITKLGESEELYKLIKTEVLN----- 345

.:::::*:*:**: *::.**: * * :. : *: :: :

sp|P0A7G6|RECA_ECOLI PNSTPDFSVDDSEGVAETNEDF 353

sp|P16971|RECA_BACSU NNGVVQQQAEETQEELEFEE-- 348

tr|F8WJY9|F8WJY9_MYCGA -------KLKDCQ--------- 351

. .: :

1. **Holliday junction ATP-dependent DNA helicase subunit A (gene name *ruvA*)**

>sp|P0A809|RUVA_ECOLI Holliday junction ATP-dependent DNA helicase RuvA OS=Escherichia coli (strain K12) GN=ruvA PE=1 SV=1

MIGRLRGIIIEKQPPLVLIEVGGVGYEVHMPMTCFYELPEAGQEAIVFTHFVVREDAQLL

YGFNNKQERTLFKELIKTNGVGPKLALAILSGMSAQQFVNAVEREEVGALVKLPGIGKKT

AERLIVEMKDRFKGLHGDLFTPAADLVLTSPASPATDDAEQEAVAALVALGYKPQEASRM

VSKIARPDASSETLIREALRAAL

>sp|O05392|RUVA_BACSU Holliday junction ATP-dependent DNA helicase RuvA OS=Bacillus subtilis (strain 168) GN=ruvA PE=3 SV=2

MIEFVKGTIDYVSPQYIVIENGGIGYQIFTPNPFIYKERSQETIFTYHHIREDAFSLYGF

STREEKALFTKLLNVTGIGPKGALAILGSGDPGAVIQAIENEDEAFLVKFPGVGKKTARQ

IILDLKGKLADVVPEMIENLFNHEERLEKQTAETALEEALEALRVLGYAEKEIKKVLPHL

KEEIGLTTDQYVKKALQKLLK

>sp|Q7NAN2|RUVA_MYCGA Holliday junction ATP-dependent DNA helicase RuvA OS=Mycoplasma gallisepticum (strain R(low / passage 15 / clone 2)) GN=ruvA PE=3 SV=1

MITSVYAKIEYVTNTKMLFVANNWGYWVNVKPNSGFSRTDNNVLVFLHELTFLAQNNAIN

KELYAFKSLKEKEWFKALLTINGIGPKTAMNVMVNKQEEVLTLIKNNDLNGLLRLENINK

KVATMLLASDIASKHYLKNQIVVSDKVEPQIDDDEKIDDSKDLNDDELLSEIVIEAIDCL

ISLGYKQEQIKTALAEIDLKNESINDSADLVAVIIKQIGLRTSEVS

sp|P0A809|RUVA_ECOLI MIGRLRGIIIEKQPPLVLIEVGGVGYEVHMPMTCFYELPEAGQEAIVFTHFVVREDAQL- 59

sp|O05392|RUVA_BACSU MIEFVKGTIDYVSPQYIVIENGGIGYQIFTPNPFIYKE---RSQETIFTYHHIREDAFS- 56

sp|Q7NAN2|RUVA_MYCGA MITSVYAKIEYVTNTKMLFVANNWGYWVNVKPNSGFSRTDNNVLVFLHELTFLAQNNAIN 60

** : . * ::: .. ** : :. :. : ::

sp|P0A809|RUVA_ECOLI --LYGFNNKQERTLFKELIKTNGVGPKLALAILSGMSAQQFVNAVEREEVGALVKLPGIG 117

sp|O05392|RUVA_BACSU --LYGFSTREEKALFTKLLNVTGIGPKGALAILGSGDPGAVIQAIENEDEAFLVKFPGVG 114

sp|Q7NAN2|RUVA_MYCGA KELYAFKSLKEKEWFKALLTINGIGPKTAMNVMVN-KQEEVLTLIKNNDLNGLLRLENIN 119

**.*.. :*: *. *:. .*:*** *: :: . . .: ::.:: *::: .:.

sp|P0A809|RUVA_ECOLI KKTAERLIVEMKD-----RFKGLHGDLFTPAADLVLTSPASPATDD------AEQEAVAA 166

sp|O05392|RUVA_BACSU KKTARQIILDLKG-----KLADVVPEMIENLFNHEER-LEKQTAET------ALEEALEA 162

sp|Q7NAN2|RUVA_MYCGA KKVATMLLASDIASKHYLKNQIVVSDKVEPQIDDDEKIDDSKDLNDDELLSEIVIEAIDC 179

**.* :: . : : : . : . : **: .

sp|P0A809|RUVA_ECOLI LVALGYKPQEASRMVSKIARPD---ASSETLIREALRAAL------- 203

sp|O05392|RUVA_BACSU LRVLGYAEKEIKKVLPHLKEEIG--LTTDQYVKKALQKLLK------ 201

sp|Q7NAN2|RUVA_MYCGA LISLGYKQEQIKTALAEIDLKNESINDSADLVAVIIKQIGLRTSEVS 226

* *** :: . :..: : : ::

1. **Holliday junction ATP-dependent DNA helicase subunit B (gene name *ruvB*)**

>sp|P0A812|RUVB_ECOLI Holliday junction ATP-dependent DNA helicase RuvB OS=Escherichia coli (strain K12) GN=ruvB PE=1 SV=1

MIEADRLISAGTTLPEDVADRAIRPKLLEEYVGQPQVRSQMEIFIKAAKLRGDALDHLLI

FGPPGLGKTTLANIVANEMGVNLRTTSGPVLEKAGDLAAMLTNLEPHDVLFIDEIHRLSP

VVEEVLYPAMEDYQLDIMIGEGPAARSIKIDLPPFTLIGATTRAGSLTSPLRDRFGIVQR

LEFYQVPDLQYIVSRSARFMGLEMSDDGALEVARRARGTPRIANRLLRRVRDFAEVKHDG

TISADIAAQALDMLNVDAEGFDYMDRKLLLAVIDKFFGGPVGLDNLAAAIGEERETIEDV

LEPYLIQQGFLQRTPRGRMATTRAWNHFGITPPEMP

>sp|O32055|RUVB_BACSU Holliday junction ATP-dependent DNA helicase RuvB OS=Bacillus subtilis (strain 168) GN=ruvB PE=1 SV=2

MDERLVSSEADNHESVIEQSLRPQNLAQYIGQHKVKENLRVFIDAAKMRQETLDHVLLYG

PPGLGKTTLASIVANEMGVELRTTSGPAIERPGDLAAILTALEPGDVLFIDEIHRLHRSI

EEVLYPAMEDFCLDIVIGKGPSARSVRLDLPPFTLVGATTRVGLLTAPLRDRFGVMSRLE

YYTQEELADIVTRTADVFEVEIDKPSALEIARRSRGTPRVANRLLRRVRDFAQVLGDSRI

TEDISQNALERLQVDRLGLDHIDHKLLMGMIEKFNGGPVGLDTISATIGEESHTIEDVYE

PYLLQIGFIQRTPRGRIVTPAVYHHFQMEAPRYD

>tr|Q7NAN3|Q7NAN3_MYCGA Holliday junction ATP-dependent DNA helicase RuvB OS=Mycoplasma gallisepticum (strain R(low / passage 15 / clone 2)) GN=ruvB PE=3 SV=1

MKLARPNNFDEFIGKNELKQKLLTFINASISQNKALDHVLFYGPPGVGKTSLAQIIANEL

KSKIKILQASQIQKPADLLNAFSLLSKNDVLFIDEIHSLSPTIMELLFPIMEDYVVDILI

GKEFNSKFTRMKLPPFTLIGATTMYGRIIDPLEERFGILLQLDYYQDDEIFEIIRSINAK

EKIKLTKDEMVQIAEHSKGTPRNALRIYKRVMDFKLFDQEITIKSILEKLNIYQYGLSNL

DLEYLKSFDDNPKLYLGLKSLSLISGIDCFTIESKIEPYLLKMNLIKKTSKGRQITQKAI

QYFKDN

sp|P0A812|RUVB_ECOLI MIEADRLISAGTTLPEDVADRAIRPKLLEEYVGQPQVRSQMEIFIKAAKL 50

sp|O32055|RUVB_BACSU --MDERLVSSEADNHESVIEQSLRPQNLAQYIGQHKVKENLRVFIDAAKM 48

tr|Q7NAN3|Q7NAN3_MYCGA -------------------MKLARPNNFDEFIGKNELKQKLLTFINASIS 31

: **: : :::*: :::.:: **.*:

sp|P0A812|RUVB_ECOLI RGDALDHLLIFGPPGLGKTTLANIVANEMGVNLRTTSGPVLEKAGDLAAM 100

sp|O32055|RUVB_BACSU RQETLDHVLLYGPPGLGKTTLASIVANEMGVELRTTSGPAIERPGDLAAI 98

tr|Q7NAN3|Q7NAN3_MYCGA QNKALDHVLFYGPPGVGKTSLAQIIANELKSKIKILQASQIQKPADLLNA 81

: .:***:*::****:***:**.*:***: ::: ... :::..**

sp|P0A812|RUVB_ECOLI LTNLEPHDVLFIDEIHRLSPVVEEVLYPAMEDYQLDIMIGEGPAARSIKI 150

sp|O32055|RUVB_BACSU LTALEPGDVLFIDEIHRLHRSIEEVLYPAMEDFCLDIVIGKGPSARSVRL 148

tr|Q7NAN3|Q7NAN3_MYCGA FSLLSKNDVLFIDEIHSLSPTIMELLFPIMEDYVVDILIGKEFNSKFTRM 131

:: *. ********* * : *:*:* ***: :**:**: :: ::

sp|P0A812|RUVB_ECOLI DLPPFTLIGATTRAGSLTSPLRDRFGIVQRLEFYQVPDLQYIVSRSARFM 200

sp|O32055|RUVB_BACSU DLPPFTLVGATTRVGLLTAPLRDRFGVMSRLEYYTQEELADIVTRTADVF 198

tr|Q7NAN3|Q7NAN3_MYCGA KLPPFTLIGATTMYGRIIDPLEERFGILLQLDYYQDDEIFEIIRSINAKE 181

.******:**** * : **.:***:: :*::* :: *:

sp|P0A812|RUVB_ECOLI GLEMSDDGALEVARRARGTPRIANRLLRRVRDFAEVKHDGTISADIAAQA 250

sp|O32055|RUVB_BACSU EVEIDKPSALEIARRSRGTPRVANRLLRRVRDFAQVLGDSRITEDISQNA 248

tr|Q7NAN3|Q7NAN3_MYCGA KIKLTKDEMVQIAEHSKGTPRNALRIYKRVMDFKLFDQEITIKS-----I 226

::: . :::*.:::**** * *: :** ** . : *.

sp|P0A812|RUVB_ECOLI LDMLNVDAEGFDYMDRKLLLAVIDKFFGGPVGLDNLAAAIGEERETIEDV 300

sp|O32055|RUVB_BACSU LERLQVDRLGLDHIDHKLLMGMIEKFNGGPVGLDTISATIGEESHTIEDV 298

tr|Q7NAN3|Q7NAN3_MYCGA LEKLNIYQYGLSNLDLEYLKSFDDNPK-LYLGLKSLSLISGIDCFTIESK 275

*: *:: *:. :* : * .. :: :**..:: * : ***.

sp|P0A812|RUVB_ECOLI LEPYLIQQGFLQRTPRGRMATTRAWNHFGITPPEMP 336

sp|O32055|RUVB_BACSU YEPYLLQIGFIQRTPRGRIVTPAVYHHFQMEAPRYD 334

tr|Q7NAN3|Q7NAN3_MYCGA IEPYLLKMNLIKKTSKGRQITQKAIQYFKDN----- 306

****:: .::::*.:** * . ::*

1. **Chromosome cohesion (gene name *smc*)**

Gene is absent in *E. coli* (strain K12) genome.

>sp|P51834|SMC_BACSU Chromosome partition protein Smc OS=Bacillus subtilis (strain 168) GN=smc PE=1 SV=3

MFLKRLDVIGFKSFAERISVDFVKGVTAVVGPNGSGKSNITDAIRWVLGEQSARSLRGGK

MEDIIFAGSDSRKRLNLAEVTLTLDNDDHFLPIDFHEVSVTRRVYRSGESEFLINNQPCR

LKDIIDLFMDSGLGKEAFSIISQGKVEEILSSKAEDRRSIFEEAAGVLKYKTRKKKAENK

LFETQDNLNRVEDILHELEGQVEPLKIQASIAKDYLEKKKELEHVEIALTAYDIEELHGK

WSTLKEKVQMAKEEELAESSAISAKEAKIEDTRDKIQALDESVDELQQVLLVTSEELEKL

EGRKEVLKERKKNAVQNQEQLEEAIVQFQQKETVLKEELSKQEAVFETLQAEVKQLRAQV

KEKQQALSLHNENVEEKIEQLKSDYFELLNSQASIRNELQLLDDQMSQSAVTLQRLADNN

EKHLQERHDISARKAACETEFARIEQEIHSQVGAYRDMQTKYEQKKRQYEKNESALYQAY

QYVQQARSKKDMLETMQGDFSGFYQGVKEVLKAKERLGGIRGAVLELISTEQKYETAIEI

ALGASAQHVVTDDEQSARKAIQYLKQNSFGRATFLPLSVIRDRQLQSRDAETAARHSSFL

GVASELVTFDPAYRSVIQNLLGTVLITEDLKGANELAKLLGHRYRIVTLEGDVVNPGGSM

TGGAVKKKNNSLLGRSRELEDVTKRLAEMEEKTALLEQEVKTLKHSIQDMEKKLADLRET

GEGLRLKQQDVKGQLYELQVAEKNINTHLELYDQEKSALSESDEERKVRKRKLEEELSAV

SEKMKQLEEDIDRLTKQKQTQSSTKESLSNELTELKIAAAKKEQACKGEEDNLARLKKEL

TETELALKEAKEDLSFLTSEMSSSTSGEEKLEEAAKHKLNDKTKTIELIALRRDQRIKLQ

HGLDTYERELKEMKRLYKQKTTLLKDEEVKLGRMEVELDNLLQYLREEYSLSFEGAKEKY

QLETDPEEARKRVKLIKLAIEELGTVNLGSIDEFERVNERYKFLSEQKEDLTEAKNTLFQ

VIEEMDEEMTKRFNDTFVQIRSHFDQVFRSLFGGGRAELRLTDPNDLLHSGVEIIAQPPG

KKLQNLNLLSGGERALTAIALLFSILKVRPVPFCVLDEVEAALDEANVFRFAQYLKKYSS

DTQFIVITHRKGTMEEADVLYGVTMQESGVSKVISVKLEETKEFVQ

>tr|Q7NBU0|Q7NBU0_MYCGA Chromosome partition protein Smc OS=Mycoplasma gallisepticum (strain R(low / passage 15 / clone 2)) GN=smc PE=3 SV=1

MLFLKKFHAQGFKSYADNISFTFDEHVTGIVGPNGSGKSNVVDALKWVLGERSMKNLRGK

TSDDVIFFGSQEKPASKFAEVSLTFDNSQGYLHDKRKEITVTRRVYRGSGVSEYLINNEP

SSLKEINDIFLDSGLTKGSLCIISQNTVSSFIEAKPEDRRQIFEDAAGIGRYAKKKQDAI

RQIARTNDNLKEITTIVNELNRDLKKLNQQAEKAILYAETKEKLKDLEITLSVNEYLISQ

KEIEALSEQIAEIDERLLKNDPQLQINQEKLEAFKKRYNSADQNVQKIQDELQKIYDEIV

LLEKRNVFNDLQLKSDLDSNDKNKKINALEQLLKSSEEQLKKYFELISTWEEELKEKDVD

KTDLANELENLKKSLATFQVKRYEANLQVQFYQNQKINQFAQDAGVRTVLNNKDAIGGVH

GIVQDFIKVEPEYELAISTALNKAAKNIIVDSNQDAINAVNFLKANKAGRATFLPLANLK

DRDVKPEHLEVLEQVEGYLGIAANLVNYHDQYDPAIRALLGQIIIASDLEAATKISKFTY

QLYRVISLGGDIVNAGGAITGGAESKQTHSLFNLDEKIDTLKNELLVAEKNINELNKKLE

FLTADYTKKDKEFNEQKIAIQRYQDLIVIEQKKLDDYKIQYEQLTDKTFDGKDVKWDDKK

IKDKLFSLETKKATLVQDLKINQEAKDMYQKQVNQLEKDVTLFYKEIDEDKNDKLKRREQ

LTKHENTIYLAKSKINESYNMAIEFAIENYNKPLPISLSQARSEVVKLQSTLNNLGAINM

EAIQELDIKKERYEKLYSQQQELINARERINQAIIRLDEKAIFEFDQLINNLNKELPKTF

YYLFGGGNCEIRYSNPEEKLTSGIEVFASPPGKNIGNLNLLSGGEKALVALSVLFSILKV

SSFPLVVLDEAESALDLANVERFANIIKNSSDQTQFLIITHREGTMVKCDKLIGATMQTK

GVTKMLSVSLHQAKDMAEEIESQ

sp|P51834|SMC_BACSU -MFLKRLDVIGFKSFAERISVDFVKGVTAVVGPNGSGKSNITDAIRWVLG 49

tr|Q7NBU0|Q7NBU0_MYCGA MLFLKKFHAQGFKSYADNISFTFDEHVTGIVGPNGSGKSNVVDALKWVLG 50

:***::.. ****:*:.**. * : **.:**********:.**::****

sp|P51834|SMC_BACSU EQSARSLRGGKMEDIIFAGSDSRKRLNLAEVTLTLDNDDHFLPIDFHEVS 99

tr|Q7NBU0|Q7NBU0_MYCGA ERSMKNLRGKTSDDVIFFGSQEKPASKFAEVSLTFDNSQGYLHDKRKEIT 100

*:* :.*** . :*:** **:.: ::***:**:**.: :* . :*::

sp|P51834|SMC_BACSU VTRRVYR-SGESEFLINNQPCRLKDIIDLFMDSGLGKEAFSIISQGKVEE 148

tr|Q7NBU0|Q7NBU0_MYCGA VTRRVYRGSGVSEYLINNEPSSLKEINDIFLDSGLTKGSLCIISQNTVSS 150

******* ** **:****:*. **:* *:*:**** * ::.****..*..

sp|P51834|SMC_BACSU ILSSKAEDRRSIFEEAAGVLKYKTRKKKAENKLFETQDNLNRVEDILHEL 198

tr|Q7NBU0|Q7NBU0_MYCGA FIEAKPEDRRQIFEDAAGIGRYAKKKQDAIRQIARTNDNLKEITTIVNEL 200

::.:*.****.***:***: :* .:*:.* .:: .*:***:.: *::**

sp|P51834|SMC_BACSU EGQVEPLKIQASIAKDYLEKKKELEHVEIALTAYDIEELHGKWSTLKEKV 248

tr|Q7NBU0|Q7NBU0_MYCGA NRDLKKLNQQAEKAILYAETKEKLKDLEITLSVNEYLISQKEIEALSEQI 250

: ::: *: **. * * *.*::*:.:**:*:. : : : .:*.*::

sp|P51834|SMC_BACSU QMAKEEELAESSAISAKEAKIEDTRDKIQALDESVDELQQVLLVTSEELE 298

tr|Q7NBU0|Q7NBU0_MYCGA AEIDERLLKNDPQLQINQEKLEAFKKRYNSADQNVQKIQDELQKIYDEIV 300

.*. * :.. :. :: *:* :.: :: *:.*:::*: * :*:

sp|P51834|SMC_BACSU KLEGRKEVLKERKKNAVQNQEQLEEAIVQFQQKETVLKEELSKQEAVFET 348

tr|Q7NBU0|Q7NBU0_MYCGA LLE-KRNVFNDLQLKSDLDSNDKNKKINALEQLLKSSEEQLKKYFELIST 349

** :::*::: : :: :.:: :: * ::* . :*:*.* ::.*

sp|P51834|SMC_BACSU LQAEVKQLRAQVKEKQQALSLHNENVEEKIEQLKSDYFELLNSQASIRNE 398

tr|Q7NBU0|Q7NBU0_MYCGA WEEELKEK-----------DVDKTDLANELENLKKSLATFQVKRYEANLQ 388

: *:*: .:.: :: :::*:**.. : .: . . :

sp|P51834|SMC_BACSU LQLLDDQMSQSAVTLQRLADNNEKHLQERHDISARKAACETEFARIEQEI 448

tr|Q7NBU0|Q7NBU0_MYCGA VQFYQNQKIN---------------------------------------- 398

:*: ::* :

sp|P51834|SMC_BACSU HSQVGAYRDMQTKYEQKKRQYEKNESALYQAYQYVQQARSKKDMLETMQG 498

tr|Q7NBU0|Q7NBU0_MYCGA --------------------------------QFAQDAG----------- 405

*:.*:*

sp|P51834|SMC_BACSU DFSGFYQGVKEVLKAKERLGGIRGAVLELISTEQKYETAIEIALGASAQH 548

tr|Q7NBU0|Q7NBU0_MYCGA --------VRTVLNNKDAIGGVHGIVQDFIKVEPEYELAISTALNKAAKN 447

*: **: *: :**::* * ::*..* :** **. **. :*::

sp|P51834|SMC_BACSU VVTDDEQSARKAIQYLKQNSFGRATFLPLSVIRDRQLQSRDAETAARHSS 598

tr|Q7NBU0|Q7NBU0_MYCGA IIVDSNQDAINAVNFLKANKAGRATFLPLANLKDRDVKPEHLEVLEQVEG 497

::.*.:*.* :*:::** *. ********: ::**:::... *. : ..

sp|P51834|SMC_BACSU FLGVASELVTFDPAYRSVIQNLLGTVLITEDLKGANELAKLLGHRYRIVT 648

tr|Q7NBU0|Q7NBU0_MYCGA YLGIAANLVNYHDQYDPAIRALLGQIIIASDLEAATKISKFTYQLYRVIS 547

:**:*::**.:. * ..*: *** ::*:.**:.*.:::*: : **:::

sp|P51834|SMC_BACSU LEGDVVNPGGSMTGGAVKKKNNSLLGRSRELEDVTKRLAEMEEKTALLEQ 698

tr|Q7NBU0|Q7NBU0_MYCGA LGGDIVNAGGAITGGAESKQTHSLFN---------------------LDE 576

* **:**.**::**** .*:.:**:. *::

sp|P51834|SMC_BACSU EVKTLKHSIQDMEKKLADLRETGEGLRLKQQDVKGQLYELQVAEKNINTH 748

tr|Q7NBU0|Q7NBU0_MYCGA KIDTLKN-------------------------------ELLVAEKNIN-- 593

::.***: ** *******

sp|P51834|SMC_BACSU LELYDQEKSALSESDEERKVRKRKLEEELSAVSEKMKQLEEDIDRLTKQK 798

tr|Q7NBU0|Q7NBU0_MYCGA --------------------------------------------ELNKKL 599

.*.*:

sp|P51834|SMC_BACSU QTQSSTKESLSNELTELKIAAAKKEQACKGEEDNLARLKKELTETELALK 848

tr|Q7NBU0|Q7NBU0_MYCGA EFLTADYTKKDKEFNEQKIAIQRYQDLIVIEQKKLDDYKIQY-------- 641

: :: . .:*:.* *** : :: *:.:* * :

sp|P51834|SMC_BACSU EAKEDLSFLTSEMSSSTSGEEKLEEAAKHKLNDKTKTIELIALRRDQRIK 898

tr|Q7NBU0|Q7NBU0_MYCGA ---EQLTDKTFDGKDVKWDDKKIKDKLFSLETKKATLVQDLKINQEAKDM 688

*:*: * : .. . .::*::: ..*:. :: : :.:: :

sp|P51834|SMC_BACSU LQHGLDTYERELKEMKRLYKQKTTLLKDEEVKLGRMEVELDNLLQYLREE 948

tr|Q7NBU0|Q7NBU0_MYCGA YQKQVNQLEKDVTLFYKEIDEDKNDKLKRREQLTKHENTIYLAKSKINES 738

*: :: *:::. : : .:... ... :* : * : . :.*.

sp|P51834|SMC_BACSU YSLSFEGAKEKY--QLETDPEEARKRVKLIKLAIEELGTVNLGSIDEFER 996

tr|Q7NBU0|Q7NBU0_MYCGA YNMAIEFAIENYNKPLPISLSQARSEVVKLQSTLNNLGAINMEAIQELDI 788

*.:::* * *:* * . .:**..* :: ::::**::*: :*:*::

sp|P51834|SMC_BACSU VNERYKFLSEQKEDLTEAKNTLFQVIEEMDEEMTKRFNDTFVQIRSHFDQ 1046

tr|Q7NBU0|Q7NBU0_MYCGA KKERYEKLYSQQQELINARERINQAIIRLDEKAIFEFDQLINNLNKELPK 838

:***: * .*:::* :*:: : *.* .:**: .*:: : ::...: :

sp|P51834|SMC_BACSU VFRSLFGGGRAELRLTDPNDLLHSGVEIIAQPPGKKLQNLNLLSGGERAL 1096

tr|Q7NBU0|Q7NBU0_MYCGA TFYYLFGGGNCEIRYSNPEEKLTSGIEVFASPPGKNIGNLNLLSGGEKAL 888

.* *****..*:* ::*:: * **:*::*.****:: *********:**

sp|P51834|SMC_BACSU TAIALLFSILKVRPVPFCVLDEVEAALDEANVFRFAQYLKKYSSDTQFIV 1146

tr|Q7NBU0|Q7NBU0_MYCGA VALSVLFSILKVSSFPLVVLDEAESALDLANVERFANIIKNSSDQTQFLI 938

.*:::******* ..*: ****.*:*** *** ***: :*: *.:***::

sp|P51834|SMC_BACSU ITHRKGTMEEADVLYGVTMQESGVSKVISVKLEETKEFVQ----- 1186

tr|Q7NBU0|Q7NBU0_MYCGA ITHREGTMVKCDKLIGATMQTKGVTKMLSVSLHQAKDMAEEIESQ 983

****:*** :.* * *.*** .**:*::**.*.::*::.:

>sp|P22523|MUKB_ECOLI Chromosome partition protein MukB OS=Escherichia coli (strain K12) GN=mukB PE=1 SV=2

MIERGKFRSLTLINWNGFFARTFDLDELVTTLSGGNGAGKSTTMAAFVTALIPDLTLLHF

RNTTEAGATSGSRDKGLHGKLKAGVCYSMLDTINSRHQRVVVGVRLQQVAGRDRKVDIKP

FAIQGLPMSVQPTQLVTETLNERQARVLPLNELKDKLEAMEGVQFKQFNSITDYHSLMFD

LGIIARRLRSASDRSKFYRLIEASLYGGISSAITRSLRDYLLPENSGVRKAFQDMEAALR

ENRMTLEAIRVTQSDRDLFKHLISEATNYVAADYMRHANERRVHLDKALEFRRELHTSRQ

QLAAEQYKHVDMARELAEHNGAEGDLEADYQAASDHLNLVQTALRQQEKIERYEADLDEL

QIRLEEQNEVVAEAIERQQENEARAEAAELEVDELKSQLADYQQALDVQQTRAIQYNQAI

AALNRAKELCHLPDLTADCAAEWLETFQAKELEATEKMLSLEQKMSMAQTAHSQFEQAYQ

LVVAINGPLARNEAWDVARELLREGVDQRHLAEQVQPLRMRLSELEQRLREQQEAERLLA

DFCKRQGKNFDIDELEALHQELEARIASLSDSVSNAREERMALRQEQEQLQSRIQSLMQR

APVWLAAQNSLNQLSEQCGEEFTSSQDVTEYLQQLLEREREAIVERDEVGARKNAVDEEI

ERLSQPGGSEDQRLNALAERFGGVLLSEIYDDVSLEDAPYFSALYGPSRHAIVVPDLSQV

TEHLEGLTDCPEDLYLIEGDPQSFDDSVFSVDELEKAVVVKIADRQWRYSRFPEVPLFGR

AARESRIESLHAEREVLSERFATLSFDVQKTQRLHQAFSRFIGSHLAVAFESDPEAEIRQ

LNSRRVELERALSNHENDNQQQRIQFEQAKEGVTALNRILPRLNLLADDSLADRVDEIRE

RLDEAQEAARFVQQFGNQLAKLEPIVSVLQSDPEQFEQLKEDYAYSQQMQRDARQQAFAL

TEVVQRRAHFSYSDSAEMLSGNSDLNEKLRERLEQAEAERTRAREALRGHAAQLSQYNQV

LASLKSSYDTKKELLNDLQRELQDIGVRADSGAEERARIRRDELHAQLSNNRSRRNQLEK

ALTFCEAEMDNLTRKLRKLERDYFEMREQVVTAKAGWCAVMRMVKDNGVERRLHRRELAY

LSADDLRSMSDKALGALRLAVADNEHLRDVLRMSEDPKRPERKIQFFVAVYQHLRERIRQ

DIIRTDDPVEAIEQMEIELSRLTEELTSREQKLAISSRSVANIIRKTIQREQNRIRMLNQ

GLQNVSFGQVNSVRLNVNVRETHAMLLDVLSEQHEQHQDLFNSNRLTFSEALAKLYQRLN

PQIDMGQRTPQTIGEELLDYRNYLEMEVEVNRGSDGWLRAESGALSTGEAIGTGMSILVM

VVQSWEDESRRLRGKDISPCRLLFLDEAARLDARSIATLFELCERLQMQLIIAAPENISP

EKGTTYKLVRKVFQNTEHVHVVGLRGFAPQLPETLPGTDEAPSQAS

sp|P51834|SMC_BACSU -MFLKRLDVIGFKSFAERIS--VDFVKGVTAVVGPNGSGKSNITDAIRWV 47

tr|Q7NBU0|Q7NBU0_MYCGA MLFLKKFHAQGFKSYADNIS--FTFDEHVTGIVGPNGSGKSNVVDALKWV 48

sp|P22523|MUKB_ECOLI MIERGKFRSLTLINWNGFFARTFDLDELVTTLSGGNGAGKSTTMAAFVTA 50

: :: : .: :: . : : ** : * **:***. *: .

sp|P51834|SMC_BACSU LGEQSARSLRGGKMEDIIFAGSDSR-----KRLNLAEVTLTLDNDDHFLP 92

tr|Q7NBU0|Q7NBU0_MYCGA LGERSMKNLRGKTSDDVIFFGSQEK-----PASKFAEVSLTFDNSQGYLH 93

sp|P22523|MUKB_ECOLI LIPDLTLLHFRNTTEAGATSGSRDKGLHGKLKAGVCYSMLDTINSRHQRV 100

* . : ** .: .. * *.

sp|P51834|SMC_BACSU IDFHEVSVTRRVYR-SGESEFLINNQPC---------------------- 119

tr|Q7NBU0|Q7NBU0_MYCGA DKRKEITVTRRVYRGSGVSEYLINNEPS---------------------- 121

sp|P22523|MUKB_ECOLI VVGVRLQQVAGRDRKVDIKPFAIQGLPMSVQPTQLVTETLNERQARVLPL 150

.: . * . . : *:. *

sp|P51834|SMC_BACSU -------------------RLKDIIDLFMDSGLGKEAFSIISQGK----- 145

tr|Q7NBU0|Q7NBU0_MYCGA -------------------SLKEINDIFLDSGLTKGSLCIISQNT----- 147

sp|P22523|MUKB_ECOLI NELKDKLEAMEGVQFKQFNSITDYHSLMFDLGIIARRLRSASDRSKFYRL 200

:.: .:::* *: : *: .

sp|P51834|SMC_BACSU ----------------VEEILSSKAEDRRSIFEEAAGVLKYKTRKKKAEN 179

tr|Q7NBU0|Q7NBU0_MYCGA ----------------VSSFIEAKPEDRRQIFEDAAGIGRYAKKKQDAIR 181

sp|P22523|MUKB_ECOLI IEASLYGGISSAITRSLRDYLLPENSGVRKAFQDMEAALRENRMTLEAIR 250

: . : .: .. *. *:: . : . .* .

sp|P51834|SMC_BACSU KLFETQDNLNRVEDILH--------------------------------- 196

tr|Q7NBU0|Q7NBU0_MYCGA QIARTNDNLKEITTIVN--------------------------------- 198

sp|P22523|MUKB_ECOLI VTQSDRDLFKHLISEATNYVAADYMRHANERRVHLDKALEFRRELHTSRQ 300

.* ::.:

sp|P51834|SMC_BACSU ----------ELEGQVEPLK-IQASIAKDYLEKKKELEHVEIALTAY--- 232

tr|Q7NBU0|Q7NBU0_MYCGA ----------ELNRDLKKLN-QQAEKAILYAETKEKLKDLEITLSVN--- 234

sp|P22523|MUKB_ECOLI QLAAEQYKHVDMARELAEHNGAEGDLEADYQAASDHLNLVQTALRQQEKI 350

:: :: : :.. * ...*: :: :*

sp|P51834|SMC_BACSU -----DIEELHGKWSTLKEKVQMAKEEELAESSAISAKEAKIEDTRDKIQ 277

tr|Q7NBU0|Q7NBU0_MYCGA -----EYLISQKEIEALSEQIAEIDERLLKNDPQLQINQEKLEAFKKRYN 279

sp|P22523|MUKB_ECOLI ERYEADLDELQIRLEEQNEVVAEAIERQQENEARAEAAELEVDELKSQLA 400

: : . . .* : *. :.. . : ::: :.:

sp|P51834|SMC_BACSU ALDESVDELQQVLLVTSEELEKLEGRKEVLKERKKNAVQNQEQLE----- 322

tr|Q7NBU0|Q7NBU0_MYCGA SADQNVQKIQDELQKIYDEIVLLE-KRNVFNDLQLKSDLDSNDKN----- 323

sp|P22523|MUKB_ECOLI DYQQALDVQQTRAIQYNQAIAALNRAKELCHLPDLTADCAAEWLETFQAK 450

:: :: * : : *: ::: : . .: : :

sp|P51834|SMC_BACSU -----EAIVQFQQKETVLKEELSKQEAVFETLQAEVKQLRA----QVKEK 363

tr|Q7NBU0|Q7NBU0_MYCGA -----KKINALEQLLKSSEEQLKKYFELISTWEEELKEK----------- 357

sp|P22523|MUKB_ECOLI ELEATEKMLSLEQKMSMAQTAHSQFEQAYQLVVAINGPLARNEAWDVARE 500

: : ::* . : .: .

sp|P51834|SMC_BACSU QQALSLHNENVEEKIEQLKSDYFELLNSQASIRNELQLLDDQMSQSAVTL 413

tr|Q7NBU0|Q7NBU0_MYCGA ----DVDKTDLANELENLKKSLATFQVKRYEANLQVQFYQNQKIN----- 398

sp|P22523|MUKB_ECOLI LLREGVDQRHLAEQVQPLRMRLSELEQRLREQQEAERLLADFCKR----- 545

.:.: .: :::: *: : . . :: : .

sp|P51834|SMC_BACSU QRLADNNEKHLQERHDISARKAACETEFARIEQEIHSQVGAYRDMQTKYE 463

tr|Q7NBU0|Q7NBU0_MYCGA --------------------------------------------------

sp|P22523|MUKB_ECOLI QGKNFDIDELEALHQELEARIASLSDSVSNAREERMALRQEQEQLQSRIQ 595

sp|P51834|SMC_BACSU QKKRQYEKNESALYQAYQYVQQARSKKDMLETMQGDFSGFYQGVKEVLKA 513

tr|Q7NBU0|Q7NBU0_MYCGA -----------------QFAQDAG-------------------VRTVLNN 412

sp|P22523|MUKB_ECOLI SLMQRAPVWLAAQNSLNQLSEQCGEEFTSSQDVTEYLQQLLEREREAIVE 645

* ::. : .:

sp|P51834|SMC_BACSU KERLGGIRGAVLELIS-------------------------------TEQ 532

tr|Q7NBU0|Q7NBU0_MYCGA KDAIGGVHGIVQDFIK-------------------------------VEP 431

sp|P22523|MUKB_ECOLI RDEVGARKNAVDEEIERLSQPGGSEDQRLNALAERFGGVLLSEIYDDVSL 695

:: :*. :. * : *. ..

sp|P51834|SMC_BACSU KYETAIEIALGASAQHVVTDDEQSARKAIQ-------------------- 562

tr|Q7NBU0|Q7NBU0_MYCGA EYELAISTALNKAAKNIIVDSNQDAINAVN-------------------- 461

sp|P22523|MUKB_ECOLI EDAPYFSALYGPSRHAIVVPDLSQVTEHLEGLTDCPEDLYLIEGDPQSFD 745

: :. . : : ::. . ... : ::

sp|P51834|SMC_BACSU --YLKQNSFGRATFLPLSV-----------------IRDRQLQSRDAETA 593

tr|Q7NBU0|Q7NBU0_MYCGA --FLKANKAGRATFLPLAN-----------------LKDRDVKPEHLEVL 492

sp|P22523|MUKB_ECOLI DSVFSVDELEKAVVVKIADRQWRYSRFPEVPLFGRAARESRIESLHAERE 795

:. :. :*..: :: :: ::. . *

sp|P51834|SMC_BACSU ARHSSFLGVASELVTFDPAYRSVIQNLLGTVLITEDLKGANELAKLLGHR 643

tr|Q7NBU0|Q7NBU0_MYCGA EQVEGYLGIAANLVNYHDQYDPAIRALLGQIIIASDLEAATKISKFTYQL 542

sp|P22523|MUKB_ECOLI VLSERFATLSFDVQKTQRLHQAFSRFIGSHLAVAFESDPEAEIRQLNSRR 845

. : :: :: . . : . : : . : :: : . :: :: :

sp|P51834|SMC_BACSU YRIVTLEGDVVNPGGS------------------------MTGGAVKKKN 669

tr|Q7NBU0|Q7NBU0_MYCGA YRVISLGGDIVNAGGA------------------------ITGGAESKQT 568

sp|P22523|MUKB_ECOLI VELERALSNHENDNQQQRIQFEQAKEGVTALNRILPRLNLLADDSLADRV 895

.: .: * . ::..: .:

sp|P51834|SMC_BACSU NSLLGRSRELEDVTKRLAEMEEKTALLEQEVKTLKHSIQDMEKKLADLRE 719

tr|Q7NBU0|Q7NBU0_MYCGA HSLFN---------------------LDEKIDTLKN-------------- 583

sp|P22523|MUKB_ECOLI DEIRERLDEAQEAARFVQQFGNQLAKLEPIVSVLQSDPEQFEQLKEDYAY 945

..: *: :..*:

sp|P51834|SMC_BACSU TGEGLRLKQQDVKGQLYELQVAEKNINTHLELYDQEKSALSESDEERKVR 769

tr|Q7NBU0|Q7NBU0_MYCGA -----------------ELLVAEKNIN----------------------- 593

sp|P22523|MUKB_ECOLI S---QQMQRDARQQAFALTEVVQRRAHFSYSDSAEMLSGNSDLNEKLRER 992

*.::. :

sp|P51834|SMC_BACSU KRKLEEELSAVSEKMKQLEEDIDRLTKQKQTQSSTKESLSNELTELK--- 816

tr|Q7NBU0|Q7NBU0_MYCGA -----------------------ELNKKLEFLTADYTKKDKEFNEQK--- 617

sp|P22523|MUKB_ECOLI LEQAEAERTRAREALRGHAAQLSQYNQVLASLKSSYDTKKELLNDLQREL 1042

. .: .: . .: :.: :

sp|P51834|SMC_BACSU --IAAAKKEQACKGEEDNLARLKKELTETELALKEAKEDLSFLTSEMSSS 864

tr|Q7NBU0|Q7NBU0_MYCGA --IAIQRYQDLIVIEQKKLDDYKIQY-----------EQLTDKTFDGKDV 654

sp|P22523|MUKB_ECOLI QDIGVRADSGAEERARIRRDELHAQLSNNRSRRNQLEKALTFCEAEMDNL 1092

*. . . . : : : *: : ..

sp|P51834|SMC_BACSU TSGEEKLEEAAKHKLNDKTKTIELIALRRDQRIKLQHGLDTYERELKEMK 914

tr|Q7NBU0|Q7NBU0_MYCGA KWDDKKIKDKLFSLETKKATLVQDLKINQEAKDMYQKQVNQLEKDVTLFY 704

sp|P22523|MUKB_ECOLI TRKLRKLERDYFEMREQVVTAKAGWCAVMRMVKDNGVERRLHRRELAYLS 1142

. .*:: . .. .::: :

sp|P51834|SMC_BACSU RLYKQKTTLLKDEEVKLGRMEVELDNLLQYLREEYSLSFEGAKEKY--QL 962

tr|Q7NBU0|Q7NBU0_MYCGA KEIDEDKNDKLKRREQLTKHENTIYLAKSKINESYNMAIEFAIENYNKPL 754

sp|P22523|MUKB_ECOLI ADDLRSMSDKALGALRLAVADNEHLRDVLRMSEDP-KRPERKIQFFVAVY 1191

.. . :* : : *. * : :

sp|P51834|SMC_BACSU ETDPEEARKRVKLIKLAIEELGTVNLGSIDEFERVNERYKFLSEQKE--- 1009

tr|Q7NBU0|Q7NBU0_MYCGA PISLSQARSEVVKLQSTLNNLGAINMEAIQELDIKKERYEKLYSQQQ--- 801

sp|P22523|MUKB_ECOLI QHLRERIRQDIIRTDDPVEAIEQMEIELSRLTEELTSREQKLAISSRSVA 1241

.. *. : . .:: : ::: : ..* : * ...

sp|P51834|SMC_BACSU ------------------------------------DLTEAKNTLFQVIE 1023

tr|Q7NBU0|Q7NBU0_MYCGA ------------------------------------ELINARERINQAII 815

sp|P22523|MUKB_ECOLI NIIRKTIQREQNRIRMLNQGLQNVSFGQVNSVRLNVNVRETHAMLLDVLS 1291

:: ::: : :.:

sp|P51834|SMC_BACSU EMDEEMTKRFNDTFVQIRSHFDQVFRSLFGGGRAELR--------LTDPN 1065

tr|Q7NBU0|Q7NBU0_MYCGA RLDEKAIFEFDQLINNLNKELPKTFYYLFGGGNCEIR--------YSNPE 857

sp|P22523|MUKB_ECOLI EQHEQHQDLFNSNRLTFSEALAKLYQRLNPQIDMGQRTPQTIGEELLDYR 1341

. .*: *:. : . : : : * * : .

sp|P51834|SMC_BACSU DLLHSGVEIIAQPPGKKLQNLNLLSGGERALTAIALLFSILK-------- 1107

tr|Q7NBU0|Q7NBU0_MYCGA EKLTSGIEVFASPPGKNIGNLNLLSGGEKALVALSVLFSILK-------- 899

sp|P22523|MUKB_ECOLI NYLEMEVEVNRGSDGWLRAESGALSTGEAIGTGMSILVMVVQSWEDESRR 1391

: * :*: . * : . ** ** ..:::*. :::

sp|P51834|SMC_BACSU -----VRPVPFCVLDEVE--AALDEANVFRFAQYLKKYSSDTQFIVITHR 1150

tr|Q7NBU0|Q7NBU0_MYCGA -----VSSFPLVVLDEAE--SALDLANVERFANIIKNSSDQTQFLIITHR 942

sp|P22523|MUKB_ECOLI LRGKDISPCRLLFLDEAARLDARSIATLFELCERLQMQLIIAAPENISPE 1441

: . : .***. * . *.: .:.: :: : *: .

sp|P51834|SMC_BACSU KGTMEEADVLYGVTMQESGVSKVISVKLEETKEFVQ--------- 1186

tr|Q7NBU0|Q7NBU0_MYCGA EGTMVKCDKLIGATMQTKGVTKMLSVSLHQAKDMAEEIESQ---- 983

sp|P22523|MUKB_ECOLI KGTTYKLVRKVFQNTEHVHVVGLRGFAPQLPETLPGTDEAPSQAS 1486

:** : . : * : .. . .: :

1. **Recombination protein RecR (gene name *recR*)**

>sp|P0A7H6|RECR_ECOLI Recombination protein RecR OS=Escherichia coli (strain K12) GN=recR PE=3 SV=1

MQTSPLLTQLMEALRCLPGVGPKSAQRMAFTLLQRDRSGGMRLAQALTRAMSEIGHCADC

RTFTEQEVCNICSNPRRQENGQICVVESPADIYAIEQTGQFSGRYFVLMGHLSPLDGIGP

DDIGLDRLEQRLAEEKITEVILATNPTVEGEATANYIAELCAQYDVEASRIAHGVPVGGE

LEMVDGTTLSHSLAGRHKIRF

>sp|P24277|RECR_BACSU Recombination protein RecR OS=Bacillus subtilis (strain 168) GN=recR PE=3 SV=2

MQYPEPISKLIDSFMKLPGIGPKTAVRLAFFVLGMKEDVVLDFAKALVNAKRNLTYCSVC

GHITDQDPCYICEDTRRDKSVICVVQDPKDVIAMEKMKEYNGQYHVLHGAISPMDGIGPE

DIKIPELLKRLQDDQVTEVILATNPNIEGEATAMYISRLLKPSGIKLSRIAHGLPVGGDL

EYADEVTLSKALEGRREL

>tr|Q7NAN7|Q7NAN7_MYCGA Recombination protein RecR OS=Mycoplasma gallisepticum (strain R(low / passage 15 / clone 2)) GN=recR PE=3 SV=2

MTSDLDLNEFNNLVEQISDLPSVSKKQAKKITQYLMTKSDRYVYDLIDVLKRAKLSIKIC

EMCQGWSNRSICSICSDESRNNNELCIVSFFDDLNVIEESQAYHGKYFILNHEISKKNKR

IIEEINFDLLLDLIKKQKIEKVIIATNFTQDGQTTANYLRFLLDDFDLKIYRLGMGLPYN

SSIDYADSFSLKGAFENKQLIKDKKA

sp|P0A7H6|RECR_ECOLI ---MQTSPLLTQLMEALRCLPGVGPKSAQRMAFTLLQRDRSGGMRLAQAL 47

sp|P24277|RECR_BACSU ---MQYPEPISKLIDSFMKLPGIGPKTAVRLAFFVLGMKEDVVLDFAKAL 47

tr|Q7NAN7|Q7NAN7_MYCGA MTSDLDLNEFNNLVEQISDLPSVSKKQAKKITQYLMTKSDRYVYDLIDVL 50

:.:*:: : **.:. * * ::: :: . : ..*

sp|P0A7H6|RECR_ECOLI TRAMSEIGHCADCRTFTEQEVCNICSNPRRQENGQICVVESPADIYAIEQ 97

sp|P24277|RECR_BACSU VNAKRNLTYCSVCGHITDQDPCYICEDTRR-DKSVICVVQDPKDVIAMEK 96

tr|Q7NAN7|Q7NAN7_MYCGA KRAKLSIKICEMCQGWSNRSICSICSDESR-NNNELCIVSFFDDLNVIEE 99

.* .: * * :::. * **.: * ::. :*:*. *: .:*:

sp|P0A7H6|RECR_ECOLI TGQFSGRYFVLMGHLSPLDGIGPDDIGLDRLEQRLAEEKITEVILATNPT 147

sp|P24277|RECR_BACSU MKEYNGQYHVLHGAISPMDGIGPEDIKIPELLKRLQDDQVTEVILATNPN 146

tr|Q7NAN7|Q7NAN7_MYCGA SQAYHGKYFILNHEISKKNKRIIEEINFDLLLDLIKKQKIEKVIIATNFT 149

: *:*.:* :* : ::* : * . : .::: :**:*** .

sp|P0A7H6|RECR_ECOLI VEGEATANYIAELCAQYDVEASRIAHGVPVGGELEMVDGTTLSHSLAGRH 197

sp|P24277|RECR_BACSU IEGEATAMYISRLLKPSGIKLSRIAHGLPVGGDLEYADEVTLSKALEGRR 196

tr|Q7NAN7|Q7NAN7_MYCGA QDGQTTANYLRFLLDDFDLKIYRLGMGLPYNSSIDYADSFSLKGAFENKQ 199

:*::** *: * .:: *:. *:* ...:: .* :*. :: .::

sp|P0A7H6|RECR_ECOLI KIRF--- 201

sp|P24277|RECR_BACSU EL----- 198

tr|Q7NAN7|Q7NAN7_MYCGA LIKDKKA 206

:

1. **Recombination protein RecO (gene name *MGA_0016*)**

>sp|P0A7H3|RECO_ECOLI DNA repair protein RecO OS=Escherichia coli (strain K12) GN=recO PE=1 SV=1

MEGWQRAFVLHSRPWSETSLMLDVFTEESGRVRLVAKGARSKRSTLKGALQPFTPLLLRF

GGRGEVKTLRSAEAVSLALPLSGITLYSGLYINELLSRVLEYETRFSELFFDYLHCIQSL

AGVTGTPEPALRRFELALLGHLGYGVNFTHCAGSGEPVDDTMTYRYREEKGFIASVVIDN

KTFTGRQLKALNAREFPDADTLRAAKRFTRMALKPYLGGKPLKSRELFRQFMPKRTVKTH

YE

>sp|P42095|RECO_BACSU DNA repair protein RecO OS=Bacillus subtilis (strain 168) GN=recO PE=1 SV=3

MLTKCEGIVLRTNDYGETNKIVTLLTREHGKIGVMARGAKKPNSRLSAVSQPFLYGSFLM

QKTSGLGTLQQGEMILSMRGIREDLFLTAYAAYVAELVDRGTEEKKPNPYLFEFILESLK

QLNEGTDPDVITFIVQMKMLGVMGLYPELNHCVHCKSQDGTFHFSVRDNGFICHRCFEKD

PYRIPIKPQTARLLRLFYYFDLSRLGNVSLKEETKAELKQVIDLYYEEYSGLYLKSKRFL

DQMESMKHLMGENKS

>tr|Q7NB87|Q7NB87_MYCGA Putative recombinational DNA repair protein RecO OS=Mycoplasma gallisepticum (strain R(low / passage 15 / clone 2)) GN=MYCGA3920 PE=4 SV=1

MSSITKKGYIIDYFDHNENDQIIKILFDDNTLTSLISVGSKKILSKNGRYITLGSLHDFE

FFQARSIERLSKLKKIHEIDTKDATISESLPMVIMHYYLNKKSGELENNFFNFYDDVINY

VIKQRYSDETIIIYILLNIINLEGIAFQLLNCGICNSKQVITLSFKKMYGLCEKCAYEQH

EFLYDKNFMRNIFWLIYKNDYEVSTLESRKYISLIKGLASAIYHNAGIYLEPVFSYLIKL

K

sp|P42095|RECO_BACSU -MLTKCEGIVLRTNDYGETNKIVTLLTREHGKIGVMARGAKKPNSRLSAV 49

sp|P0A7H3|RECO_ECOLI -MEGWQRAFVLHSRPWSETSLMLDVFTEESGRVRLVAKGARSKRSTLKGA 49

tr|Q7NB87|Q7NB87_MYCGA MSSITKKGYIIDYFDHNENDQIIKILFDDNTLTSLISVGSKKILSKNGRY 50

.. :: .*.. :: :: : ::: *::. *

sp|P42095|RECO_BACSU SQPFLYGSFLMQKTSGLGTLQQGEMILSMRGIREDLFLTAYAAYVAELVD 99

sp|P0A7H3|RECO_ECOLI LQPFTPLLLRFGGRGEVKTLRSAEAVSLALPLSGITLYSG--LYINELLS 97

tr|Q7NB87|Q7NB87_MYCGA ITLGSLHDFEFFQARSIERLSKLKKIHEIDTKDATISESLPMVIMHYYLN 100

: : : * . : : : : :.

sp|P42095|RECO_BACSU RGTEEKKPNPYLFEFILESLKQLNEGT-DPDVITFIVQMKMLGVMGLYPE 148

sp|P0A7H3|RECO_ECOLI RVLEYETRFSELFFDYLHCIQSLAGVTGTPEPALRRFELALLGHLGYGVN 147

tr|Q7NB87|Q7NB87_MYCGA KKSGELENN--FFNFYDDVINYVIKQRYSDETIIIYILLNIINLEGIAFQ 148

: :* . :: : : . : ::. * :

sp|P42095|RECO_BACSU LNHCVHCK-SQDGTFHFSVRD-NGFICHRCFEKDPYRIPIKPQTARLLRL 196

sp|P0A7H3|RECO_ECOLI FTHCAGSGEPVDDTMTYRYREEKGFIASVVIDN-------KTFTGRQLKA 190

tr|Q7NB87|Q7NB87_MYCGA LLNCGICN--SKQVITLSFKKMYGLCEKCAYEQHEF-----LYDKNFMRN 191

: :* . . .: :. *: :: . ::

sp|P42095|RECO_BACSU FYYFDLSRLGNVSLKEETKAELKQVIDLYYEEYSG-LYLKSKRFLDQMES 245

sp|P0A7H3|RECO_ECOLI LNAREFP-------DADTLRAAKRFTRMALKPYLGGKPLKSRELFRQFMP 233

tr|Q7NB87|Q7NB87_MYCGA IFWLIYKN-----DYEVSTLESRKYISLIKGLASA-IYHNAGIYLEPVFS 235

: : :: : . :: : . .

sp|P42095|RECO_BACSU MKHLMGENKS 255

sp|P0A7H3|RECO_ECOLI KRTVKTHYE- 242

tr|Q7NB87|Q7NB87_MYCGA YLIKLK---- 241

1. **Holliday junction resolvase (gene name *recU*)**

Gene is absent in *E. coli* (strain K12) genome.

>sp|P39792|RECU_BACSU Holliday junction resolvase RecU OS=Bacillus subtilis (strain 168) GN=recU PE=1 SV=1

MIRYPNGKTFQPKHSVSSQNSQKRAPSYSNRGMTLEDDLNETNKYYLTNQIAVIHKKPTP

VQIVNVHYPKRSAAVIKEAYFKQSSTTDYNGIYKGRYIDFEAKETKNKTSFPLQNFHDHQ

IEHMKQVKAQDGICFVIISAFDQVYFLEADKLFYFWDRKEKNGRKSIRKDELEETAYPIS

LGYAPRIDYISIIEQLYFSPSSGAKG

>tr|Q7NBW8|Q7NBW8_MYCGA Holliday junction resolvase RecU OS=Mycoplasma gallisepticum (strain R(low / passage 15 / clone 2)) GN=recU PE=3 SV=2

MHQNRGMFLETLINNTIKHNELAHKGLIFKRHLPINVYNFANRRVTGWLKEKTQTDYYGL

YKGYFFDFDAKQSSKINYSLKNIKQHQLDHLRKIHEQGGIAFILLLIVPKEEFYMIPIKK

IDSWLKNQESNTLKYEWIQKSSFKLELFYPGVIGIFEALQEWIDLITLRRSSGS

sp|P39792|RECU_BACSU MIRYPNGKTFQPKHSVSSQNSQKRAPSYSNRGMTLEDDLNETNKYYLTNQ 50

tr|Q7NBW8|Q7NBW8_MYCGA --------------------------MHQNRGMFLETLINNTIKHNELAH 24

:.**** ** :*:* *: :

sp|P39792|RECU_BACSU IAVIHKKPTPVQIVNVHYPKRSAAVIKEAYFKQSSTTDYNGIYKGRYIDF 100

tr|Q7NBW8|Q7NBW8_MYCGA KGLIFKRHLPINVYNFANRR------VTGWLKEKTQTDYYGLYKGYFFDF 68

.:*.*: *::: *. : .::*:.: *** *:*** ::**

sp|P39792|RECU_BACSU EAKETKNKTSFPLQNFHDHQIEHMKQVKAQDGICFVIISAF--DQVYFLE 148

tr|Q7NBW8|Q7NBW8_MYCGA DAKQSS-KINYSLKNIKQHQLDHLRKIHEQGGIAFILLLIVPKEEFYMIP 117

:**::. * .:.*:*:::**::*::::: *.**.*::: . ::.*::

sp|P39792|RECU_BACSU ADKLFYFWDRKEKNGRKSIRKDELEETAYPISLGYAPRIDYIS----IIE 194

tr|Q7NBW8|Q7NBW8_MYCGA IKKIDSWLKNQESN---TLKYEWIQKSSFKLELFYPGVIGIFEALQEWID 164

.*: : ..:*.* ::: : :::::: :.* *. *. :. *:

sp|P39792|RECU_BACSU QLYFSPSSGAKG 206

tr|Q7NBW8|Q7NBW8_MYCGA LITLRRSSGS-- 174

: : ***:

>sp|P0A814|RUVC_ECOLI Crossover junction endodeoxyribonuclease RuvC OS=Escherichia coli (strain K12) GN=ruvC PE=1 SV=2

MAIILGIDPGSRVTGYGVIRQVGRQLSYLGSGCIRTKVDDLPSRLKLIYAGVTEIITQFQ

PDYFAIEQVFMAKNADSALKLGQARGVAIVAAVNQELPVFEYAARQVKQTVVGIGSAEKS

QVQHMVRTLLKLPANPQADAADALAIAITHCHVSQNAMQMSESRLNLARGRLR

sp|P39792|RECU_BACSU MIRYPNGKTFQPKHSVSSQNSQKRAPSYSNRGMTLEDDLN-ETNKYYLTN 49

tr|Q7NBW8|Q7NBW8_MYCGA --------------------------MHQNRGMFLETLIN-NTIKHNELA 23

sp|P0A814|RUVC_ECOLI --------------------------MAIILGIDPGSRVTGYGVIRQVGR 24

*: :.

sp|P39792|RECU_BACSU QIAVIHKKPTPVQIVNVHYPKRSAAVIKEAYFKQSSTTDYNGIYKGRYID 99

tr|Q7NBW8|Q7NBW8_MYCGA HKGLIFKRHLPINVYNFANRR------VTGWLKEKTQTDYYGLYKGYFFD 67

sp|P0A814|RUVC_ECOLI QLSYLGSGCIRTKVDDLPSRLK---------LIYAGVTEIITQFQPDYFA 65

: . : . :: :. : *: :: ::

sp|P39792|RECU_BACSU FEAKETKNKTSFPLQNFHDHQIEHMKQVKAQDGICFVIISAF--DQVYFL 147

tr|Q7NBW8|Q7NBW8_MYCGA FDAKQSS-KINYSLKNIKQHQLDHLRKIHEQGGIAFILLLIVPKEEFYMI 116

sp|P0A814|RUVC_ECOLI IEQVFMAKNADSALKLGQARGVAIVAAVNQELPVFEYAARQVKQTVVGIG 115

:: : . .*: : : : : :: : : . . :

sp|P39792|RECU_BACSU EADKLFYFWDRKEKNGRKSIRKDELEETAYPISLGYAPRIDYIS----II 193

tr|Q7NBW8|Q7NBW8_MYCGA PIKKIDSWLKNQESN---TLKYEWIQKSSFKLELFYPGVIGIFEALQEWI 163

sp|P0A814|RUVC_ECOLI SAEKSQ--VQHMVRT---LLKLPANPQADAADALAIAITHCHVSQNAMQM 160

.* .. . :: :: * . .. :

sp|P39792|RECU_BACSU EQLYFSPSSGAKG 206

tr|Q7NBW8|Q7NBW8_MYCGA DLITLRRSSGS-- 174

sp|P0A814|RUVC_ECOLI SESRLNLARGRLR 173

. : : *

1. **Holliday junction resolvase (gene name *MGA_0836*)**

>sp|P0A8I1|RUVX_ECOLI Putative Holliday junction resolvase OS=Escherichia coli (strain K12) GN=yqgF PE=1 SV=1

MSGTLLAFDFGTKSIGVAVGQRITGTARPLPAIKAQDGTPDWNIIERLLKEWQPDEIIVG

LPLNMDGTEQPLTARARKFANRIHGRFGVEVKLHDERLSTVEARSGLFEQGGYRALNKGK

VDSASAVIILESYFEQGY

>sp|O34634|RUVX_BACSU Putative Holliday junction resolvase OS=Bacillus subtilis (strain 168) GN=yrrK PE=1 SV=1

MRILGLDLGTKTLGVALSDEMGWTAQGIETIKINEAEGDYGLSRLSELIKDYTIDKIVLG

FPKNMNGTVGPRGEASQTFAKVLETTYNVPVVLWDERLTTMAAEKMLIAADVSRQKRKKV

IDKMAAVMILQGYLDSLN

>sp|Q7NBY7|RUVX_MYCGA Putative Holliday junction resolvase OS=Mycoplasma gallisepticum (strain R(low / passage 15 / clone 2)) GN=MYCGA1230 PE=3 SV=1

MYYVALDVGSRTLGIATGDGEFKIASPYCVISFNQYDFRQCLAELKEKTASFFYDFKFVI

GMPKNIDQTKSSTTEMVENFIELLKANYKNEVIIYDESYTSIIADQLLIDNQIKAKKRKE

KIDKLAAFVILQSFFDDDRYPK

sp|O34634|RUVX_BACSU --MRILGLDLGTKTLGVALSDEMGWTAQGIETIKINEAEGDYGLSRLSELIKDYTID-KI 57

sp|Q7NBY7|RUVX_MYCGA --MYYVALDVGSRTLGIATGDGEFKIASPYCVISFNQYDFRQCLAELKEKTASFFYDFKF 58

sp|P0A8I1|RUVX_ECOLI MSGTLLAFDFGTKSIGVAVGQRITGTARPLPAIKAQDGTPDWNIIER--LLKEWQPD-EI 57

:.:*.*::::*:* .: * .*. :: : . .: * ::

sp|O34634|RUVX_BACSU VLGFPKNMNGTVGPRGEASQTFAKVLETTYNVPVVLWDERLTTMAAEKMLIAADVSRQKR 117

sp|Q7NBY7|RUVX_MYCGA VIGMPKNIDQTKSSTTEMVENFIELLKANYKNEVIIYDESYTSIIADQLLIDNQIKAKKR 118

sp|P0A8I1|RUVX_ECOLI IVGLPLNMDGTEQPLTARARKFANRIHGRFGVEVKLHDERLSTVEARSGLFEQGGYRALN 117

::*:* *:: * . ..* : :. : * : ** ::: * . *: .

sp|O34634|RUVX_BACSU KKVIDKMAAVMILQGYLDSLN--- 138

sp|Q7NBY7|RUVX_MYCGA KEKIDKLAAFVILQSFFDDDRYPK 142

sp|P0A8I1|RUVX_ECOLI KGKVDSASAVIILESYFEQGY--- 138

* :*. :*.:**:.:::.

**21. DNA-polymerase IV (gene name *dinB*)**

>sp|Q47155|DPO4_ECOLI DNA polymerase IV OS=Escherichia coli (strain K12) GN=dinB PE=1 SV=1

MRKIIHVDMDCFFAAVEMRDNPALRDIPIAIGGSRERRGVISTANYPARKFGVRSAMPTG

MALKLCPHLTLLPGRFDAYKEASNHIREIFSRYTSRIEPLSLDEAYLDVTDSVHCHGSAT

LIAQEIRQTIFNELQLTASAGVAPVKFLAKIASDMNKPNGQFVITPAEVPAFLQTLPLAK

IPGVGKVSAAKLEAMGLRTCGDVQKCDLVMLLKRFGKFGRILWERSQGIDERDVNSERLR

KSVGVERTMAEDIHHWSECEAIIERLYPELERRLAKVKPDLLIARQGVKLKFDDFQQTTQ

EHVWPRLNKADLIATARKTWDERRGGRGVRLVGLHVTLLDPQMERQLVLGL

>sp|Q02886|DINB_BACSU Protein DinB OS=Bacillus subtilis (strain 168) GN=dinB PE=3 SV=2

MSDFAFKLYEYNVWANQQIFNRLKELPKEIYHQEIQSVFPSISHVLSHVYLSDLGWIEVF

SGKTLSDALALAEQLKEQTEAKEIEEMEDLFLRLSERYILFLQQKEQLNKPLQIQNPSSG

IMKTTVSELLPHVVNHGTYHRGNITAMLRQAGYASAPTDYGLYLFMTKTEKA

>tr|Q7NAR2|Q7NAR2_MYCGA DNA polymerase IV OS=Mycoplasma gallisepticum (strain R(low / passage 15 / clone 2)) GN=dinB PE=3 SV=2

MFLMDRKTIFHIDFDAFFASVEENFNPEYNNKPLVVGSKSNGSIVSSANYIARKFGVRSA

MPIFQAKKLCPSLIIAEVDYPKYERVSAYVFSYLRDFVSNKMEVASIDECYMDVTDILEK

NSEISASDLAKKIQKQIYELTQLTVSIGISSNLFLAKMASDQNKPNGVYEIWPDEIEEKL

WPLEIKKMYLIGSKKIPLLNQLNIKNIGNFAQFENKELLIDIFKNMYWDHYNHAHGKGSD

FVDYERNSRKSISVSKNIRHKVSDYESLLKLFNDLFDDVYSRLKNHNLLAKNISVSIRTE

KVRSLSFSFKQYSDKKTLFYNKAIDLFERLHNNQLVANISISFGNITNKYKFMPNINIYE

ELSKEQKDTMLLKKIVNQINKEYNKELVNIADDFDYFKFQK

sp|Q47155|DPO4_ECOLI -----MRKIIHVDMDCFFAAVEMRDNPALRDIPIAIGGSRERRGVISTAN 45

tr|Q7NAR2|Q7NAR2_MYCGA MFLMDRKTIFHIDFDAFFASVEENFNPEYNNKPLVVG-SKSNGSIVSSAN 49

sp|Q02886|DINB_BACSU ---------------------------------------------MSDFA 5

:*

sp|Q47155|DPO4_ECOLI YPARKFGVRSAMPTGMALKLCPHLTLLPGRFDAYKEASNHIREIFSRYTS 95

tr|Q7NAR2|Q7NAR2_MYCGA YIARKFGVRSAMPIFQAKKLCPSLIIAEVDYPKYERVSAYVFSYLRDFVS 99

sp|Q02886|DINB_BACSU FKLYEYNVWANQQIFNRLKELP--------KEIYHQEIQSVFPSISHVLS 47

: ::.* : * * *.. : : *

sp|Q47155|DPO4_ECOLI -RIEPLSLDEAYLDVTDSVHCHG--SATLIAQEIRQTIFNELQLTASAGV 142

tr|Q7NAR2|Q7NAR2_MYCGA NKMEVASIDECYMDVTDILEKNSEISASDLAKKIQKQIYELTQLTVSIGI 149

sp|Q02886|DINB_BACSU ---------HVYLSDLGWIEVFS-----------------------GKTL 65

. *:. . :. . . :

sp|Q47155|DPO4_ECOLI APVKFLAKIASDMNKPNGQFVITPAEVPAFLQTLPLAKIPGVGKVSAAKL 192

tr|Q7NAR2|Q7NAR2_MYCGA SSNLFLAKMASDQNKPNGVYEIWPDEIEEKLWPLEIKKMYLIGSKKIPLL 199

sp|Q02886|DINB_BACSU SDALALAEQLKEQTEAK-----EIEEMEDLFLRLSERYILFLQ-QKEQLN 109

: **: .: .:.: *: : * : : .

sp|Q47155|DPO4_ECOLI EAMGLRTCGDVQKCD-LVMLLKRFGKFGRILWERSQGIDERDVNSER-LR 240

tr|Q7NAR2|Q7NAR2_MYCGA NQLNIKNIGNFAQFENKELLIDIFKNMYWDHYNHAHGKGSDFVDYERNSR 249

sp|Q02886|DINB_BACSU KPLQIQNPS-----------SGIMKTTVSELLPHVVNHG----TYHRGNI 144

: : ::. . : . : . . .*

sp|Q47155|DPO4_ECOLI KSVGVERTMAEDIHHWSECEAIIERLYPELERRLA-----KVKPDLLIAR 285

tr|Q7NAR2|Q7NAR2_MYCGA KSISVSKNIRHKVSDYESLLKLFNDLFDDVYSRLKNHNLLAKNISVSIRT 299

sp|Q02886|DINB_BACSU TAMLRQAGYASAPTDYG--LYLFMTKTEKA-------------------- 172

.:: . .: :: .

sp|Q47155|DPO4_ECOLI QGVKLKFDDFQQTTQEH---------VWPRLNKADLIATARKTWD----- 321

tr|Q7NAR2|Q7NAR2_MYCGA EKVRSLSFSFKQYSDKKTLFYNKAIDLFERLHNNQLVANISISFGNITNK 349

sp|Q02886|DINB_BACSU --------------------------------------------------

sp|Q47155|DPO4_ECOLI ------------ERRGGRGVRLVGLHVTLLDPQMERQLVLGL-------- 351

tr|Q7NAR2|Q7NAR2_MYCGA YKFMPNINIYEELSKEQKDTMLLKKIVNQINKEYNKELVNIADDFDYFKF 399

sp|Q02886|DINB_BACSU --------------------------------------------------

sp|Q47155|DPO4_ECOLI --

tr|Q7NAR2|Q7NAR2_MYCGA QK 401

sp|Q02886|DINB_BACSU --

| **Organism** | **Active site** | **Metal binding (Magnesium, Magnesium)** | **Site (Substrate discrimination)** |
| --- | --- | --- | --- |
| E. coli (strain K12) | E104 | 8, 103 | 13 |
| B. subtilis (strain 168) |  |  |  |
| M. gallisepticum (strain R(low / passage 15 / clone 2)) | E109 | 13, 108 | 18 |
